# Supplementary material for: Risk factors during first 1,000 days of life for carotid intima-media thickness in infants, children, and adolescents: A systematic review with meta-analyses
Source: PLoS Med. 2020 Nov 23;17(11):e1003414. doi: 10.1371/journal.pmed.1003414 (PMC7682901; doi:10.1371/journal.pmed.1003414)
Supplement: S1 PRISMA Checklist — (PDF) [file pmed.1003414.s001.pdf]

S1 PRISMA Checklist. Preferred Reporting Items for Systematic Reviews and Meta-Analyses.

	#	Checklist item	Reported in Section
**TITLE**			
Title	1	Identify the report as a systematic review, meta-analysis, or both.	• Title
**ABSTRACT**			
Structured summary	2	Provide a structured summary including, as applicable: background; objectives; data sources; study eligibility criteria, participants, and interventions; study appraisal and synthesis methods; results; limitations; conclusions and implications of key findings; systematic review registration number.	• Abstract
**INTRODUCTION**			
Rationale	3	Describe the rationale for the review in the context of what is already known.	• Introduction, paragraphs 1-3
Objectives	4	Provide an explicit statement of questions being addressed with reference to participants, interventions, comparisons, outcomes, and study design (PICOS).	• Introduction, paragraph 4
**METHODS**			
Protocol and registration	5	Indicate if a review protocol exists, if and where it can be accessed (e.g., Web address), and, if available, provide registration information including registration number.	• Methods, subheading 1 (Protocol development and reporting)
Eligibility criteria	6	Specify study characteristics (e.g., PICOS, length of follow-up) and report characteristics (e.g., years considered, language, publication status) used as criteria for eligibility, giving rationale.	• Methods, subheading 2 (Eligibility criteria)
Information sources	7	Describe all information sources (e.g., databases with dates of coverage, contact with study authors to identify additional studies) in the search and date last searched.	• Methods, subheading 3 (Search strategy)<br>• Methods, subheading 4 (Study selection process)<br>• S1 Table. Strategies for systematic searches: Source (date of search) column<br>• S2 Table. Strategies for supplementary searches: Source

			(date of search) column
Search	8	Present full electronic search strategy for at least one database, including any limits used, such that it could be repeated.	• S1 Table. Strategies for systematic searches<br>• S2 Table. Strategies for supplementary searches
Study selection	9	State the process for selecting studies (i.e., screening, eligibility, included in systematic review, and, if applicable, included in the meta-analysis).	• Methods, subheading 4 (Study selection process)<br>• Methods, subheading 6 (Data analysis), paragraph 2
Data collection process	10	Describe method of data extraction from reports (e.g., piloted forms, independently, in duplicate) and any processes for obtaining and confirming data from investigators.	• Methods, subheading 5 (Data extraction)<br>• Study protocol (BMJ Open 2018;8:e019644. doi: 10.1136/bmjopen-2017-019644)
Data items	11	List and define all variables for which data were sought (e.g., PICOS, funding sources) and any assumptions and simplifications made.	• Methods, subheading 5 (Data extraction), paragraph 1<br>• Methods, subheading 6 (Data analysis), paragraphs 1 and 2<br>• Study protocol (BMJ Open 2018;8:e019644. doi: 10.1136/bmjopen-2017-019644)
Risk of bias in individual studies	12	Describe methods used for assessing risk of bias of individual studies (including specification of whether this was done at the study or outcome level), and how this information is to be used in any data synthesis.	• Methods, subheading 5 (Data extraction), paragraph 2<br>• S7 Table. Criteria for study quality assessment<br>• S3 Table. Criteria for CIMT quality assessment. CIMT Reliability Tool and algorithm of judgement<br>• Study protocol (BMJ Open 2018;8:e019644. doi: 10.1136/bmjopen-2017-019644)

Summary measures	13	State the principal summary measures (e.g., risk ratio, difference in means).	• Methods, subheading 6 (Data analysis), paragraph 2
Synthesis of results	14	Describe the methods of handling data and combining results of studies, if done, including measures of consistency (e.g., I^2) for each meta-analysis.	• Methods, subheading 6 (Data analysis), paragraphs 2 and 3
Risk of bias across studies	15	Specify any assessment of risk of bias that may affect the cumulative evidence (e.g., publication bias, selective reporting within studies).	• Methods, subheading 5 (Data extraction), paragraph 2<br>• Methods, subheading 6 (Data analysis), paragraph 3
Additional analyses	16	Describe methods of additional analyses (e.g., sensitivity or subgroup analyses, meta-regression), if done, indicating which were pre-specified.	• Methods, subheading 6 (Data analysis), paragraph 3<br>• Study protocol (BMJ Open 2018;8:e019644. doi: 10.1136/bmjopen-2017-019644)

RESULTS			
Study selection	17	Give numbers of studies screened, assessed for eligibility, and included in the review, with reasons for exclusions at each stage, ideally with a flow diagram.	• Fig 1. Flow diagram showing the study selection process
Study characteristics	18	For each study, present characteristics for which data were extracted (e.g., study size, PICOS, follow-up period) and provide the citations.	• Table 1. Characteristics of included studies<br>• Table 2. Carotid intima-media thickness (CIMT) measurement characteristics<br>• S4 Table. CIMT Equipment and operators<br>• S5 Table. Association of CIMT with main exposures or interventions types in the first 1000 days of life<br>• S6 Table. Other exposure types in the first 1000 days of life, by level and category of exposure (reported in a single study)
Risk of bias within studies	19	Present data on risk of bias of each study and, if available, any outcome level assessment (see item 12).	• Table 2. Carotid intima-media thickness (CIMT) measurement characteristics<br>• S8 Table. Assessment of study quality for each intervention type in interventional studies<br>• S9 Table. Assessment of study quality for observational studies included in meta-analyses
Results of individual studies	20	For all outcomes considered (benefits or harms), present, for each study: (a) simple summary data for each intervention group (b) effect estimates and confidence intervals, ideally with a forest plot.	• Fig 2. Association of small size for gestational age with carotid intima-media thickness in children<br>• Fig 3. Association of prematurity with carotid intima-media thickness

			in children<br>• Fig 4. Association of (A) assisted reproductive technology conception, (B) maternal diabetes in pregnancy, (C) maternal smoking in pregnancy with carotid intima-media thickness in children<br>• S5 Table. Association of CIMT with main exposures or interventions types in the first 1000 days of life
Synthesis of results	21	Present results of each meta-analysis done, including confidence intervals and measures of consistency.	• Table 3. Summary of findings for each exposure type included in meta-analyses<br>• Fig 2. Association of small size for gestational age with carotid intima-media thickness in children<br>• Fig 3. Association of prematurity with carotid intima-media thickness in children<br>• Fig 4. Association of (A) assisted reproductive technology conception, (B) maternal diabetes in pregnancy, (C) maternal smoking in pregnancy with carotid intima-media thickness in children
Risk of bias across studies	22	Present results of any assessment of risk of bias across studies (see Item 15).	• Results, subheading 2 (CIMT measurement methods)<br>• S1 Fig. Assessment of study quality for each exposure type included in meta-analyses<br>• S2 Fig. Assessment of publication bias for each exposure type included in meta-analyses

Additional analysis	23	Give results of additional analyses, if done (e.g., sensitivity or subgroup analyses, meta-regression [see Item 16]).	• Fig 2. Association of small size for gestational age with carotid intima-media thickness in children.<br>• Fig 3. Association of prematurity with carotid intima-media thickness in children.<br>• Table 4. Subgroup meta-analyses of standardized mean differences (SMD) for the association of birth size for gestational age or gestational age with carotid intima-media thickness.<br>• S3 Fig. Random-effects meta-regression examining the influence of sample size on the association of small size for gestational age with carotid intima-media thickness.
**DISCUSSION**			
Summary of evidence	24	Summarize the main findings including the strength of evidence for each main outcome; consider their relevance to key groups (e.g., healthcare providers, users, and policy makers).	• Author summary, subheading 3 (What do these findings mean?)<br>• Discussion, subheading 1 (Main findings)<br>• Discussion, subheading 3 (Strengths and limitations), paragraph 2<br>• Conclusions
Limitations	25	Discuss limitations at study and outcome level (e.g., risk of bias), and at review-level (e.g., incomplete retrieval of identified research, reporting bias).	• Discussion, subheading 3 (Strengths and limitations), paragraph 2
Conclusions	26	Provide a general interpretation of the results in the context of other evidence, and implications for future research.	• Discussion, subheading 2 (Comparison with other studies)<br>• Discussion, subheading 4 (Implications for research and

			practice)
**FUNDING**			
Funding	27	Describe sources of funding for the systematic review and other support (e.g., supply of data); role of funders for the systematic review.	• Abstract, Methods and Findings<br>• Study protocol (BMJ Open 2018;8:e019644. doi: 10.1136/bmjopen-2017-019644)

From: Moher D, Liberati A, Tetzlaff J, Altman DG, The PRISMA Group (2009). Preferred Reporting Items for Systematic Reviews and Meta-Analyses: The PRISMA Statement. PLoS Med 6(7): e1000097. doi:10.1371/journal.pmed1000097
