# Supplementary material for: Risk factors during first 1,000 days of life for carotid intima-media thickness in infants, children, and adolescents: A systematic review with meta-analyses
Source: PLoS Med. 2020 Nov 23;17(11):e1003414. doi: 10.1371/journal.pmed.1003414 (PMC7682901; doi:10.1371/journal.pmed.1003414)

S1 Fig. Assessment of study quality for each exposure type included in meta-analyses. Abbreviations: ART, assisted reproductive technology.

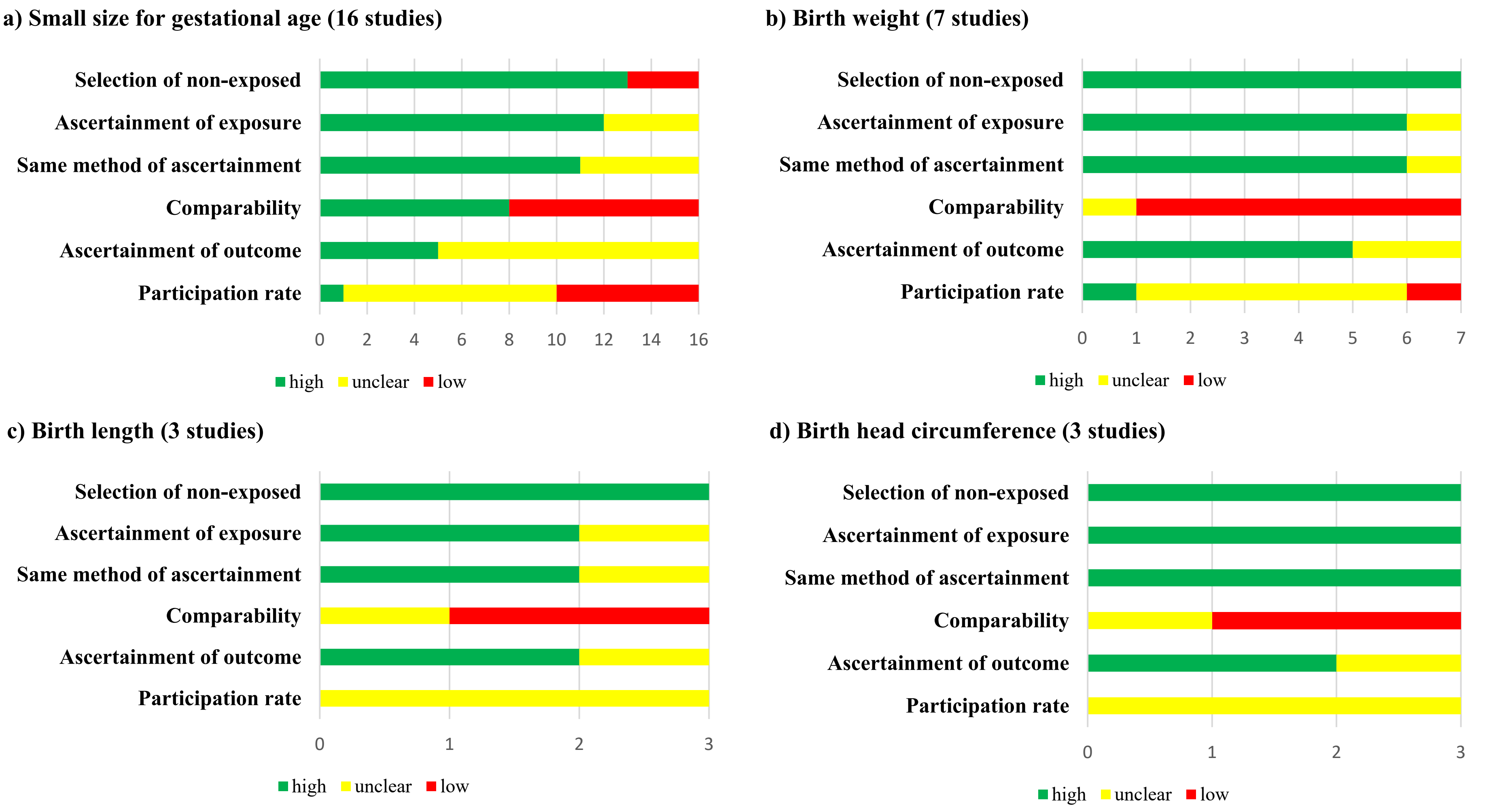

e) Prematurity (7 studies)

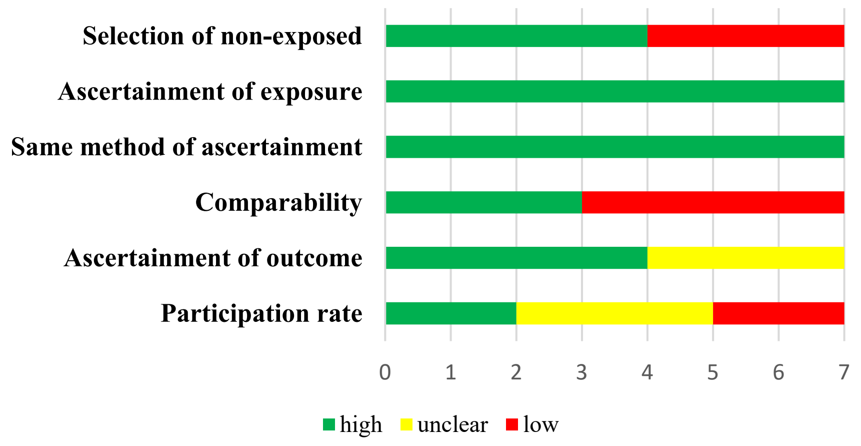

f) ART conception (3 studies)

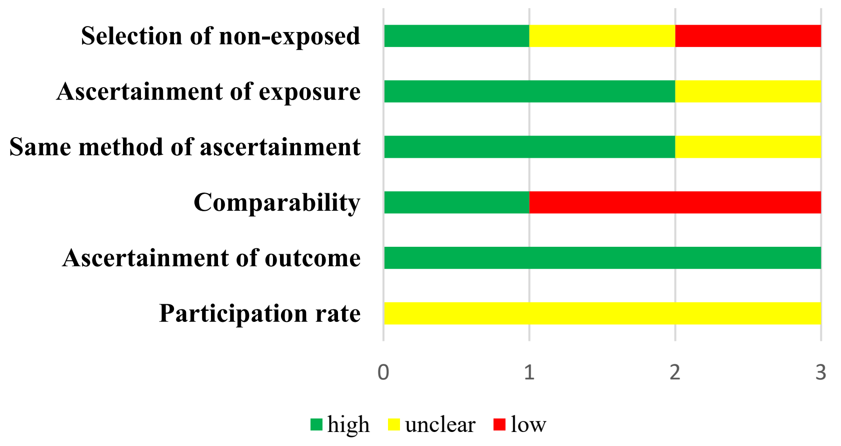

g) Maternal diabetes in pregnancy (3 studies)

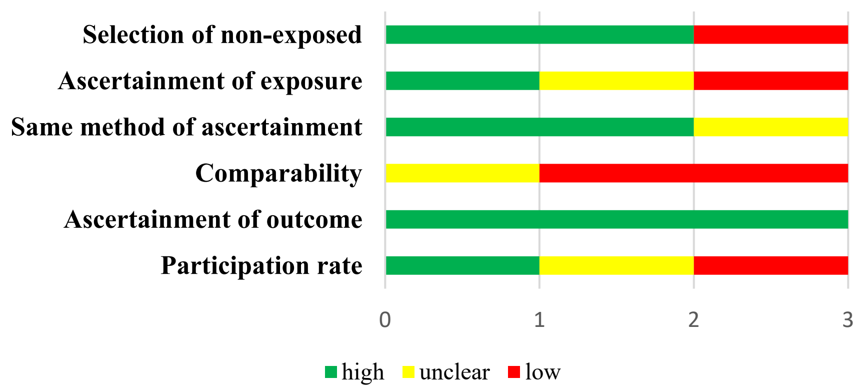

h) Maternal smoking in pregnancy (3 studies)

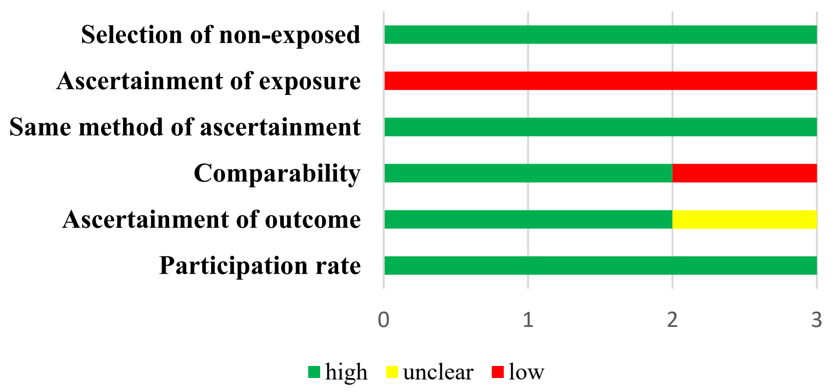

Supplement: S1 Fig — (PDF) [file pmed.1003414.s002.pdf]
