# Supplementary material for: Risk factors during first 1,000 days of life for carotid intima-media thickness in infants, children, and adolescents: A systematic review with meta-analyses
Source: PLoS Med. 2020 Nov 23;17(11):e1003414. doi: 10.1371/journal.pmed.1003414 (PMC7682901; doi:10.1371/journal.pmed.1003414)

S2 Fig. Assessment of small-study effects, including publication bias, for each exposure type included in meta-analyses. Funnel plots of standardized mean difference (binary exposure variable) or Fischer's z correlation (continuous exposure variable) against its standard error. Abbreviations: ART, assisted reproductive technology. * p-value for small study effects from Egger's test, computed only for the exposure types with 10 or more studies included in meta-analysis.

a) Small size for gestational age (16 studies) *

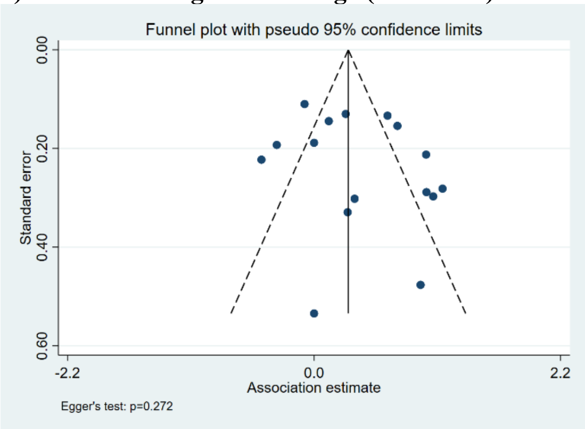

b) Birth weight (7 studies)

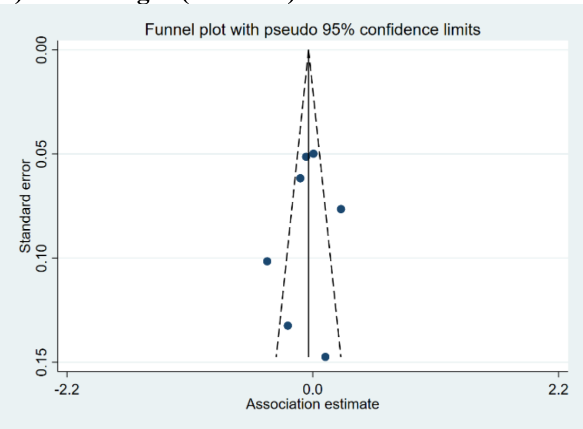

c) Birth length (3 studies)

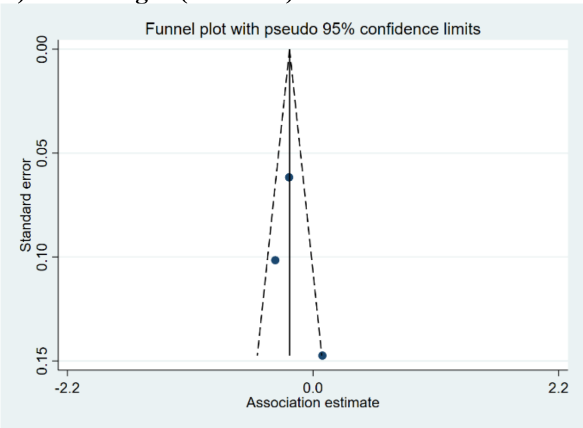

d) Birth head circumference (3 studies)

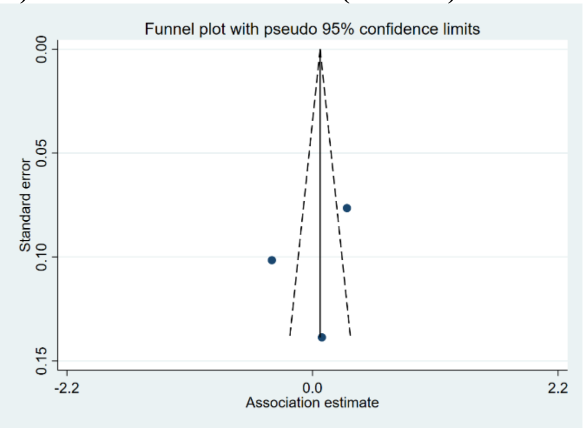

e) Prematurity (7 studies)

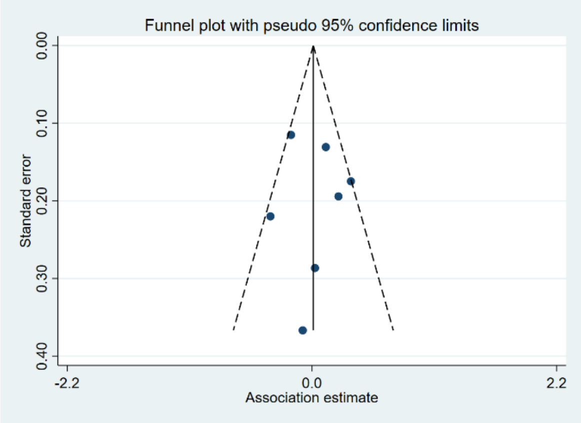

f) ART conception (3 studies)

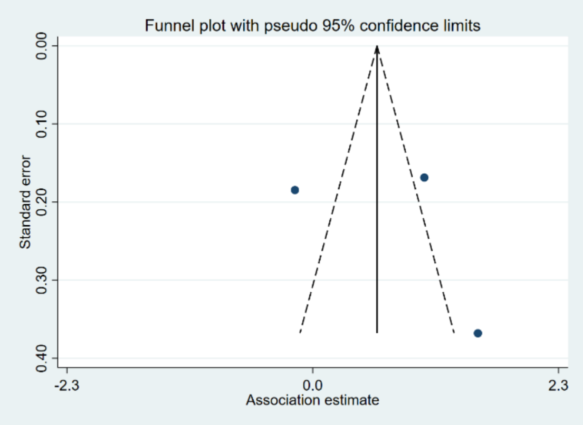

g) Maternal diabetes in pregnancy (3 studies)

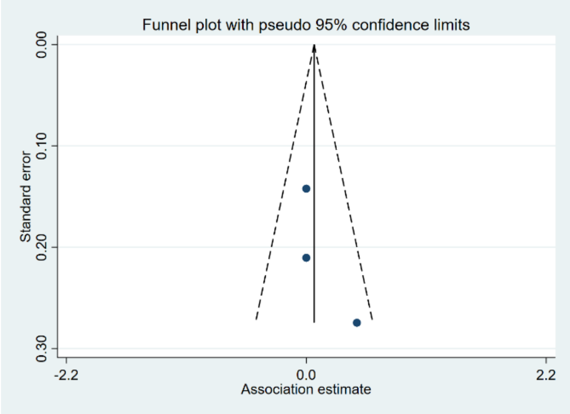

h) Maternal smoking in pregnancy (3 studies)

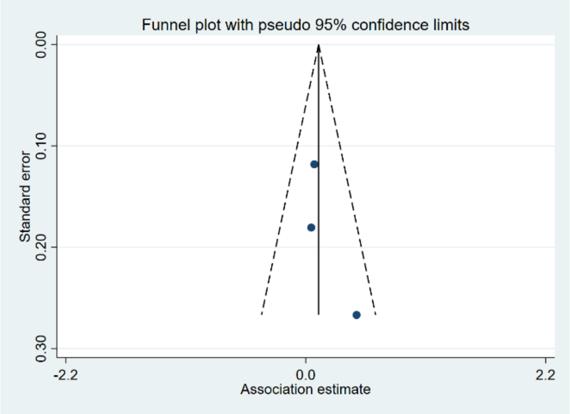

Supplement: S2 Fig — (PDF) [file pmed.1003414.s003.pdf]
