# Supplementary material for: Risk factors during first 1,000 days of life for carotid intima-media thickness in infants, children, and adolescents: A systematic review with meta-analyses
Source: PLoS Med. 2020 Nov 23;17(11):e1003414. doi: 10.1371/journal.pmed.1003414 (PMC7682901; doi:10.1371/journal.pmed.1003414)

S3 Fig. Random-effects meta-regression examining the influence of sample size on the association of small size for gestational age with carotid intima-media thickness. Bubble plot with fitted meta-regression line. The circles represent the association estimate (standardized mean difference) from each study, sized according to the random-effects weights. Number of studies: 16. Residual between-study variance (tau^2): 0.21. Residual variation due to heterogeneity (I^2_{res}): 81%. Between-study variance explained by the covariate sample size (Adjusted R^2): 2%.

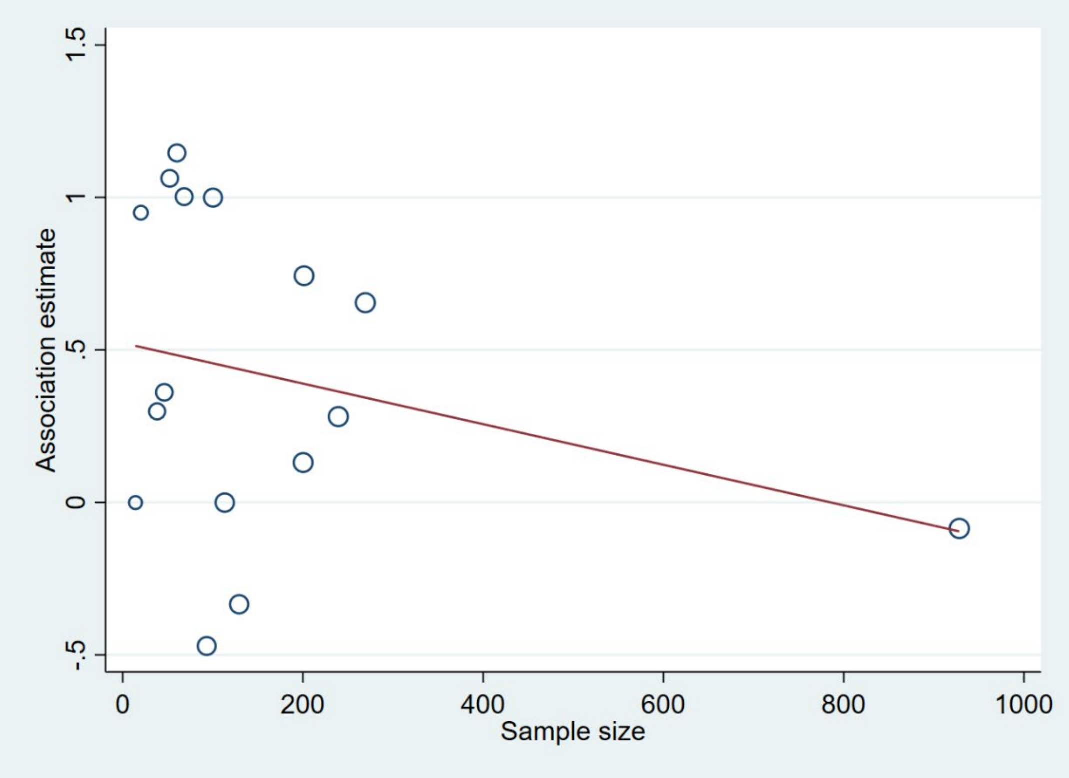

Supplement: S3 Fig — (PDF) [file pmed.1003414.s004.pdf]
