# Supplementary material for: Risk factors during first 1,000 days of life for carotid intima-media thickness in infants, children, and adolescents: A systematic review with meta-analyses
Source: PLoS Med. 2020 Nov 23;17(11):e1003414. doi: 10.1371/journal.pmed.1003414 (PMC7682901; doi:10.1371/journal.pmed.1003414)
Supplement: S1 Table — (PDF) [file pmed.1003414.s005.pdf]

S1 Table. Strategies for systematic searches [1].

Source (date of search)	Search strategy
EMBASE (18 March 2019)	1. baby:ab,ti OR babies:ab,ti OR preterm*:ab,ti OR 'pre term*':ab,ti OR prematur*:ab,ti OR newborn*:ab,ti OR infan*:ab,ti OR toddler*:ab,ti OR kindergart*:ab,ti OR kid:ab,ti OR kids:ab,ti OR boy*:ab,ti OR girl*:ab,ti OR preschool*:ab,ti OR 'pre school*':ab,ti OR child*:ab,ti OR school*:ab,ti OR preteen*:ab,ti OR prepube*:ab,ti OR preadolescen*:ab,ti OR highschool*:ab,ti OR 'high school*':ab,ti OR student*:ab,ti OR adolescen*:ab,ti OR teen*:ab,ti OR pube*:ab,ti OR youngster*:ab,ti OR youth*:ab,ti OR pediatric*:ab,ti OR paediatric*:ab,ti OR peadiatric*:ab,ti OR neonat*:ab,ti OR perinat*:ab,ti OR offspring:ab,ti OR descendant*:ab,ti OR child/exp OR adolescent/exp OR juvenile/de OR prematurity/syn OR kindergarten/de OR pediatrics/de OR neonatology/de OR perinatology/de OR progeny/de<br>2. (((intima* OR wall OR arter*) NEAR/5 (thick* OR complex*)):ti,ab) OR ‘artery wall’/de OR ‘artery intima proliferation’/de OR intima/de OR ‘artery intima’/de OR ‘artery media’/de OR ‘arterial wall thickness’/de<br>3. carotid:ab,ti OR ‘arteria carotis’:ab,ti OR ‘carotid artery’/syn OR ‘carotid artery disease’/de<br>4. atherosclero*:ab,ti OR atherosclerosis/exp OR arteriosclero*:ab,ti OR arteriosclerosis/exp OR ‘end organ damage’:ab,ti OR ‘target organ damage’:ab,ti OR ‘cardiovascular disease’/de<br>5. ultrasound:ab,ti OR echograph*:ab,ti OR ultrasonograph*:ab,ti OR sonograph*:ab,ti OR echography/syn<br>6. #2 AND #3<br>7. #3 AND #4 AND #5<br>8. #6 OR #7<br>9. #1 AND #8<br>10. #9 NOT ([animals]/lim NOT [humans]/lim)
MEDLINE (18 March 2019)	1. baby[tiab] OR babies[tiab] OR preterm*[tiab] OR pre-term*[tiab] OR prematur*[tiab] OR newborn*[tiab] OR infan*[tiab] OR infant[mh] OR toddler*[tiab] OR kindergart*[tiab] OR kid[tiab] OR kids[tiab] OR boy*[tiab] OR girl*[tiab] OR preschool*[tiab] OR pre-school*[tiab] OR child*[tiab] OR child[mh] OR school*[tiab] OR preteen*[tiab] OR prepube*[tiab] OR preadolescen*[tiab] OR highschool*[tiab] OR high-school*[tiab] OR student*[tiab] OR adolescen*[tiab] OR adolescent[mh] OR teen*[tiab] OR pube*[tiab] OR youngster*[tiab] OR youth*[tiab] OR pediatric*[tiab] OR paediatric*[tiab] OR peadiatric*[tiab] OR pediatrics[mh] OR neonat*[tiab] OR perinat*[tiab] OR offspring[tiab] OR descendant*[tiab]<br>2. “intima media thickness”[tiab] OR “intima media thickening”[tiab] OR “intimal medial thickness”[tiab] OR “intimal medial thickening”[tiab] OR “intimal media thickness”[tiab] OR “intimal media thickening”[tiab] OR “intima medial thickness”[tiab] OR “intima medial thickening”[tiab] OR “intima media complex”[tiab] OR “intimal medial complex”[tiab] OR “intimal media complex”[tiab] OR “intimamedia thickness”[tiab] OR “wall thickness”[tiab] OR “wall thickening”[tiab] OR “arterial thickness”[tiab] OR “artery thickness”[tiab] OR “artery wall thickness”[tiab] OR “arterial wall thickness”[tiab] OR “intimal thickening”[tiab] OR “tunica intima/diagnostic imaging”[mh] OR “tunica media/diagnostic imaging”[mh]<br>3. carotid[tiab] OR “arteria carotis”[tiab] OR “carotid arteries”[mh] OR “carotid artery diseases”[mh:noexp]

	4. atherosclero*[tiab] OR atherosclerosis[mh] OR arteriosclero*[tiab] OR arteriosclerosis[mh] OR "end organ damage"[tiab] OR "target organ damage"[tiab] OR "cardiovascular diseases"[mh:noexp]<br>5. ultrasound[tiab] OR echograph*[tiab] OR ultrasonograph*[tiab] OR sonograph*[tiab] OR ultrasonography[mh]<br>6. #2 AND #3<br>7. #3 AND #4 AND #5<br>8. "carotid intima-media thickness"[mh] OR "carotid arteries/diagnostic imaging"[mh]<br>9. #6 OR #7 OR #8<br>10. #1 AND #9<br>#10 NOT (animals[mh] NOT humans[mh])
CENTRAL (18 March 2019) Limits: Database of trials	1. baby:ti,ab,kw OR babies:ti,ab,kw OR preterm*:ti,ab,kw OR pre-term*:ti,ab,kw OR prematur*:ti,ab,kw OR newborn*:ti,ab,kw OR infan*:ti,ab,kw OR toddler*:ti,ab,kw OR kindergart*:ti,ab,kw OR kid:ti,ab,kw OR kids:ti,ab,kw OR boy*:ti,ab,kw OR girl*:ti,ab,kw OR preschool*:ti,ab,kw OR pre-school*:ti,ab,kw OR child*:ti,ab,kw OR school*:ti,ab,kw OR preteen*:ti,ab,kw OR prepube*:ti,ab,kw OR preadolescen*:ti,ab,kw OR highschool*:ti,ab,kw OR high-school*:ti,ab,kw OR student*:ti,ab,kw OR adolescen*:ti,ab,kw OR teen*:ti,ab,kw OR pube*:ti,ab,kw OR youngster*:ti,ab,kw OR youth*:ti,ab,kw OR pediatric*:ti,ab,kw OR paediatric*:ti,ab,kw OR peadiatric*:ti,ab,kw OR neonat*:ti,ab,kw OR perinat*:ti,ab,kw OR offspring:ti,ab,kw OR descendant*:ti,ab,kw<br>2. (((intima* OR wall OR arter*) NEAR/5 (thick* OR complex*)):ti,ab,kw) OR "tunica intima":ti,ab,kw OR "tunica media":ti,ab,kw<br>3. carotid:ti,ab,kw OR "arteria carotis":ti,ab,kw<br>4. atherosclero*:ti,ab,kw OR arteriosclero*:ti,ab,kw OR "end organ damage":ti,ab,kw OR "target organ damage":ti,ab,kw OR (cardiovascular NEXT disease*):ti,ab,kw<br>5. ultrasound:ti,ab,kw OR echograph*:ti,ab,kw OR ultrasonograph*:ti,ab,kw OR sonograph*:ti,ab,kw<br>6. #2 AND #3<br>7. #3 AND #4 AND #5<br>8. #6 OR #7<br>9. #1 AND #8

References

1. Epure AM, Leyvraz M, Mivelaz Y, Di Bernardo S, da Costa BR, Chiolero A, et al. Risk factors and determinants of carotid intima-media thickness in children: protocol for a systematic review and meta-analysis. BMJ open. 2018;8(6):e019644. doi: 10.1136/bmjopen-2017-019644.
