# Supplementary material for: Risk factors during first 1,000 days of life for carotid intima-media thickness in infants, children, and adolescents: A systematic review with meta-analyses
Source: PLoS Med. 2020 Nov 23;17(11):e1003414. doi: 10.1371/journal.pmed.1003414 (PMC7682901; doi:10.1371/journal.pmed.1003414)
Supplement: S2 Table — (PDF) [file pmed.1003414.s006.pdf]

S2 Table. Strategies for supplementary searches.

**Source (date of search)**	**Search Strategy**
www.clinicaltrials.gov (02 July 2018)	(baby OR newborn OR infant OR child OR adolescent OR pregnancy OR gestational OR prenatal OR antenatal OR utero OR fetal OR birth OR perinatal) AND carotid AND (intima OR intimal) AND (media OR medial) AND (thickness OR thickening OR complex)
www.isrctn.com (10 July 2018)	(baby OR newborn OR infant OR child OR adolescent OR pregnancy OR gestational OR prenatal OR antenatal OR utero OR fetal OR foetal OR birth OR perinatal) AND carotid AND intima AND media AND (thickness OR thickening OR complex)
www.clinicaltrialsregister.eu (10 July 2018)	(baby OR newborn OR infant OR child OR adolescent OR pregnancy OR gestational OR prenatal OR antenatal OR utero OR fetal OR birth OR perinatal) AND carotid AND (intima OR intimal) AND (media OR medial) AND (thickness OR thickening OR complex)
Google Scholar (18 June 2018)	newborn|newborns|infant|infants|child|children|adolescent|adolescents carotid "intima|intimal media|medial thickness|thickening|complex" pregnancy|gestational|prenatal|antenatal|utero|fetal|foetal|birth|infancy|perinatal
Reference lists	Reference lists of 37 full-texts included after systematic searches and 4 reviews on the topic.
Web of Science (19 June 2018)	Forward citation tracking was performed based on all full-texts included after systematic searches. Limited to references published between 2016 and 2018.
