# Supplementary material for: Risk factors during first 1,000 days of life for carotid intima-media thickness in infants, children, and adolescents: A systematic review with meta-analyses
Source: PLoS Med. 2020 Nov 23;17(11):e1003414. doi: 10.1371/journal.pmed.1003414 (PMC7682901; doi:10.1371/journal.pmed.1003414)
Supplement: S3 Table — (PDF) [file pmed.1003414.s007.pdf]

S3 Table. Criteria for CIMT quality assessment. CIMT Reliability Tool and algorithm of judgement [1].

No.	DOMAIN	RELIABILITY		
**1**	**Image acquisition** (Site of measurement)	Higher	Lower	Unclear
		• Far-wall of the CCA;<br>• Far-wall of CCA & CB & ICA.	• Otherwise (e.g., near-wall measurements of any segment or combinations of segments; combined near and far-wall measurements of any segment or combinations of segments).	• Insufficient information to permit judgment.
**2**	**Image analysis**	Higher	Lower	Unclear
		• Automatic or semi-automatic edge detection approach;<br>• Manual tracing of lines/unclear edge detection + automatic analysis of the CIMT interfaces over a specific length + at least two separate images/frames of the same site included in the CIMT calculation.	• Otherwise (e.g., manual cursor placement + point-to-point measurements within a region of interest; manual tracing of lines + automatic analysis of the CIMT interfaces over a specific length + a single image/frame of the same site included in the CIMT calculation).	• Insufficient information to permit judgment.
**3**	**Reproducibility assessment**	Higher	Lower	Unclear
		• Yes	• No	• Insufficient information to permit judgment.

Abbreviations: CIMT, carotid intima-media thickness; CCA, Common carotid artery; ICA, Internal carotid artery; CB, Carotid bifurcation.

References

1. Epure AM, Leyvraz M, Mivelaz Y, Di Bernardo S, da Costa BR, Chiolero A, et al. Risk factors and determinants of carotid intima-media thickness in children: protocol for a systematic review and meta-analysis. BMJ open. 2018;8(6):e019644. doi: 10.1136/bmjopen-2017-019644.
