# Supplementary material for: Risk factors during first 1,000 days of life for carotid intima-media thickness in infants, children, and adolescents: A systematic review with meta-analyses
Source: PLoS Med. 2020 Nov 23;17(11):e1003414. doi: 10.1371/journal.pmed.1003414 (PMC7682901; doi:10.1371/journal.pmed.1003414)
Supplement: S4 Table — (PDF) [file pmed.1003414.s008.pdf]

S4 Table. CIMT Equipment and operators.

Study name or Author, publication year	US device	US mode	Transducer array, frequency (MHz)	Operators
Gale [1], 2006	Acuson XP128	N/S	linear, 7	N/S
Gale [2], 2008	Acuson XP128	N/S	linear, 7	N/S
Ayer [3], 2009 (a)	Terason 3000; Teratek	B-mode	linear, 5-12	N/S
Ayer [4], 2009 (b)	Terason 3000; Teratek	B-mode	linear, 5-12	N/S
Ayer [5], 2011	Terason 3000; Teratek	B-mode	linear, 5-12	N/S
Skilton [6], 2012	Terason 3000; Teratek	B-mode	linear, 5-12	N/S
Skilton [7], 2013	Terason 3000; Teratek	B-mode	linear, 5-12	N/S
Crispi [8, 9], 2010	Siemenes Sonoline Antares	N/S	linear, 13	experienced physicians
Rodriguez-Lopez [10], 2016	Siemenes Sonoline Antares	N/S	linear, 13	N/S
Trevisanuto [11], 2010	Technos system (ESAOTE, Biomedica)	N/S	linear, 7.5	angiologists
Evelein [12], 2011	N/S	RF-mode	linear, N/S	one of the investigators in the study and a trained research nurse; the Vascular Imaging Center provided assistance in the vascular measurements
Geerts [13], 2012	N/S	RF-mode	linear, N/S	one of the investigators in the study and a trained research nurse; the Vascular Imaging Center provided assistance in the vascular measurements
Evelein [14], 2013	N/S	RF-mode	linear, N/S	two of the investigators in the study and a trained research nurse; the Vascular Imaging Center provided assistance in the vascular measurements
Pluymen [15], 2017	N/S	RF-mode	linear, N/S	trained pediatric research nurse
Atabek [16], 2011	Phillips Sonos 5500	B-mode	N/S, 3.5	clinician

Dratva [17], 2013	portable Biosound MyLab 25	B-mode	linear, 10	imaging specialist from the USC Atherosclerosis Research Unit, Core Imaging and Reading Center
Breton, 2016 [18] (a)	portable Biosound MyLab 25	B-mode	linear, 10	N/S
Breton, 2016 [19] (b)	portable Biosound MyLab 25	B-mode	linear, 10	physician-imaging specialist from the USC Atherosclerosis Research Unit (ARU) Core Imaging and Reading Center (CIRC)
Schubert [20], 2013	GE Vingmed Vivid 7	M-mode	phased, 4-10.5	N/S
Valenzuela-Alcaraz [21, 22], 2019	Vivid q (General Electric Healthcare)	N/S	linear, 3-10	skilled sonographer
Lee [23], 2014	N/S	B-mode	N/S, N/S	experienced sonographers
Morsing [24], 2014	Acuson Sequoia C512	N/S	N/S, 15	N/S
Stergiotou [25], 2014	Siemens Sonoline Antares	N/S	linear, 13	skilled sonographers
Gruszfeld [26], 2015	Brussels: Siemens Sonoline Antares; Liege: ATL Phillips HDI 5000; Germany: Phillips iE33; Italy: GE Logiq 9; Poland: Vivid Pro 7; Reus: Acuson Antares System; Tarragona: Acuson Sequoia 512 TM	B-mode	linear, multiple between 5-15	sonographer(s): trained member of the medical stuff in each of the 11 study sites; reader(s): single trained physician from the Children's Memorial Health Institute in Warsaw, Poland
Sebastiani [27], 2016	MyLab 125	N/S	linear, 12	one investigator in the study
Liu [28], 2017	Vivid I	B-mode	linear, 10	sonographer(s): trained technicians; reader(s): 1 technician that did a preliminary processing of recording and the measurement by 6 trained raters
Mohlkert [29], 2017	N/S	B-mode	N/S, N/S	1 experienced ultrasound examiner at each of the 3 centers with vascular measurements
Tzschoppe [30], 2017	GE logic 9, GE Healthcare	B-mode	linear, 9	N/S
Carreras-Badosa [31], 2018	MyLabTM25, Esaote, Italy	N/S	linear, 7.5-12	N/S
Chen [32], 2019	HDI 5000 apparatus (ATL; Philips, Bothell, WA)	B-mode	linear, 5.5-12.5 mHz	experienced senior ultrasound specialist

Prins-Van Ginkel [33], 2019	Panasonic CardioHealth Station (Panasonic Healthcare)	N/S	N/S, N/S	trained research staff
Sebastiani [34], 2019	MyLab 125	N/S	linear, 12	N/S
Sundholm [35], 2019	Vevo 770 system and Vevo MD system (VisualSonics, Toronto, Canada)	N/S	N/S, 35/55	sonographer(s): skilled; reader(s): experienced
Jouret [36], 2011	Philips Sonos 5550	B-mode	N/S, N/S	one study investigator rigorously trained
Scherrer [37, 38], 2012	Acuson Sequoia 512 C	B-mode	linear, 8-14	N/S
de Arriba [39], 2013	N/S	N/S	linear, 8	experienced observer
Maurice [40], 2014	Phillips iE33	B-mode	N/S, 11	N/S
Xu [41], 2014	Phillips HD11 XE	M-mode	linear, 7-9	N/S
Putra [42], 2015	General Electric Logic E	N/S	N/S, 7-12	ultrasound technician
Sodhi [43], 2015	Philips HD 11	B-mode	linear, 3-12	sonographer(s): radiologist; reader(s): pediatric radiologists
Ciccone [44], 2016	N/S	N/S	N/S, 7.5	physician
Faienza [45], 2016	Philips Sonos 5500	N/S	N/S, 7.5	physician
Olander [46], 2016	Vevo 770	B-mode	N/S, 35/55	N/S
Dilli [47], 2017	General Electric Logic 5 (Ottowa, Canada)	N/S	linear, 7.5	skilled sonographer, with more than 5 years of experience in the field
Stock [48], 2018	N/S	N/S	linear, high frequency	N/S
Wilde [49], 2018	N/S	RF-mode	linear, N/S	medical doctor
Muñiz Fontán [50], 2019	Vivid I (General Electrics, Haifa, Israel)	B-mode	linear, 12	pediatric cardiologist

References

1. Gale CR, Jiang B, Robinson SM, Godfrey KM, Law CM, Martyn CN. Maternal diet during pregnancy and carotid intima-media thickness in children. Arteriosclerosis, thrombosis, and vascular biology. 2006;26(8):1877-82. doi: 10.1161/01.ATV.0000228819.13039.b8.
2. Gale CR, Robinson SM, Harvey NC, Javaid MK, Jiang B, Martyn CN, et al. Maternal vitamin D status during pregnancy and child outcomes. European Journal of Clinical Nutrition. 2008;62(1):68-77. doi: 10.1038/sj.ejcn.1602680.
3. Ayer JG, Harmer JA, Nakhla S, Xuan W, Ng MKC, Raitakari OT, et al. HDL-cholesterol, blood pressure, and asymmetric dimethylarginine are significantly associated with arterial wall thickness in children. Arteriosclerosis, thrombosis, and vascular biology. 2009;29(6):943-9. doi: 10.1161/ATVBAHA.109.184184.
4. Ayer JG, Harmer JA, Xuan W, Toelle B, Webb K, Almqvist C, et al. Dietary supplementation with n-3 polyunsaturated fatty acids in early childhood: Effects on blood pressure and arterial structure and function at age 8 y. American Journal of Clinical Nutrition. 2009;90(2):438-46. doi: 10.3945/ajcn.2009.27811.
5. Ayer JG, Belousova E, Harmer JA, David C, Marks GB, Celermajer DS. Maternal cigarette smoking is associated with reduced high-density lipoprotein cholesterol in healthy 8-year-old children. European heart journal. 2011;32(19):2446-53. doi: 10.1093/eurheartj/ehr174.
6. Skilton MR, Ayer JG, Harmer JA, Webb K, Leeder SR, Marks GB, et al. Impaired fetal growth and arterial wall thickening: A randomized trial of omega-3 supplementation. Pediatrics. 2012;129(3):e698-e703. doi: 10.1542/peds.2011-2472.
7. Skilton MR, Marks GB, Ayer JG, Garden FL, Garnett SP, Harmer JA, et al. Weight gain in infancy and vascular risk factors in later childhood. Pediatrics. 2013;131(6):e1821-e8. doi: 10.1542/peds.2012-2789.
8. Crispi F, Bijnens B, Figueras F, Bartrons J, Eixarch E, Le Noble F, et al. Fetal growth restriction results in remodeled and less efficient hearts in children. Circulation. 2010;121(22):2427-36. doi: 10.1161/circulationaha.110.937995.
9. Crispi F, Figueras F, Cruz-Lemini M, Bartrons J, Bijnens B, Gratacos E. Cardiovascular programming in children born small for gestational age and relationship with prenatal signs of severity. American Journal of Obstetrics and Gynecology. 2012;207(2):121.e1-.e9. doi: 10.1016/j.ajog.2012.05.011.
10. Rodriguez-Lopez M, Osorio L, Acosta-Rojas R, Figueras J, Cruz-Lemini M, Figueras F, et al. Influence of breastfeeding and postnatal nutrition on cardiovascular remodeling induced by fetal growth restriction. Pediatric research. 2016;79(1):100-6. doi: 10.1038/pr.2015.182.
11. Trevisanuto D, Avezzú F, Cavallin F, Doglioni N, Marzolo M, Verlato F, et al. Arterial wall thickness and blood pressure in children who were born small for gestational age: Correlation with umbilical cord high-sensitivity C-reactive protein. Archives of disease in childhood. 2010;95(1):31-4. doi: 10.1136/adc.2008.150326.
12. Evelein AMV, Geerts CC, Visseren FLJ, Bots ML, Van Der Ent CK, Grobbee DE, et al. The association between breastfeeding and the cardiovascular system in early childhood. American Journal of Clinical Nutrition. 2011;93(4):712-8. doi: 10.3945/ajcn.110.002980.
13. Geerts CC, Bots ML, Van Der Ent CK, Grobbee DE, Uiterwaal CSPM. Parental smoking and vascular damage in their 5-year-old children. Pediatrics. 2012;129(1):45-54. doi: 10.1542/peds.2011-0249.

14. Evelein AMV, Visseren FLJ, Van Der Ent CK, Grobbee DE, Uiterwaal CSPM. Excess early postnatal weight gain leads to thicker and stiffer arteries in young children. Journal of Clinical Endocrinology and Metabolism. 2013;98(2):794-801. doi: 10.1210/jc.2012-3208.
15. Pluymen LPM, Dalmeijer GW, Smit HA, Uiterwaal C, van der Ent CK, van Rossem L. Long-chain polyunsaturated fatty acids in infant formula and cardiovascular markers in childhood. Matern Child Nutr. 2017;14(2):e12523. doi: 10.1111/mcn.12523.
16. Atabek ME, Çağan HH, Eklioğlu BS, Oran B. Absence of increase in carotid artery Intima-Media thickness in infants of diabetic mothers. JCRPE Journal of Clinical Research in Pediatric Endocrinology. 2011;3(3):144-8. doi: 10.4274/jcrpe.v3i3.28.
17. Dratva J, Breton CV, Hodis HN, Mac KWJ, Salam MT, Zemp E, et al. Birth weight and carotid artery intima-media thickness. Journal of Pediatrics. 2013;162(5):906-11.e2. doi: 10.1016/j.jpeds.2012.10.060.
18. Breton CV, Yao J, Millstein J, Gao L, Siegmund KD, Mack W, et al. Prenatal air pollution exposures, DNA methyl transferase genotypes, and associations with newborn line1 and Alu methylation and childhood blood pressure and carotid intima-media thickness in the children's health study. Environmental Health Perspectives. 2016;124(12):1905-12. doi: 10.1289/EHP181.
19. Breton CV, Gao L, Yao J, Siegmund KD, Lurmann F, Gilliland F. Particulate matter, the newborn methylome, and cardio-respiratory health outcomes in childhood. Environ Epigenet. 2016;2(2):dvw005. doi: 10.1093/eep/dvw005.
20. Schubert U, Müller M, Abdul-Khaliq H, Norman M, Bonamy AKE. Relative intima-media thickening after preterm birth. Acta Paediatrica, International Journal of Paediatrics. 2013;102(10):965-9. doi: 10.1111/apa.12355.
21. Valenzuela-Alcaraz B, Serafini A, Sepulveda-Martinez A, Casals G, Rodriguez-Lopez M, Garcia-Otero L, et al. Postnatal persistence of fetal cardiovascular remodelling associated with assisted reproductive technologies: a cohort study. Bjog. 2019;126(2):291-8. doi: 10.1111/1471-0528.15246.
22. Valenzuela-Alcaraz B, Crispi F, Bijnens B, Cruz-Lemini M, Creus M, Sitges M, et al. Assisted reproductive technologies are associated with cardiovascular remodeling in utero that persists postnatally. Circulation. 2013;128(13):1442-50. doi: 10.1161/circulationaha.113.002428.
23. Lee H, Dichtl S, Mormanova Z, Dalla Pozza R, Genzel-Boroviczeny O. In adolescence, extreme prematurity is associated with significant changes in the microvasculature, elevated blood pressure and increased carotid intima - Media thickness. Archives of Disease in Childhood: Education and Practice Edition. 2014;99(10):907-11. doi: 10.1136/archdischild-2013-304074.
24. Morsing E, Liuba P, Fellman V, Maršál K, Brodszki J. Cardiovascular function in children born very preterm after intrauterine growth restriction with severely abnormal umbilical artery blood flow. European journal of preventive cardiology. 2014;21(10):1257-66. doi: 10.1177/2047487313486044.
25. Stergiotou I, Crispi F, Valenzuela-Alcaraz B, Cruz-Lemini M, Bijnens B, Gratacos E. Aortic and carotid intima-media thickness in term small-for-gestational-age newborns and relationship with prenatal signs of severity. Ultrasound in obstetrics & gynecology : the official journal of the International Society of Ultrasound in Obstetrics and Gynecology. 2014;43(6):625-31. doi: 10.1002/uog.13245.
26. Gruszfeld D, Weber M, Nowakowska-Rysz M, Janas R, Kozlik-Feldmann R, Xhonneux A, et al. Protein intake in infancy and carotid intima media thickness at 5 years - A secondary analysis from a randomized trial for the European childhood obesity study group. Annals of Nutrition and Metabolism. 2015;66(1):51-9. doi: 10.1159/000369980.

27. Sebastiani G, Díaz M, Bassols J, Aragonés G, López-Bermejo A, de Zegher F, et al. The sequence of prenatal growth restraint and post-natal catch-up growth leads to a thicker intima-media and more pre-peritoneal and hepatic fat by age 3–6 years. Pediatric Obesity. 2016;11(4):251-7. doi: 10.1111/ijpo.12053.
28. Liu RS, Mensah FK, Carlin J, Edwards B, Ranganathan S, Cheung M, et al. Socioeconomic Position Is Associated With Carotid Intima-Media Thickness in Mid-Childhood: The Longitudinal Study of Australian Children. Journal of the American Heart Association. 2017;6(8). doi: 10.1161/jaha.117.005925.
29. Mohlkert LA, Hallberg J, Broberg O, Hellström M, Pegelow Halvorsen C, Sjöberg G, et al. Preterm arteries in childhood: Dimensions, intima-media thickness, and elasticity of the aorta, coronaries, and carotids in 6-y-old children born extremely preterm. Pediatric research. 2017;81(2):299-306. doi: 10.1038/pr.2016.212.
30. Tzschoppe A, Von Kries R, Struwe E, Rascher W, Dörr HG, Jüngert J, et al. Intrauterine Growth Restriction (IUGR) Induces Signs of Subclinical Atherosclerosis in 6-Year-Old Infants Despite Absence of Excessive Growth. Klinische Padiatrie. 2017;229(4):209-15. doi: 10.1055/s-0043-104528.
31. Carreras-Badosa G, Armero-Bujaldón C, Solé-Amat L, Prats-Puig A, Díaz-Roldán F, Soriano-Rodriguez P, et al. Serum 25-hydroxyvitamin D and cardiovascular disease risk factors in women with excessive weight gain during pregnancy and in their offspring at age 5-6 years. International Journal of Obesity. 2018;42(5):1019-28. doi: 10.1038/s41366-018-0101-6.
32. Chen L, Guilmette J, Luo ZC, Cloutier A, Wang WJ, Yang MN, et al. Placental 11beta-HSD2 and Cardiometabolic Health Indicators in Infancy. Diabetes care. 2019. doi: 10.2337/dc18-2041.
33. Prins-Van Ginkel AC, Bruijning-Verhagen PCJ, Wijga AH, Bots ML, Gehring U, Van Der Hoek W, et al. Childhood infections and common carotid intima media thickness in adolescence. Epidemiology and Infection. 2019;147. doi: 10.1017/S095026881800287X.
34. Sebastiani G, García-Beltran C, Pie S, Guerra A, López-Bermejo A, de Toledo JS, et al. The sequence of prenatal growth restraint and postnatal catch-up growth: normal heart but thicker intima-media and more pre-peritoneal fat in late infancy. Pediatric Obesity. 2019;14(3). doi: 10.1111/ijpo.12476.
35. Sundholm JKM, Litwin L, Rönö K, Koivusalo SB, Eriksson JG, Sarkola T. Maternal obesity and gestational diabetes: Impact on arterial wall layer thickness and stiffness in early childhood - RADIEL study six-year follow-up. Atherosclerosis. 2019. doi: 10.1016/j.atherosclerosis.2019.01.037.
36. Jouret B, Dulac Y, Bassil Eter R, Taktak A, Cristini C, Lounis N, et al. Endothelial function and mechanical arterial properties in children born small for gestational age: comparison with obese children. Hormone research in paediatrics. 2011;76(4):240-7. doi: 10.1159/000329379.
37. Scherrer U, Rimoldi SF, Rexhaj E, Stuber T, Duplain H, Garcin S, et al. Systemic and pulmonary vascular dysfunction in children conceived by assisted reproductive technologies. Circulation. 2012;125(15):1890-6. doi: 10.1161/CIRCULATIONAHA.111.071183.
38. Meister TA, Rimoldi SF, Soria R, von Arx R, Messerli FH, Sartori C, et al. Association of Assisted Reproductive Technologies With Arterial Hypertension During Adolescence. Journal of the American College of Cardiology. 2018;72(11):1267-74. doi: 10.1016/j.jacc.2018.06.060.

39. de Arriba A, Domínguez M, Labarta JI, Puga B, Mayayo E, Longás AF. Metabolic syndrome and endothelial dysfunction in a population born small for gestational age relationship to growth and Gh therapy. Pediatric endocrinology reviews : PER. 2013;10(3):297-307.
40. Maurice RL, Vaujois L, Dahdah N, Chibab N, Maurice A, Nuyt AM, et al. Carotid wall elastography to assess midterm vascular dysfunction secondary to intrauterine growth restriction: Feasibility and comparison with standardized intima-media thickness. Ultrasound in Medicine and Biology. 2014;40(5):864-70. doi: 10.1016/j.ultrasmedbio.2013.11.013.
41. Xu GF, Zhang JY, Pan HT, Tian S, Liu ME, Yu TT, et al. Cardiovascular dysfunction in offspring of ovarian-hyperstimulated women and effects of estradiol and progesterone: A retrospective cohort study and proteomics analysis. Journal of Clinical Endocrinology and Metabolism. 2014;99(12):E2494-E503. doi: 10.1210/jc.2014-2349.
42. Putra ST, Mansyur M, Sastroasmoro S. Effects of duration of breastfeeding during infancy on vascular dysfunction in adolescents. Acta medica Indonesiana. 2015;47(1):24-30.
43. Sodhi KS, Hondappanavar A, Saxena AK, Dutta S, Khandelwal N. Intima-media complex thickness: Preliminary workup of comparative evaluation of abdominal aorta and carotid artery of small-for-gestation-age term newborns and normal size term newborns. Acta Cardiologica. 2015;70(3):351-7. doi: 10.2143/AC.70.3.3080640.
44. Ciccone MM, Cortese F, Gesualdo M, A DIM, Tafuri S, Mancini G, et al. The role of very low birth weight and prematurity on cardiovascular disease risk and on kidney development in children: a pilot study. Minerva Pediatr. 2016.
45. Faienza MF, Brunetti G, Delvecchio M, Zito A, de Palma FD, Cortese F, et al. Vascular function and myocardial performance indices in children born small for gestational age. Circulation Journal. 2016;80(4):958-63. doi: 10.1253/circj.CJ-15-1038.
46. Olander RFW, Sundholm JKM, Ojala TH, Andersson S, Sarkola T. Neonatal Arterial Morphology Is Related to Body Size in Abnormal Human Fetal Growth. Circulation: Cardiovascular Imaging. 2016;9(9). doi: 10.1161/CIRCIMAGING.116.004657.
47. Dilli D, Ozkan E, Ozkan MB, Aydin B, Özyazici A, Fettah N, et al. Umbilical cord asymmetric dimethylarginine levels and ultrasound assessment of carotid arteries in neonates born small for gestational age. Journal of Maternal-Fetal and Neonatal Medicine. 2017;30(4):492-6. doi: 10.1080/14767058.2016.1176136.
48. Stock K, Schmid A, Griesmaier E, Gande N, Hochmayr C, Knoflach M, et al. The Impact of Being Born Preterm or Small for Gestational Age on Early Vascular Aging in Adolescents. The Journal of pediatrics. 2018;201:49-54.e1. doi: 10.1016/j.jpeds.2018.05.056.
49. Wilde MAD, Eising JB, Gunning MN, Koster MPH, Evelein AMV, Dalmeijer GW, et al. Cardiovascular and Metabolic Health of 74 Children From Women Previously Diagnosed With Polycystic Ovary Syndrome in Comparison With a Population-Based Reference Cohort. Reproductive Sciences. 2018;25(10):1492-500. doi: 10.1177/1933719117749761.
50. Muñiz Fontán M, Oulego Erroz I, Revilla Orias D, Muñoz Lozón A, Rodriguez Núñez A, Lurbe IFE. Thoracic Aortic Intima-Media Thickness in Preschool Children Born Small for Gestational Age. Journal of Pediatrics. 2019. doi: 10.1016/j.jpeds.2018.12.037.
