# Supplementary material for: Risk factors during first 1,000 days of life for carotid intima-media thickness in infants, children, and adolescents: A systematic review with meta-analyses
Source: PLoS Med. 2020 Nov 23;17(11):e1003414. doi: 10.1371/journal.pmed.1003414 (PMC7682901; doi:10.1371/journal.pmed.1003414)
Supplement: S5 Table — (PDF) [file pmed.1003414.s009.pdf]

S5 Table. Association of CIMT with main exposures or interventions types in the first 1000 days of life.

Author, Year	Outcome metric	Exposure time frame	Exposure MM	Level of comparison		Association		
				Exposed	Reference	Measure	Estimates	Covariates*
**CHILD LEVEL: FETAL GROWTH**								
**BIRTH SIZE FOR GESTATIONAL AGE**								
Jouret [1], 2011	CCA-segment N/S-wall (N/S thickness)	Perinatal	not self-reported	small (BW and/or BL < -2 SD)	appropriate (BW and/or BL range N/S)	SMD (95% CI)	0 (-0.369 to 0.369)	N/A
Dratva [2], 2013	CCA-segment far-wall (mean thickness)	Perinatal	not self-reported	small (BW<10th percentile)	appropriate (BW 10th to 90th percentile)	MD (95% CI)	0.002 (-0.009 to 0.014)	child age, sex, BMI, systolic blood pressure, and asthma status at CIMT assessment; delivery mode; maternal cardiovascular and metabolic pregnancy complications; annual household income; and race/ethnicity and study area/community
de Arriba [3], 2013	CCA-segment far-wall (maximum thickness)	Perinatal	N/S	small (BW and/or BL <=-2SD), with or without spontaneous catch-up growth	appropriate (N/S range)	SMD (95% CI)	0.656 (0.394 to 0.917)	N/A
Sodhi [4], 2015	CCA-segment N/S-wall	Perinatal	not self-reported	small (BW < 10th percentile)	appropriate (BW within 50th and 90th percentiles)	SMD (95% CI)	1 (0.584 to 1.416)	N/A

	(mean thickness)							
Sebastiani [5], 2016	CCA-segment far-wall (N/S thickness)	Perinatal	not self-reported	small (BW < -2 SD), with catch-up growth	appropriate (BW within -1SD and +1 SD)	SMD (95% CI)	0.362 (-0.23 to 0.953)	N/A
Faienza [6], 2016	CCA-segment N/S-wall (N/S thickness)	Perinatal	N/S	small (BW and/or BL <3rd percentile)	appropriate (BW and/or BL range N/S)	SMD (95% CI)	1.063 (0.481 to 1.646)	N/A
Stock [7], 2018	CCA-segment far-wall (mean thickness)	Perinatal	not self-reported	small (BW < 10th percentile), with term birth	appropriate (BW range N/S), with term or preterm birth	SMD (95% CI)	-0.085 (-0.3 to 0.13)	N/A
Sebastiani [8], 2019	CCA-segment far-wall (N/S thickness)	Perinatal	not self-reported	small (BW< -2SD), with catch-up growth (weight and height > -2 SD by age 1 year)	appropriate (BW within -1 and +1 SD)	SMD (95% CI)	1.003 (0.438 to 1.568)	N/A
Muñiz Fontán [9], 2019	CCA&ICA &CB-segment far-wall (mean thickness)	Perinatal	unclear	small (BW < 10th percentile)	appropriate (BW within 10th and 90th percentiles)	SMD (95% CI)	0.282 (0.027 to 0.537)	N/A
Crispi [10], 2010	CCA-segment far-wall (mean thickness)	Pregnancy/ Perinatal	not self-reported	small with prenatal signs of FGR (BW<10th percentile + with or without abnormal umbilical artery Doppler)	appropriate (BW>= 10th percentile)	SMD (95% CI)	All: 0.131 (-0.152 to 0.415); Male: 0.481 (0.034 to 0.927); Female: 0.779 (0.394 to 1.163)	N/A

Trevisanuto [11], 2010	CCA-segment far-wall (maximum thickness)	Pregnancy/ Perinatal	N/S	small with prenatal signs of FGR (BW < 10th percentile + prenatal diagnosis of IUGR (criteria N/S))	appropriate (BW range N/S)	SMD (95% CI)	0.299 (-0.346 to 0.944)	N/A
Morsing [12], 2014	CCA-segment N/S-wall (mean thickness)	Pregnancy/ Perinatal	not self-reported	small with prenatal signs of FGR (BW < -2 SD + EFW < -2 SD + absent or reverse end-diastolic blood flow in the umbilical artery), in the context of preterm birth	small with prenatal signs of FGR (BW < -2 SD + EFW < -2 SD + absent or reverse end-diastolic blood flow in the umbilical artery), with term or preterm birth	SMD (95% CI)	-0.47 (-0.907 to -0.034)	N/A
Stergiotou [13], 2014	CCA-segment far-wall (mean thickness)	Pregnancy/ Perinatal	not self-reported	small with prenatal signs of FGR of higher severity (BW and EFW < 3rd percentile or abnormal uterine artery mean pulsatility index or cerebroplacental ratio) or small with prenatal signs of FGR of lower severity (BW and EFW within 3rd and 10th percentile + normal uterine	appropriate (BW and EFW > 10th percentile + no pregnancy complications)	SMD (95% CI)	0.744 (0.442 to 1.046)	N/A

				artery mean pulsatility index or cerebroplacental ratio)				
Maurice [14], 2014	CCA-segment far-wall (N/S thickness)	Pregnancy/ Perinatal	not self-reported	small with prenatal signs of FGR (BW < 3rd percentile + abnormal flow in the uterine and/or umbilical arteries)	appropriate (BW range N/S)	SMD (95% CI)	0 (-1.048 to 1.048)	N/A
Olander [15], 2016	CCA-segment far-wall (mean thickness)	Pregnancy/ Perinatal	not self-reported	small with and without prenatal signs of FGR (BW < -2 SD + with or without (1) umbilical artery resistance index >97.5 percentile, umbilical artery block, or brain sparing on mean cerebral artery Doppler or (2) EFW z-score downshift)	appropriate (BW within -2SD and +2SD)	SMD (95% CI)	-0.333 (-0.711 to 0.045)	N/A
Tzschoppe [16], 2017	CCA-segment N/S-wall (maximum thickness)	Pregnancy/ Perinatal	not self-reported	small with prenatal signs of FGR (BW < 10th percentile + evidence of placental insufficiency: 1) elevated pulsatility index in at least one uterine artery with or without early	appropriate (BW within 25th and 75th percentiles + no evidence of placental insufficiency)	SMD (95% CI)	0.951 (0.017 to 1.885)	N/A

				diastolic notches and/or 2) elevated pulsatility index in the umbilical arteries or absent/reverse diatolic flow)				
Dilli [17], 2017	CCA-segment far-wall (N/S thickness)	Pregnancy/ Perinatal	not self-reported	small with prenatal signs of FGR (BW<10th percentile + abnormal fetal ultrasound biometry and umbilical artery Doppler)	appropriate (BW 10th to 90th percentile)	SMD (95% CI)	1.147 (0.595 to 1.698)	N/A
Atabek [18], 2011	CCA-segment far-wall (maximum thickness)	Perinatal	not self-reported	large (BW > 90th percentile), with exposure to maternal diabetes	appropriate (BW range N/S), with or without exposure to maternal diabetes	SMD (95% CI)	0.496 (-0.093 to 1.086)	N/A
Dratva [2], 2013	CCA-segment far-wall (mean thickness)	Perinatal	not self-reported	large (BW > 90th percentile)	appropriate (BW 10th to 90th percentile)	MD (95% CI)	0.007 (-0.006 to 0.02)	child age, sex, BMI, systolic blood pressure, and asthma status at CIMT assessment; delivery mode; maternal cardiovascular and metabolic pregnancy complications; annual household income; and race/ethnicity and study area/community
Olander [15], 2016	CCA-segment far-	Pregnancy/ Perinatal	not self-reported	large (BW > +2 SD)	appropriate (BW within -2SD and +2SD)	SMD (95% CI)	0.333 (-0.027 to 0.693)	N/A

	wall (mean thickness)							
Evelein [19], 2013	CCA-segment far-wall (N/S thickness)	Perinatal	self-reported	range of values	range of values	Correlation (95% CI)	0.01 (-0.1 to 0.119)	N/A
Rodriguez-Lopez [20], 2016	CCA-segment far-wall (N/S thickness)	Perinatal	not self-reported	range of values	range of values	Correlation (95% CI)	-0.184 (-0.314 to -0.046)	child sex and gestational age at delivery
**BIRTH WEIGHT**								
Gale [21], 2006	CCA-segment far-wall (N/S thickness)	Perinatal	not self-reported	<3.5 kg	>3.5 kg	SMD (95% CI)	-0.156 (-0.464 to 0.152)	child sex
Dratva [2], 2013	CCA-segment far-wall (mean thickness)	Perinatal	not self-reported	<3545 g	>=3545 g	SMD (95% CI)	-0.194 (-0.347 to -0.042)	N/A
Ciccone [22], 2016	CCA-segment far-wall (N/S thickness)	Perinatal	not self-reported	small (very low birth weight, exact criteria and range N/S), with preterm birth	appropriate (criteria & range N/S), with term birth	SMD (95% CI)	-0.083 (-0.802 to 0.636)	N/A
Ayer [23], 2009 (a)	CCA-segment far-wall (mean thickness)	Perinatal	not self-reported	range of values	range of values	Correlation (95% CI)	0.006 (-0.091 to 0.103)	N/A
Atabek [18], 2011	CCA-segment far-wall (maximum thickness)	Perinatal	not self-reported	range of values	range of values	Correlation (95% CI)	0.112 (-0.175 to 0.381)	N/A

Gruszfeld [24], 2015	CCA-segment far-wall (mean thickness)	Perinatal	not self-reported	range of values	range of values	Correlation (95% CI)	All: -0.06 (-0.159 to 0.041); Male: -0.14 (-0.275 to 0.001); Female: 0.02 (-0.124 to 0.163)	N/A
de Arriba [3], 2013	CCA-segment far-wall (maximum thickness)	Perinatal	N/S	range of values	range of values	Correlation (95% CI)	-0.111 (-0.228 to 0.009)	N/A
Sodhi [4], 2015	CCA-segment N/S-wall (mean thickness)	Perinatal	not self-reported	range of values	range of values	Correlation (95% CI)	-0.387 (-0.542 to -0.206)	N/A
Olander [15], 2016	CCA-segment far-wall (mean thickness)	Perinatal	not self-reported	range of values	range of values	Correlation (95% CI)	0.247 (0.102 to 0.382)	N/A
Dilli [17], 2017	CCA-segment far-wall (N/S thickness)	Perinatal	not self-reported	range of values	range of values	Correlation (95% CI)	-0.22 (-0.449 to 0.036)	N/A
**BIRTH LENGTH**								
Atabek [18], 2011	CCA-segment far-wall (maximum thickness)	Perinatal	not self-reported	range of values	range of values	Correlation (95% CI)	0.084 (-0.202 to 0.356)	N/A
Skilton [25], 2013	CCA-segment far-wall	Perinatal	not self-reported	range of values	range of values	Correlation (95% CI)	0 (-0.105 to 0.105)	child age, gender, randomization to intervention, gestational age, length-adjusted

	(maximum thickness)							birth weight, breastfeeding at 6 months, change in weight-for-height Z-score between 0 and 18 months of age; paternal BMI; maternal education
de Arriba [3], 2013	CCA-segment far-wall (maximum thickness)	Perinatal	N/S	range of values	range of values	Correlation (95% CI)	-0.211 (-0.323 to -0.093)	N/A
Sodhi [4], 2015	CCA-segment N/S-wall (mean thickness)	Perinatal	not self-reported	range of values	range of values	Correlation (95% CI)	-0.326 (-0.491 to -0.138)	N/A
**BIRTH HEAD CIRCUMFERENCE**								
Atabek [18], 2011	CCA-segment far-wall (maximum thickness)	Perinatal	not self-reported	range of values	range of values	Correlation (95% CI)	0.084 (-0.186 to 0.341)	N/A
Olander [15], 2016	CCA-segment far-wall (mean thickness)	Perinatal	not self-reported	range of values	range of values	Correlation (95% CI)	0.299 (0.157 to 0.429)	N/A
Sodhi [4], 2015	CCA-segment N/S-wall (mean thickness)	Perinatal	not self-reported	range of values	range of values	Correlation (95% CI)	-0.349 (-0.510 to -0.164)	N/A
**BIRTH PONDERAL INDEX**								
Atabek [18], 2011	CCA-segment far-	Perinatal	not self-reported	range of values	range of values	Correlation (p-value)	Appropriate size at birth & not	N/A

	wall (maximum thickness)						exposed to gestational diabetes: -0.045 (0.830); Appropriate size at birth & exposed to gestational diabetes: 0.437 (0.118); Large size at birth & exposed to gestational diabetes: -0.069 (0.798)	
Olander [15], 2016	CCA-segment far-wall (mean thickness)	Perinatal	not self-reported	range of values (100Xg/cm^3)	range of values (100Xg/cm^3)	b (p-value)	0.016 (0.002)	N/A
**BIRTH BODY SURFACE AREA**								
Stergiotou [13], 2014	CCA-segment far-wall (mean thickness)	Perinatal	N/S	range of values (m^2)	range of values (m^2)	b (95% CI)	-0.386 (-0.511 to -0.261)	unclear (potentially: child gender, age at evaluation, gestational age at delivery and size for gestation group)
Olander [15], 2016	CCA-segment far-wall (mean thickness)	Perinatal	not self-reported	range of values (N/S unit)	range of values (N/S unit)	b (p-value)	0.260 (< 0.001)	N/A
**CHILD LEVEL: GESTATIONAL AGE**								
**GESTATIONAL AGE**								
Dratva [2], 2013	CCA-segment far-	Perinatal	not self-reported	postterm (>41 weeks)	term (37 to 41 weeks)	SMD (95% CI)	0.479 (0.267 to 0.69)	N/A

	wall (mean thickness)							
Crispi [10], 2010	CCA-segment far-wall (mean thickness)	Perinatal	not self-reported	preterm (range N/S)	term (range N/S)	SMD (95% CI)	0.238 (-0.143 to 0.618)	N/A
Dratva [2], 2013	CCA-segment far-wall (mean thickness)	Perinatal	not self-reported	preterm (<37 weeks)	term (37 to 41 weeks)	SMD (95% CI)	0.35 (0.007 to 0.693)	N/A
Schubert [26], 2013	CCA-segment far-wall (mean thickness)	Perinatal	not self-reported	preterm (26 to 30 weeks)	term (range N/S)	SMD (95% CI)	0.027 (-0.534 to 0.589)	N/A
Lee [27], 2014	CCA-segment far-wall (mean thickness)	Perinatal	not self-reported	preterm (range N/S)	age and sex-specific normative values	MD (95% CI)	0.07 (not available)	N/A
Morsing [12], 2014	CCA-segment N/S-wall (mean thickness)	Perinatal	not self-reported	preterm (< 30 weeks)	term (range N/S)	SMD (95% CI)	-0.374 (-0.805 to 0.058)	N/A
Mohlkert [28], 2017	CCA-segment far-wall (mean thickness)	Perinatal	not self-reported	preterm (22 to 26 weeks)	term (37 to 41 weeks)	SMD (95% CI)	0.125 (-0.132 to 0.381)	N/A
Ciccone [22], 2016	CCA-segment far-wall (N/S thickness)	Perinatal	not self-reported	preterm (range N/S)	term (range N/S)	SMD (95% CI)	-0.083 (-0.802 to 0.636)	N/A
Stock [7], 2018	CCA-segment far-	Perinatal	not self-reported	preterm (<37 weeks)	term (>37 weeks)	SMD (95% CI)	-0.188 (-0.414 to 0.037)	N/A

	wall (mean thickness)							
Skilton [29], 2012	CCA-segment far-wall (mean thickness)	Perinatal	not self-reported	range of values	range of values	Correlation (95% CI)	0.073 (-0.032 to 0.175)	child sex and birth weight
Rodriguez-Lopez [20], 2016	CCA-segment far-wall (N/S thickness)	Perinatal	not self-reported	range of values	range of values	Correlation (95% CI)	-0.295 (-0.417 to -0.164)	child sex
Olander [15], 2016	CCA-segment far-wall (mean thickness)	Perinatal	not self-reported	range of values	range of values	Correlation (95% CI)	0.212 (0.065 to 0.35)	N/A
**CHILD LEVEL: POSTNATAL DIET AND FEEDING PRATICES**								
**BREASTFEEDING DURATION**								
Gale [21], 2006	CCA-segment far-wall (N/S thickness)	Postnatal	N/S	> 0 months BF	never BF	SMD (95% CI)	-0.1 (-0.391 to 0.191)	child sex
Skilton [25], 2013	CCA-segment far-wall (maximum thickness)	Postnatal	self-reported	ongoing BF at age 6 months	discontinued BF before age 6 months	SMD (95% CI)	-0.125 (-0.325 to 0.075)	child age, gender, randomization to intervention, gestational age, birth length, length-adjusted birth weight, change in weight-for-height Z-score between 0 and 18 months of age; paternal BMI; maternal education
Putra [30], 2015	N/S-segment N/S-wall (N/S thickness)	Postnatal	self-reported	≥ 2 months BF	never or < 2 months BF	SMD (95% CI)	-0.117 (-0.513 to 0.28)	N/A

Rodriguez-Lopez [20], 2016	CCA-segment far-wall (N/S thickness)	Postnatal	self-reported	≥ 1-month BF	< 1-month BF	SMD (95% CI)	-0.162 (-0.55 to 0.226)	child sex and gestational age at delivery
Evelein [31], 2011	CCA-segment far-wall (N/S thickness)	Postnatal	self-reported	> 0 months exclusive BF	never BF	SMD (95% CI)	0.217 (-0.046 to 0.479)	N/A
Gruszfeld [24], 2015	CCA-segment far-wall (mean thickness)	Postnatal	self-reported	3 months exclusive BF	never or < 2 months BF	SMD (95% CI)	All: 0.129 (-0.085 to 0.343); Male: 0.127 (-0.175 to 0.429); Female: 0.142 (-0.161 to 0.445)	N/A
**POLYUNSATURATED FATTY ACIDS INTAKE**								
Ayer [32], 2009 (b)	CCA-segment far-wall (mean thickness)	Postnatal	Intervention (RCT; administration by parents)	higher (n-3 to n-6 fatty acids ratio of about 1:5; participants were provided with 1) canola-based oils and spreads (16% n-6 and 6% n-3 fatty acids) to use from the time of enrollment; 2) tuna oil (500-mg capsule containing 37% n-3 and 6% n-6), once daily, from the start of bottle feeding if <6 months of age or from 6 months of age; the dose of	lower (n-3 to n-6 fatty acids ratio in the range of 1:15-1:20; participants were provided with 1) polyunsaturated oils and spreads (40% n-6 fatty acids) to use from enrollment; 2) sunola oils (500-mg capsule containing 0.3% n-3 and 7% n-6	SMD (95% CI)	0 (-0.195 to 0.195)	N/A

				tuna oil was 12-18 drops from a 500-mg capsule if < 6 months of age or the entire content of a capsule starting 6 months of age.)	fatty acids) once daily from the start of bottle feeding or to solid foods introduction at 6 months of age, whichever came first; the dosage of sunola oil was similar to the protocol used for tuna oil.)			
Pluymen [33], 2017	CCA-segment far-wall (maximum thickness)	Postnatal	self-reported (cohort study)	higher (participants used formula milk supplemented with long chain polyunsaturated fatty acids)	lower (participants used formula milk non-supplemented with long chain polyunsaturated fatty acids)	SMD (95% CI)	0.532 (0.197 to 0.868)	N/A
**PROTEIN INTAKE**								
Gruszfeld [24], 2015	CCA-segment far-wall (mean thickness)	Postnatal	Interventio n (double-blind RCT)	higher (cow-milk formula with 2.05 and 3.2 g/dl protein content was given during the first year of life to infants whose parents opted for exclusive formula-feeding shortly after birth)	lower (cow-milk formula with 1.25 and 1.6 g/dl protein content was given during the first year of life to infants whose parents opted for exclusive formula-feeding	SMD (95% CI)	-0.205 (-0.45 to 0.04)	N/A

					shortly after birth)			
**CHILD LEVEL: POSTNATAL GROWTH**								
**WEIGHT GAIN as weight change rate in g/day between 0 and 3 months**								
Evelein [19], 2013	CCA-segment far-wall (N/S thickness)	Postnatal	self-reported	range of values (g/day)	range of values (g/day)	b (95%CI)	0.002 (0.001 to 0.003)	N/A
**WEIGHT GAIN as weight-for-length Z-score change rate between 0 and 3 months**								
Evelein [19], 2013	CCA-segment far-wall (N/S thickness)	Postnatal	self-reported	range of values	range of values	b (95%CI)	0.006 (0.002 to 0.010)	N/A
**WEIGHT GAIN as height-adjusted weight change in kg between 0 and 18 months**								
Skilton [25], 2013	CCA-segment far-wall (maximum thickness)	Postnatal	not self-reported	range of values	range of values	Correlation (p-value)	0.171 (<0.001)	N/A
**WEIGHT GAIN as weight change in kg between 0 and 18 months**								
Skilton [25], 2013	CCA-segment far-wall (maximum thickness)	Postnatal	not self-reported	range of values	range of values	Correlation (p-value)	0.147 (0.003)	N/A
**WEIGHT GAIN as weight-for-height Z-score change between 0 and 18 months**								
Skilton [25], 2013	CCA-segment far-wall (maximum thickness)	Postnatal	not self-reported	range of values	range of values	Correlation (p-value)	0.143 (0.005)	N/A

**WEIGHT GAIN as weight Z-score change > 0.67 between 0 and 4 to 5 years**								
Rodriguez-Lopez [20], 2016	CCA-segment far-wall (N/S thickness)	Postnatal	not self-reported	yes	no	MD (95%CI)	0.010 (0.000 to 0.021)	child sex and gestational age
**WEIGHT as absolute value at 9 months**								
Gale [21], 2006	CCA-segment far-wall (N/S thickness)	Postnatal	not self-reported	Quarter 1: < 8.3 kg; Quarter 2: < 9 kg; Quarter 3: < 9.7 kg	Quarter 4: > 9.7 kg	MD (95%CI)	-0.021 (-0.045 to 0.003); -0.026 (-0.050 to -0.001); -0.019 (-0.043 to 0.005)	child sex
**LENGTH GAIN as length change rate in mm/day between 0 and 3 months**								
Evelein [19], 2013	CCA-segment far-wall (N/S thickness)	Postnatal	self-reported	range of values (mm/day)	range of values (mm/day)	b (95%CI)	-0.028 (-0.115 to 0.060)	N/A
**FAMILY LEVEL: CONCEPTION, DELIVERY, AND PREGNANCY COMPLICATIONS**								
**MODE OF CONCEPTION**								
Scherrer [34], 2012	CCA-segment N/S-wall (mean thickness)	Periconceptional	N/S	ART conception (in vitro fertilization or intracytoplasmic sperm injection)	Natural conception	SMD (95% CI)	1.545 (0.824 to 2.266)	N/A
Xu [35], 2014	CCA-segment N/S-wall (N/S thickness)	Periconceptional	self-reported	ART conception (in vitro fertilization, with or without ovarian hyperstimulation syndrome)	Natural conception	SMD (95% CI)	-0.167 (-0.529 to 0.195)	N/A

Valenzuela-Alcaraz [36], 2019	CCA-segment far-wall (mean thickness)	Periconceptional	self-reported	ART conception (standard in vitro fertilization or intracytoplasmic sperm injection)	Natural conception	SMD (95% CI)	1.043 (0.713 to 1.374)	N/A
**MATERNAL AGE AT CONCEPTION**								
Scherrer [34], 2012	CCA-segment N/S-wall (mean thickness)	Periconceptional	N/S	range of values	range of values	Correlation (p-value)	0.170 (0.300)	N/A
**PATERNAL AGE AT CONCEPTION**								
Rodriguez-Lopez [20], 2016	CCA-segment far-wall (N/S thickness)	Periconceptional	self-reported	range of values (year)	range of values (year)	b (p-value)	0.001 (0.070)	child sex and gestational age
**EXPOSURE TO MATERNAL DIABETES**								
Ayer [23], 2009 (a)	CCA-segment far-wall (mean thickness)	Pregnancy	self-reported	yes (gestational diabetes)	no	SMD (95% CI)	0 (-0.412 to 0.412)	N/A
Sundholm [37], 2019	CCA-segment far-wall (N/S thickness)	Pregnancy	not self-reported	yes (gestational diabetes)	no	SMD (95% CI)	0 (-0.279 to 0.279)	N/A
Atabek [18], 2011	CCA-segment far-wall (maximum thickness)	Pregnancy	N/S	yes (type N/S)	no	SMD (95% CI)	0.464 (-0.074 to 1.002)	N/A

**EXPOSURE TO PREECLAMPSIA OR GESTATIONAL HYPERTENSION**								
Ayer [23], 2009 (a)	CCA-segment far-wall (mean thickness)	Pregnancy	self-reported	yes	no	MD (p-value)	0.000 (0.880)	N/A
Rodriguez-Lopez [20], 2016	CCA-segment far-wall (N/S thickness)	Pregnancy	not self-reported	yes	no	MD (95%CI)	0.009 (-0.005 to 0.023)	child sex and gestational age
**EXPOSURE TO PREGNANCY COMPLICATIONS**								
Dratva [2], 2013	CCA-segment far-wall (mean thickness)	Pregnancy	not self-reported	yes (eclampsia, pre-eclampsia, gestational diabetes, gestational hypertension, cardiac disease)	no	MD (p-value)	0.012 (0.164)	N/A
**FAMILY LEVEL: MATERNAL OR PATERNAL GROWTH**								
**MATERNAL PRE-PREGNANCY BODY MASS INDEX**								
Gale [21], 2006	CCA-segment far-wall (N/S thickness)	Periconceptional	self-reported	< 20 kg/m^2;<br><22 kg/m^2;<br><24 kg/m^2;	> 24 kg/m^2	MD (95%CI)	-0.018 (-0.042 to 0.006);<br>0.009 (-0.013 to 0.032);<br>-0.007 (-0.032 to 0.017)	child sex
Sundholm [37], 2019	CCA-segment far-wall (N/S thickness)	Periconceptional	self-reported (for some subjects); mixed (for other subjects)	range of values	range of values	Correlation (p-value)	0.050 (> 0.05)	N/A

Gruszfeld [24], 2015	CCA-segment far-wall (mean thickness)	Periconceptional	mixed	range of values	range of values	Correlation (p-value)	All: 0.000 (> 0.05)<br>Male: 0.030 (> 0.05)<br>Female: -0.040 (> 0.05)	N/A
**MATERNAL WEIGHT GAIN as weight change between delivery and pre-pregnancy according to pre-pregnancy body mass index status**								
Carreras-Badosa [38], 2018	CCA-segment N/S-wall (N/S thickness)	Pregnancy	not self-reported	insufficient; excessive	adequate	mean (SD) for each category<br><br>[trend p-value]	insufficient: 0.370 (0.030);<br>adequate: 0.370 (0.030);<br>excessive: 0.370 (0.030)<br><br>[0.680]	N/A
**PATERNAL BODY MASS INDEX**								
Gruszfeld [24], 2015	CCA-segment far-wall (mean thickness)	Perinatal	not self-reported	range of values	range of values	Correlation (p-value)	All: -0.080 (> 0.05)<br>Male: -0.120 (> 0.05)<br>Female: -0.040 (> 0.05)	N/A
**MATERNAL SERUM TOTAL 25 (OH)- VITAMIN D**								
Gale [39], 2008	CCA-segment far-wall (N/S thickness)	Pregnancy	not self-reported	Quarter 1 (<30 nmol/L);<br>Quarter 2 (30-50 nmol/L);<br>Quarter 3 (51-75 nmol/L)	Quarter 4 (>75 nmol/L)	mean (SD) for each category<br><br>[p-value from linear regression]	Quarter 1: 0.330 (0.050);<br>Quarter 2: 0.360 (0.070);<br>Quarter 3: 0.340 (0.060);<br>Quarter 4: 0.340 (0.070)<br><br>[0.660]	N/A

Carreras-Badosa [38], 2018	CCA-segment N/S-wall (N/S thickness)	Pregnancy	not self-reported	range of values	range of values	Correlation (p-value)	-0.248(0.010)	N/A
**ENVIRONMENTA LEVEL: TOBACCO EXPOSURE**								
**EXPOSURE TO MATERNAL SMOKING**								
Gale [21], 2006	CCA-segment far-wall (N/S thickness)	Pregnancy	self-reported	yes	no	SMD (95% CI)	0.050 (-0.304 to 0.404)	child sex
Ayer [40], 2011	CCA-segment far-wall (mean thickness)	Pregnancy	self-reported	yes	no	SMD (95% CI)	0.078 (-0.153 to 0.310)	N/A
Geerts [41], 2012	CCA-segment far-wall (N/S thickness)	Pregnancy	self-reported	yes	no	SMD (95% CI)	0.466 (-0.057 to 0.989)	N/A
**NUMBER OF CIGARETTES PER DAY SMOKED BY THE MOTHER**								
Gruszfeld [24], 2015	CCA-segment far-wall (mean thickness)	Pregnancy	self-reported	range of values (number of cigarettes/day)	range of values (number of cigarettes/day)	Correlation (95% CI)	All: -0.050 (-0.150 to 0.051); Male: -0.070 (-0.209 to 0.072); Female: -0.030 (-0.173 to 0.114)	N/A
**EXPOSURE TO PATERNAL SMOKING**								
Rodriguez-Lopez [20], 2016	CCA-segment far-wall (N/S thickness)	Pregnancy	self-reported	yes	no	MD (95% CI)	0.007 (-0.002 to 0.016)	child sex and gestational age at delivery

**EXPOSURE TO HOUSEHOLD SMOKING**								
Ayer [40], 2011	CCA-segment far-wall (mean thickness)	Postnatal	self-reported	yes	no	MD (95% CI)	0 (-0.009 to 0.020)	N/A
**ENVIRONMENTA LEVEL: SOCIOECONOMIC STATUS**								
**SOCIOECONOMIC STATUS based on maternal occupation**								
Gale [21], 2006	CCA-segment far-wall (N/S thickness)	Pregnancy	self-reported	medium (skilled non-manual and manual); high (professional/ intermediate)	low (partly skilled/ unskilled)	MD (95% CI) [trend p-value]	-0.010 (-0.016 to 0.035); -0.030 (-0.023 to 0.016) ‡ [0.589]	child sex
Rodriguez-Lopez [20], 2016	CCA-segment far-wall (N/S thickness)	Perinatal	self-reported	low (routine occupation, long-term unemployment, or never worked)	high (otherwise)	MD (95% CI)	0.014 (0.001 to 0.027)	child sex and gestational age
**SOCIOECONOMIC STATUS based on maternal education**								
Skilton [25], 2013	CCA-segment far-wall (maximum thickness)	Pregnancy	self-reported	medium (11-12 years of school); high (tertiary education)	low (≤ 10 years of school)	MD (95% CI)	-0.010 (-0.030 to 0.020); -0.020 (-0.040 to 0.000)	child age, sex, randomization to intervention, gestational age, birth length, length-adjusted birth weight, breastfeeding at 6 months, change in weight-for-height Z-score between 0 and 18 months of age; paternal BMI
**SOCIOECONOMIC STATUS based on combined household income, parental occupation, parental education**								
Liu [42], 2017	CCA-segment far-wall (maximum thickness)	Postnatal	self-reported	low-medium (second quartile); medium-high (third quartile);	low (lowest quartile)	mean (95%CI) in each category	low: 0.584 (0.579 to 0.589) †; low-medium: 0.582 (0.577 to 0.587) †;	child age, sex, body mass index Z-score, mean arterial blood pressure, pubertal category, minimal intima-media lumen

				high (highest quartile)		[trend p-value]	medium-high: 0.580 (0.575 to 0.585)†; high: 0.576 (0.571 to 0.581)† [0.06]	diameter, and exposure to passive smoking
**SOCIOECONOMIC STATUS based on SEIFA INDEX OF RELATIVE SOCIOECONOMIC ADVANTAGE and DISADVANTAGE SCORE- according to child's postcode of residence**								
Liu [42], 2017	CCA-segment far-wall (maximum thickness)	Postnatal	not self-reported	low-medium (second quartile); medium-high (third quartile); high (highest quartile)	low (lowest quartile)	mean (95%CI) in each category [trend p-value]	low: 0.582 (0.576 to 0.587)†; low-medium: 0.581 (0.576 to 0.585)†; medium-high: 0.583 (0.578 to 0.589)†; high: 0.577 (0.573 to 0.582)†; [0.13]	child age, sex, body mass index Z-score, mean arterial blood pressure, pubertal category, minimal intima-media lumen diameter, and exposure to passive smoking

Note: b represents the difference in CIMT in millimeters per 1-unit difference in exposure; MD represents the difference in CIMT between exposed and reference in millimeters; mean represents the CIMT summary value in mm; SMD represents the difference in CIMT between exposed and reference in units of standard deviation.

* Covariates from partial associations.

† Data reported only graphically and extracted with the software WebPlot Digitizer- Version 4.1 (https://automeris.io/WebPlotDigitizer).

‡ Data was not reliably reported in the original report as the point estimate is not comprised within the CI limits.

Abbreviations: ART, assisted reproductive technologies; b, unstandardized regression coefficient; BF, breastfeeding; BL, birth length; BW, birth weight; CCA, common carotid artery; CI, confidence interval; cm, centimeter; dl, deciliter; EFW, estimated fetal weight; FGR, fetal growth restriction; g, gram; kg, kilogram; m, meter; MM, measurement method; MD, mean difference; SD, standard deviation; SMD, standardized mean difference; RCT, randomized controlled trial.

References

1. Jouret B, Dulac Y, Bassil Eter R, Taktak A, Cristini C, Lounis N, et al. Endothelial function and mechanical arterial properties in children born small for gestational age: comparison with obese children. Hormone research in paediatrics. 2011;76(4):240-7. doi: 10.1159/000329379.
2. Dratva J, Breton CV, Hodis HN, Mac KWJ, Salam MT, Zemp E, et al. Birth weight and carotid artery intima-media thickness. Journal of Pediatrics. 2013;162(5):906-11.e2. doi: 10.1016/j.jpeds.2012.10.060.
3. de Arriba A, Domínguez M, Labarta JI, Puga B, Mayayo E, Longás AF. Metabolic syndrome and endothelial dysfunction in a population born small for gestational age relationship to growth and Gh therapy. Pediatric endocrinology reviews : PER. 2013;10(3):297-307.
4. Sodhi KS, Hondappanavar A, Saxena AK, Dutta S, Khandelwal N. Intima-media complex thickness: Preliminary workup of comparative evaluation of abdominal aorta and carotid artery of small-for-gestation-age term newborns and normal size term newborns. Acta Cardiologica. 2015;70(3):351-7. doi: 10.2143/AC.70.3.3080640.
5. Sebastiani G, Díaz M, Bassols J, Aragonés G, López-Bermejo A, de Zegher F, et al. The sequence of prenatal growth restraint and post-natal catch-up growth leads to a thicker intima-media and more pre-peritoneal and hepatic fat by age 3–6 years. Pediatric Obesity. 2016;11(4):251-7. doi: 10.1111/ijpo.12053.
6. Faienza MF, Brunetti G, Delvecchio M, Zito A, de Palma FD, Cortese F, et al. Vascular function and myocardial performance indices in children born small for gestational age. Circulation Journal. 2016;80(4):958-63. doi: 10.1253/circj.CJ-15-1038.
7. Stock K, Schmid A, Griesmaier E, Gande N, Hochmayr C, Knoflach M, et al. The Impact of Being Born Preterm or Small for Gestational Age on Early Vascular Aging in Adolescents. The Journal of pediatrics. 2018;201:49-54.e1. doi: 10.1016/j.jpeds.2018.05.056.
8. Sebastiani G, García-Beltran C, Pie S, Guerra A, López-Bermejo A, de Toledo JS, et al. The sequence of prenatal growth restraint and postnatal catch-up growth: normal heart but thicker intima-media and more pre-peritoneal fat in late infancy. Pediatric Obesity. 2019;14(3). doi: 10.1111/ijpo.12476.
9. Muñiz Fontán M, Oulego Erroz I, Revilla Orias D, Muñoz Lozón A, Rodriguez Núñez A, Lurbe IFE. Thoracic Aortic Intima-Media Thickness in Preschool Children Born Small for Gestational Age. Journal of Pediatrics. 2019. doi: 10.1016/j.jpeds.2018.12.037.
10. Crispi F, Bijnens B, Figueras F, Bartrons J, Eixarch E, Le Noble F, et al. Fetal growth restriction results in remodeled and less efficient hearts in children. Circulation. 2010;121(22):2427-36. doi: 10.1161/circulationaha.110.937995.
11. Trevisanuto D, Avezzú F, Cavallin F, Doglioni N, Marzolo M, Verlato F, et al. Arterial wall thickness and blood pressure in children who were born small for gestational age: Correlation with umbilical cord high-sensitivity C-reactive protein. Archives of disease in childhood. 2010;95(1):31-4. doi: 10.1136/adc.2008.150326.
12. Morsing E, Liuba P, Fellman V, Maršál K, Brodszki J. Cardiovascular function in children born very preterm after intrauterine growth restriction with severely abnormal umbilical artery blood flow. European journal of preventive cardiology. 2014;21(10):1257-66. doi: 10.1177/2047487313486044.

13. Stergiotou I, Crispi F, Valenzuela-Alcaraz B, Cruz-Lemini M, Bijnens B, Gratacos E. Aortic and carotid intima-media thickness in term small-for-gestational-age newborns and relationship with prenatal signs of severity. Ultrasound in obstetrics & gynecology : the official journal of the International Society of Ultrasound in Obstetrics and Gynecology. 2014;43(6):625-31. doi: 10.1002/uog.13245.
14. Maurice RL, Vaujois L, Dahdah N, Chibab N, Maurice A, Nuyt AM, et al. Carotid wall elastography to assess midterm vascular dysfunction secondary to intrauterine growth restriction: Feasibility and comparison with standardized intima-media thickness. Ultrasound in Medicine and Biology. 2014;40(5):864-70. doi: 10.1016/j.ultrasmedbio.2013.11.013.
15. Olander RFW, Sundholm JKM, Ojala TH, Andersson S, Sarkola T. Neonatal Arterial Morphology Is Related to Body Size in Abnormal Human Fetal Growth. Circulation: Cardiovascular Imaging. 2016;9(9). doi: 10.1161/CIRCIMAGING.116.004657.
16. Tzschoppe A, Von Kries R, Struwe E, Rascher W, Dörr HG, Jüngert J, et al. Intrauterine Growth Restriction (IUGR) Induces Signs of Subclinical Atherosclerosis in 6-Year-Old Infants Despite Absence of Excessive Growth. Klinische Padiatrie. 2017;229(4):209-15. doi: 10.1055/s-0043-104528.
17. Dilli D, Ozkan E, Ozkan MB, Aydin B, Özyazici A, Fettah N, et al. Umbilical cord asymmetric dimethylarginine levels and ultrasound assessment of carotid arteries in neonates born small for gestational age. Journal of Maternal-Fetal and Neonatal Medicine. 2017;30(4):492-6. doi: 10.1080/14767058.2016.1176136.
18. Atabek ME, Çağan HH, Eklioğlu BS, Oran B. Absence of increase in carotid artery Intima-Media thickness in infants of diabetic mothers. JCRPE Journal of Clinical Research in Pediatric Endocrinology. 2011;3(3):144-8. doi: 10.4274/jcrpe.v3i3.28.
19. Evelein AMV, Visseren FLJ, Van Der Ent CK, Grobbee DE, Uiterwaal CSPM. Excess early postnatal weight gain leads to thicker and stiffer arteries in young children. Journal of Clinical Endocrinology and Metabolism. 2013;98(2):794-801. doi: 10.1210/jc.2012-3208.
20. Rodriguez-Lopez M, Osorio L, Acosta-Rojas R, Figueras J, Cruz-Lemini M, Figueras F, et al. Influence of breastfeeding and postnatal nutrition on cardiovascular remodeling induced by fetal growth restriction. Pediatric research. 2016;79(1):100-6. doi: 10.1038/pr.2015.182.
21. Gale CR, Jiang B, Robinson SM, Godfrey KM, Law CM, Martyn CN. Maternal diet during pregnancy and carotid intima-media thickness in children. Arteriosclerosis, thrombosis, and vascular biology. 2006;26(8):1877-82. doi: 10.1161/01.ATV.0000228819.13039.b8.
22. Ciccone MM, Cortese F, Gesualdo M, A DIM, Tafuri S, Mancini G, et al. The role of very low birth weight and prematurity on cardiovascular disease risk and on kidney development in children: a pilot study. Minerva Pediatr. 2016.
23. Ayer JG, Harmer JA, Nakhla S, Xuan W, Ng MKC, Raitakari OT, et al. HDL-cholesterol, blood pressure, and asymmetric dimethylarginine are significantly associated with arterial wall thickness in children. Arteriosclerosis, thrombosis, and vascular biology. 2009;29(6):943-9. doi: 10.1161/ATVBAHA.109.184184.
24. Gruszfeld D, Weber M, Nowakowska-Rysz M, Janas R, Kozlik-Feldmann R, Xhonneux A, et al. Protein intake in infancy and carotid intima media thickness at 5 years - A secondary analysis from a randomized trial for the European childhood obesity study group. Annals of Nutrition and Metabolism. 2015;66(1):51-9. doi: 10.1159/000369980.
25. Skilton MR, Marks GB, Ayer JG, Garden FL, Garnett SP, Harmer JA, et al. Weight gain in infancy and vascular risk factors in later childhood. Pediatrics. 2013;131(6):e1821-e8. doi: 10.1542/peds.2012-2789.

26. Schubert U, Müller M, Abdul-Khaliq H, Norman M, Bonamy AKE. Relative intima-media thickening after preterm birth. Acta Paediatrica, International Journal of Paediatrics. 2013;102(10):965-9. doi: 10.1111/apa.12355.
27. Lee H, Dichtl S, Mormanova Z, Dalla Pozza R, Genzel-Boroviczeny O. In adolescence, extreme prematurity is associated with significant changes in the microvasculature, elevated blood pressure and increased carotid intima - Media thickness. Archives of Disease in Childhood: Education and Practice Edition. 2014;99(10):907-11. doi: 10.1136/archdischild-2013-304074.
28. Mohlkert LA, Hallberg J, Broberg O, Hellström M, Pegelow Halvorsen C, Sjöberg G, et al. Preterm arteries in childhood: Dimensions, intima-media thickness, and elasticity of the aorta, coronaries, and carotids in 6-y-old children born extremely preterm. Pediatric research. 2017;81(2):299-306. doi: 10.1038/pr.2016.212.
29. Skilton MR, Ayer JG, Harmer JA, Webb K, Leeder SR, Marks GB, et al. Impaired fetal growth and arterial wall thickening: A randomized trial of omega-3 supplementation. Pediatrics. 2012;129(3):e698-e703. doi: 10.1542/peds.2011-2472.
30. Putra ST, Mansyur M, Sastroasmoro S. Effects of duration of breastfeeding during infancy on vascular dysfunction in adolescents. Acta medica Indonesiana. 2015;47(1):24-30.
31. Evelein AMV, Geerts CC, Visseren FLJ, Bots ML, Van Der Ent CK, Grobbee DE, et al. The association between breastfeeding and the cardiovascular system in early childhood. American Journal of Clinical Nutrition. 2011;93(4):712-8. doi: 10.3945/ajcn.110.002980.
32. Ayer JG, Harmer JA, Xuan W, Toelle B, Webb K, Almqvist C, et al. Dietary supplementation with n-3 polyunsaturated fatty acids in early childhood: Effects on blood pressure and arterial structure and function at age 8 y. American Journal of Clinical Nutrition. 2009;90(2):438-46. doi: 10.3945/ajcn.2009.27811.
33. Pluymen LPM, Dalmeijer GW, Smit HA, Uiterwaal C, van der Ent CK, van Rossem L. Long-chain polyunsaturated fatty acids in infant formula and cardiovascular markers in childhood. Matern Child Nutr. 2017;14(2):e12523. doi: 10.1111/mcn.12523.
34. Scherrer U, Rimoldi SF, Rexhaj E, Stuber T, Duplain H, Garcin S, et al. Systemic and pulmonary vascular dysfunction in children conceived by assisted reproductive technologies. Circulation. 2012;125(15):1890-6. doi: 10.1161/CIRCULATIONAHA.111.071183.
35. Xu GF, Zhang JY, Pan HT, Tian S, Liu ME, Yu TT, et al. Cardiovascular dysfunction in offspring of ovarian-hyperstimulated women and effects of estradiol and progesterone: A retrospective cohort study and proteomics analysis. Journal of Clinical Endocrinology and Metabolism. 2014;99(12):E2494-E503. doi: 10.1210/jc.2014-2349.
36. Valenzuela-Alcaraz B, Serafini A, Sepulveda-Martinez A, Casals G, Rodriguez-Lopez M, Garcia-Otero L, et al. Postnatal persistence of fetal cardiovascular remodelling associated with assisted reproductive technologies: a cohort study. Bjog. 2019;126(2):291-8. doi: 10.1111/1471-0528.15246.
37. Sundholm JKM, Litwin L, Rönö K, Koivusalo SB, Eriksson JG, Sarkola T. Maternal obesity and gestational diabetes: Impact on arterial wall layer thickness and stiffness in early childhood - RADIEL study six-year follow-up. Atherosclerosis. 2019. doi: 10.1016/j.atherosclerosis.2019.01.037.

38. Carreras-Badosa G, Armero-Bujaldón C, Solé-Amat L, Prats-Puig A, Díaz-Roldán F, Soriano-Rodriguez P, et al. Serum 25-hydroxyvitamin D and cardiovascular disease risk factors in women with excessive weight gain during pregnancy and in their offspring at age 5-6 years. International Journal of Obesity. 2018;42(5):1019-28. doi: 10.1038/s41366-018-0101-6.
39. Gale CR, Robinson SM, Harvey NC, Javaid MK, Jiang B, Martyn CN, et al. Maternal vitamin D status during pregnancy and child outcomes. European Journal of Clinical Nutrition. 2008;62(1):68-77. doi: 10.1038/sj.ejcn.1602680.
40. Ayer JG, Belousova E, Harmer JA, David C, Marks GB, Celermajer DS. Maternal cigarette smoking is associated with reduced high-density lipoprotein cholesterol in healthy 8-year-old children. European heart journal. 2011;32(19):2446-53. doi: 10.1093/eurheartj/ehr174.
41. Geerts CC, Bots ML, Van Der Ent CK, Grobbee DE, Uiterwaal CSPM. Parental smoking and vascular damage in their 5-year-old children. Pediatrics. 2012;129(1):45-54. doi: 10.1542/peds.2011-0249.
42. Liu RS, Mensah FK, Carlin J, Edwards B, Ranganathan S, Cheung M, et al. Socioeconomic Position Is Associated With Carotid Intima-Media Thickness in Mid-Childhood: The Longitudinal Study of Australian Children. Journal of the American Heart Association. 2017;6(8). doi: 10.1161/jaha.117.005925.
