# Supplementary material for: Risk factors during first 1,000 days of life for carotid intima-media thickness in infants, children, and adolescents: A systematic review with meta-analyses
Source: PLoS Med. 2020 Nov 23;17(11):e1003414. doi: 10.1371/journal.pmed.1003414 (PMC7682901; doi:10.1371/journal.pmed.1003414)
Supplement: S6 Table — (PDF) [file pmed.1003414.s010.pdf]

S6 Table. Other exposure types in the first 1000 days of life, by level and category of exposure (reported in a single study).

Level	Domain	Type
**Child**	**Fetal growth**	Abdominal circumference [1]
	**Cardiometabolic and inflammatory factors**	11 beta-HSD2 protein [2]; Aortic intima-media thickness [3]; Asymmetric dimethylarginine [4]; Carotid artery resistive index [4]; Carotid tensile stress [5]; Cord plasma cortisol [2]; Cord plasma C-peptide [6]; Cord plasma glucose [6]; Cord plasma HDL [6]; Cord plasma insulin [6]; Cord plasma LDL [6]; Cord plasma total cholesterol [6]; Cord plasma triglycerides [6]; Diastolic blood pressure [1]; Infectious disease occurrence [7]; Left ventricular mass index [6]; Pulse pressure [1]; Systolic blood pressure [1]
	**Epigenetics**	Gene methylation following prenatal exposure to air pollution [8, 9]
	**Other**	1-minute APGAR score [10]; Days in neonatal intensive care unit [10]; Glucocorticoids exposure [10]
**Family**	**Pregnancy-specific factors**	Mode of delivery [11]; Premature rupture of membranes [10]; Spontaneous preterm delivery [10]
	**Maternal weight and growth, nutrition, physical activity**	Maternal carbohydrate intake [12]; Maternal energy from carbohydrates [12]; Maternal energy from protein [12]; Maternal energy from saturated fat [12]; Maternal energy from total fat [12]; Maternal protein intake [12]; Maternal ratio of energy intake to basal metabolic rate [12]; Maternal saturated fat intake [12]; Maternal strenuous exercise [12]; Maternal total energy intake [12]; Maternal total fat intake [12]
	**Cardiometabolic and inflammatory factors**	Maternal plasma cortisol [2]
	**Other**	Maternal Polycystic Ovary Syndrome [13]
**Environmental**	**Air pollution**	NO2 [8]; O3 [8]; PM10 [8]; PM2.5 [8]

References

1. Olander RFW, Sundholm JKM, Ojala TH, Andersson S, Sarkola T. Neonatal Arterial Morphology Is Related to Body Size in Abnormal Human Fetal Growth. Circulation: Cardiovascular Imaging. 2016;9(9). doi: 10.1161/CIRCIMAGING.116.004657.
2. Chen L, Guilmette J, Luo ZC, Cloutier A, Wang WJ, Yang MN, et al. Placental 11beta-HSD2 and Cardiometabolic Health Indicators in Infancy. Diabetes care. 2019. doi: 10.2337/dc18-2041.
3. Sodhi KS, Hondappanavar A, Saxena AK, Dutta S, Khandelwal N. Intima-media complex thickness: Preliminary workup of comparative evaluation of abdominal aorta and carotid artery of small-for-gestation-age term newborns and normal size term newborns. Acta Cardiologica. 2015;70(3):351-7. doi: 10.2143/AC.70.3.3080640.
4. Dilli D, Ozkan E, Ozkan MB, Aydin B, Özyazici A, Fettah N, et al. Umbilical cord asymmetric dimethylarginine levels and ultrasound assessment of carotid arteries in neonates born small for gestational age. Journal of Maternal-Fetal and Neonatal Medicine. 2017;30(4):492-6. doi: 10.1080/14767058.2016.1176136.
5. Stergiotou I, Crispi F, Valenzuela-Alcaraz B, Cruz-Lemini M, Bijnens B, Gratacos E. Aortic and carotid intima-media thickness in term small-for-gestational-age newborns and relationship with prenatal signs of severity. Ultrasound in obstetrics & gynecology : the official journal of the International Society of Ultrasound in Obstetrics and Gynecology. 2014;43(6):625-31. doi: 10.1002/uog.13245.
6. Atabek ME, Çağan HH, Eklioğlu BS, Oran B. Absence of increase in carotid artery Intima-Media thickness in infants of diabetic mothers. JCRPE Journal of Clinical Research in Pediatric Endocrinology. 2011;3(3):144-8. doi: 10.4274/jcrpe.v3i3.28.
7. Prins-Van Ginkel AC, Bruijning-Verhagen PCJ, Wijga AH, Bots ML, Gehring U, Van Der Hoek W, et al. Childhood infections and common carotid intima media thickness in adolescence. Epidemiology and Infection. 2019;147. doi: 10.1017/S095026881800287X.
8. Breton CV, Yao J, Millstein J, Gao L, Siegmund KD, Mack W, et al. Prenatal air pollution exposures, DNA methyl transferase genotypes, and associations with newborn line1 and Alu methylation and childhood blood pressure and carotid intima-media thickness in the children's health study. Environmental Health Perspectives. 2016;124(12):1905-12. doi: 10.1289/EHP181.
9. Breton CV, Gao L, Yao J, Siegmund KD, Lurmann F, Gilliland F. Particulate matter, the newborn methylome, and cardio-respiratory health outcomes in childhood. Environ Epigenet. 2016;2(2):dvw005. doi: 10.1093/eep/dvw005.
10. Rodriguez-Lopez M, Osorio L, Acosta-Rojas R, Figueras J, Cruz-Lemini M, Figueras F, et al. Influence of breastfeeding and postnatal nutrition on cardiovascular remodeling induced by fetal growth restriction. Pediatric research. 2016;79(1):100-6. doi: 10.1038/pr.2015.182.
11. Dratva J, Breton CV, Hodis HN, Mac KWJ, Salam MT, Zemp E, et al. Birth weight and carotid artery intima-media thickness. Journal of Pediatrics. 2013;162(5):906-11.e2. doi: 10.1016/j.jpeds.2012.10.060.
12. Gale CR, Jiang B, Robinson SM, Godfrey KM, Law CM, Martyn CN. Maternal diet during pregnancy and carotid intima-media thickness in children. Arteriosclerosis, thrombosis, and vascular biology. 2006;26(8):1877-82. doi: 10.1161/01.ATV.0000228819.13039.b8.
13. Wilde MAD, Eising JB, Gunning MN, Koster MPH, Evelein AMV, Dalmeijer GW, et al. Cardiovascular and Metabolic Health of 74 Children From Women Previously Diagnosed With Polycystic Ovary Syndrome in Comparison With a Population-Based Reference Cohort. Reproductive Sciences. 2018;25(10):1492-500. doi: 10.1177/1933719117749761.
