# Supplementary material for: Risk factors during first 1,000 days of life for carotid intima-media thickness in infants, children, and adolescents: A systematic review with meta-analyses
Source: PLoS Med. 2020 Nov 23;17(11):e1003414. doi: 10.1371/journal.pmed.1003414 (PMC7682901; doi:10.1371/journal.pmed.1003414)
Supplement: S7 Table — (PDF) [file pmed.1003414.s011.pdf]

**S7 Table. Criteria for study quality assessment.** Using Cochrane collaboration risk of bias tool for interventional studies [1,2] and the Newcastle-Ottawa Scale for observational studies [3].

| INTERVENTIONAL STUDIES | |
|---|---|
| Random sequence generation | Selection bias (biased allocation to interventions) due to inadequate generation of a randomized sequence.<br>Criteria<br>• high: the random component in the sequence generation process was referring to a random number table, using a computer random number generator, coin tossing, shuffling cards or envelopes, throwing dice, or drawing of lots<br>• low: non-random component in the sequence generation process such as generation by odd or even date of birth, some rule based on date (or day) of admission, some rule based on hospital or clinic record number, or allocation by judgment of the clinician, preference of the participant, based on the results of a laboratory test or a series of tests, or availability of the intervention<br>• unclear: insufficient information to permit judgment |
| Allocation concealment | Selection bias (biased allocation to interventions) due to inadequate concealment of allocations prior to assignment.<br>Criteria<br>• high: assignment could not be foreseen by participants and investigators due to central allocation, sequentially numbered drug container of identical appearance, sequentially numbered opaque sealed envelopes<br>• low: assignment could have been foreseen by participants or investigators due to open random allocation schedule (e.g. list of random numbers), assignment envelopes without appropriate safeguards, alternation or rotation, date of birth, case record number, or any other explicitly unconcealed procedure<br>• unclear: insufficient information to permit judgment |
| Blinding participants and personnel | Performance bias due to knowledge of the allocated interventions by participants and personnel during the study.<br>Criteria<br>• high: no blinding or incomplete blinding, but the review authors judge that the outcome is not likely to be influenced by lack of blinding; or blinding of participants and key study personnel ensured, and unlikely that the blinding could have been broken<br>• low: no blinding or incomplete blinding, and the outcome is likely to be influenced by lack of blinding; or blinding of key study participants and personnel attempted, but likely that the blinding could have been broken, and the outcome is likely to be influenced by lack of blinding.<br>• unclear: insufficient information to permit judgment |
| Blinding outcome assessment | Detection bias due to knowledge of the allocated interventions by outcome assessors.<br>Criteria<br>• high: no blinding of outcome assessment, but the review authors judge that the outcome measurement is not likely to be influenced by lack of blinding; or blinding of outcome assessment ensured, and unlikely that the blinding could have been broken.<br>• low: no blinding of outcome assessment, and the outcome measurement is likely to be influenced by lack of blinding; or blinding of outcome assessment, but likely that the blinding could have been broken, and the outcome measurement is likely to be influenced by lack of blinding. |

| | |
|---|---|
| | • unclear: insufficient information to permit judgment |
| Incomplete outcome data | Attrition bias due to amount, nature or handling of incomplete outcome data.<br>Criteria<br>• high: no missing outcome data; or reasons for missing outcome data unlikely to be related to true outcome (for survival data, censoring unlikely to be introducing bias); or missing outcome data balanced in numbers across intervention groups, with similar reasons for missing data across groups; or for dichotomous outcome data, the proportion of missing outcomes compared with observed event risk not enough to have a clinically relevant impact on the intervention effect estimate; or for continuous outcome data, plausible effect size (difference in means or standardized difference in means) among missing outcomes not enough to have a clinically relevant impact on observed effect size; or missing data have been imputed using appropriate methods<br>• low: reason for missing outcome data likely to be related to true outcome, with either imbalance in numbers or reasons for missing data across intervention groups; or for dichotomous outcome data, the proportion of missing outcomes compared with observed event risk enough to induce clinically relevant bias in intervention effect estimate; or for continuous outcome data, plausible effect size (difference in means or standardized difference in means) among missing outcomes enough to induce clinically relevant bias in observed effect size; or 'as-treated' analysis done with substantial departure of the intervention received from that assigned at randomization or; or potentially inappropriate application of simple imputation<br>• unclear: insufficient reporting of attrition/exclusions to permit judgment (e.g. number randomized not stated, no reasons for missing data provided) |
| Selective reporting | Reporting bias due to selective outcome reporting.<br>Criteria<br>• high: the study protocol is available and all of the study's pre-specified (primary and secondary) outcomes that are of interest in the review have been reported in the pre-specified way; or the study protocol is not available but it is clear that the published reports include all expected outcomes, including those that were pre-specified (convincing text of this nature may be uncommon)<br>• low: not all of the study's pre-specified primary outcomes have been reported; or one or more primary outcomes is reported using measurements, analysis methods or subsets of the data (e.g. subscales) that were not pre-specified; or one or more reported primary outcomes were not pre-specified (unless clear justification for their reporting is provided, such as an unexpected adverse effect); or one or more outcomes of interest in the review are reported incompletely so that they cannot be entered in a meta-analysis or; or the study report fails to include results for a key outcome that would be expected to have been reported for such a study<br>• unclear: insufficient information to permit judgment |
| Other bias | Bias due to problems not covered elsewhere in the table.<br>Criteria<br>• high: the study appears to be free of other sources of bias.<br>• low: there is at least one important risk of bias, for example, the study had a potential source of bias related to the specific study design used; or has been claimed to have been fraudulent; or had some other problem<br>• unclear: there may be a risk of bias, but there is either: insufficient information to assess whether an important risk of bias exists; or insufficient rationale or evidence that an identified problem will introduce bias. |

| OBSERVATIONAL STUDIES | |
|---|---|
| Selection of non-exposed | For both cohort and cross-sectional studies.<br>Criteria<br>• high: non-exposed drawn from the same source as the exposed cohort or study performed in a single pool of participants (i.e. when sampling of participants in the study is not based on the exposure status)<br>• low: non-exposed drawn from a different source than the exposed cohort<br>• unclear: insufficient information to permit judgement; no description of the derivation of the non-exposed |
| Ascertainment of outcome | For both cohort and cross-sectional studies.<br>Criteria<br>• high: blind assessment<br>• low: non-blind assessment<br>• unclear: insufficient information to permit judgement |
| Participation rate (adequacy of follow-up) | For cohort studies only.<br>Criteria<br>• high: complete follow-up or subjects lost to follow-up unlikely to introduce bias (i.e. small number lost- ≥ 80% follow-up or follow-up rate corresponding to < 80%, but subjects lost to follow-up were well described and did not differ substantially from those not lost to follow-up)<br>• low: subjects lost to follow-up likely to introduce bias (subjects lost to follow-up differ substantially from those not lost to follow-up or follow-up rate corresponding to < 80% without further description of those lost to follow-up)<br>• unclear: insufficient information to permit judgement |
| Participation rate (non-response rate) | For cross-sectional studies only.<br>Criteria<br>• high: non-response rate ≤ 20%<br>• low: non-response rate ≥ 20%<br>• unclear: insufficient information to permit judgement |
| Ascertainment of exposure | For both cohort and cross-sectional studies.<br>Ascertainment of exposure<br>• high: objective and/or reliable assessment method (e.g. reference to records, clinical assessments by study team)<br>• low: method based on self-reporting (e.g. interview or questionnaire) without independent validation (that is, for example, without reference to another source, such as medical records or registries)<br>• unclear: insufficient information to permit judgement |
| Same method of ascertainment for exposed and non-exposed | For both cohort and cross-sectional studies.<br>Same method of ascertainment for exposed and non-exposed?<br>• high: yes<br>• low: no<br>• unclear: insufficient information to permit judgement |
| Comparability | For both cohort and cross-sectional studies. |

| | |
|---|---|
| | Comparability of exposed and non-exposed for child age and sex, on the basis of the design or analysis:<br>• high: study controls for these factors<br>• low: study does not control for these factors<br>• unclear: insufficient information to permit judgement |

Note: high: high quality and low risk of bias; low: low quality and high risk of bias

## References


1. Higgins JPT, Green S. Cochrane Handbook for Systematic Reviews of Interventions Version 5.1.0: The Cochrane Collaboration; 2011. Available from: www.handbook-5-1.cochrane.org.
2. Higgins JP, Altman DG, Gøtzsche PC, Jüni P, Moher D, Oxman AD, et al. The Cochrane Collaboration's tool for assessing risk of bias in randomised trials. BMJ. 2011;343:d5928. doi: 10.1136/bmj.d5928.
3. Wells G, Shea B, O'Connell D, Peterson J, Welch V, Losos M, et al. The Newcastle-Ottawa Scale (NOS) for assessing the quality of nonrandomised studies in meta-analyses. Available from: http://www.ohri.ca/programs/clinical_epidemiology/oxford.asp (accessed August 2017).
