# Supplementary material for: Risk factors during first 1,000 days of life for carotid intima-media thickness in infants, children, and adolescents: A systematic review with meta-analyses
Source: PLoS Med. 2020 Nov 23;17(11):e1003414. doi: 10.1371/journal.pmed.1003414 (PMC7682901; doi:10.1371/journal.pmed.1003414)
Supplement: S8 Table — (PDF) [file pmed.1003414.s012.pdf]

S8 Table. Assessment of study quality for each intervention type in interventional studies.

Author, year	Random sequence generation	Allocation concealment	Blinding participants and personnel	Blinding outcome assessment	Incomplete data outcome	Selective reporting	Other bias	CIMT acquisition site	CIMT image analysis	CIMT reproducibility assessment
Ayer [1], 2009 (b)	high	high	high	high	high	low	high	higher	higher	higher
Gruszfeld [2], 2015	high	high	high	high	high	low	high	higher	lower	lower

Note: high: high quality and low risk of bias; low: low quality and high risk of bias; higher: higher CIMT quality (reliability); lower: lower CIMT quality (reliability)

References

1. Ayer JG, Harmer JA, Xuan W, Toelle B, Webb K, Almqvist C, et al. Dietary supplementation with n-3 polyunsaturated fatty acids in early childhood: Effects on blood pressure and arterial structure and function at age 8 y. American Journal of Clinical Nutrition. 2009;90(2):438-46. doi: 10.3945/ajcn.2009.27811.
2. Gruszfeld D, Weber M, Nowakowska-Rysz M, Janas R, Kozlik-Feldmann R, Xhonneux A, et al. Protein intake in infancy and carotid intima media thickness at 5 years - A secondary analysis from a randomized trial for the European childhood obesity study group. Annals of Nutrition and Metabolism. 2015;66(1):51-9. doi: 10.1159/000369980.
