# Supplementary material for: Risk factors during first 1,000 days of life for carotid intima-media thickness in infants, children, and adolescents: A systematic review with meta-analyses
Source: PLoS Med. 2020 Nov 23;17(11):e1003414. doi: 10.1371/journal.pmed.1003414 (PMC7682901; doi:10.1371/journal.pmed.1003414)
Supplement: S9 Table — (PDF) [file pmed.1003414.s013.pdf]

S9 Table. Assessment of study quality for observational studies included in meta-analyses.

Author, Year	Selection of non-exposed	Ascertain outcome	Participation rate	Type of exposure	Ascertain exposure	Same method for exposed and non-exposed	Comparability	CIMT acquisition site	CIMT image analysis	CIMT reproducibility assessment
Gale [1], 2006	high	unclear	high	Maternal smoking in pregnancy	low	high	high	higher	higher	higher
Ayer [2], 2009 (a)	high	high	high	Birth weight	high	high	low	higher	higher	higher
				Maternal diabetes in pregnancy	low	high	low			
Ayer [3], 2011	high	high	high	Maternal smoking in pregnancy	low	high	low	higher	higher	higher
Crispi [4], 2010	high	high	high	Prematurity	high	high	low	higher	higher	higher
				Small size for gestational age	high	high	high			
Trevisanuto [5], 2010	high	high	low	Small size for gestational age	unclear	unclear	low	higher	lower	higher
Geerts [6], 2012	high	high	high	Maternal smoking in pregnancy	low	high	high	higher	higher	higher
Atabek [7], 2011	high	high	unclear	Birth length	high	high	low	higher	lower	unclear
				Birth weight	high	high	low			
				Birth head circumference	high	high	low			
				Maternal diabetes in pregnancy	unclear	unclear	low			
Dratva [8], 2013	high	unclear	unclear	Prematurity	high	high	low	higher	higher	higher
Schubert [9], 2013	low	high	unclear	Prematurity	high	high	low	higher	higher	higher
Valenzuela-Alcaraz [10], 2019	high	high	unclear	ART conception	high	high	low	higher	higher	higher

Morsing [11], 2014	low	unclear	low	Prematurity	high	high	high	unclear	lower	unclear
				Small size for gestational age	high	high	high			
Stergiotou [12], 2014	high	unclear	unclear	Small size for gestational age	high	high	high	higher	higher	higher
Gruszfeld [13], 2015	high	high	low	Birth weight	high	high	low	higher	lower	lower
Sebastiani [14], 2016	high	unclear	low	Small size for gestational age	high	high	high	higher	unclear	higher
Mohlkert [15], 2017	low	high	high	Prematurity	high	high	high	higher	higher	unclear
Tzschoppe [16], 2017	high	unclear	low	Small size for gestational age	high	high	low	unclear	unclear	unclear
Sebastiani [17], 2019	high	unclear	low	Small size for gestational age	high	high	high	higher	unclear	higher
Sundholm [18], 2019	low	high	low	Maternal diabetes in pregnancy	high	high	unclear	higher	unclear	higher
Jouret [19], 2011	low	unclear	unclear	Small size for gestational age	high	unclear	high	unclear	unclear	unclear
Scherrer [20], 2012	low	high	unclear	ART conception	unclear	unclear	high	unclear	higher	higher
de Arriba [21], 2013	high	unclear	unclear	Birth length	unclear	unclear	low	higher	unclear	unclear
				Birth weight	unclear	unclear	low			
				Small size for gestational age	unclear	unclear	low			
Maurice [22], 2014	high	unclear	unclear	Small size for gestational age	high	high	low	higher	higher	unclear
Xu [23], 2014	unclear	high	unclear	ART conception	high	high	low	unclear	unclear	unclear
Sodhi [24], 2015	high	high	unclear	Birth length	high	high	unclear	unclear	lower	lower
				Birth weight	high	high	unclear			
				Birth head circumference	high	high	unclear			
				Small size for gestational age	high	high	low			

Ciccone [25], 2016	high	high	unclear	Prematurity	high	high	high	higher	unclear	unclear
Faienza [26], 2016	low	unclear	unclear	Small size for gestational age	unclear	unclear	high	unclear	lower	higher
Olander [27], 2016	high	unclear	unclear	Birth weight	high	high	low	higher	lower	higher
				Birth head circumference	high	high	low			
				Small size for gestational age	high	high	low			
Dilli [28], 2017	high	high	unclear	Birth weight	high	high	low	higher	higher	higher
				Small size for gestational age	high	high	low			
Stock [29], 2018	high	unclear	low	Prematurity	high	high	low	higher	lower	unclear
				Small size for gestational age	high	high	low			
Muñiz Fontán [30], 2019	high	high	unclear	Small size for gestational age	unclear	unclear	high	higher	higher	unclear

Note: high: high quality and low risk of bias; low: low quality and high risk of bias; higher: higher CIMT quality (reliability); lower: lower CIMT quality (reliability)
Abbreviations: ART, assisted reproductive technology.

References

1. Gale CR, Jiang B, Robinson SM, Godfrey KM, Law CM, Martyn CN. Maternal diet during pregnancy and carotid intima-media thickness in children. Arteriosclerosis, thrombosis, and vascular biology. 2006;26(8):1877-82. doi: 10.1161/01.ATV.0000228819.13039.b8.
2. Ayer JG, Harmer JA, Nakhla S, Xuan W, Ng MKC, Raitakari OT, et al. HDL-cholesterol, blood pressure, and asymmetric dimethylarginine are significantly associated with arterial wall thickness in children. Arteriosclerosis, thrombosis, and vascular biology. 2009;29(6):943-9. doi: 10.1161/ATVBAHA.109.184184.
3. Ayer JG, Belousova E, Harmer JA, David C, Marks GB, Celermajer DS. Maternal cigarette smoking is associated with reduced high-density lipoprotein cholesterol in healthy 8-year-old children. European heart journal. 2011;32(19):2446-53. doi: 10.1093/eurheartj/ehr174.
4. Crispi F, Bijnens B, Figueras F, Bartrons J, Eixarch E, Le Noble F, et al. Fetal growth restriction results in remodeled and less efficient hearts in children. Circulation. 2010;121(22):2427-36. doi: 10.1161/circulationaha.110.937995.
5. Trevisanuto D, Avezzú F, Cavallin F, Doglioni N, Marzolo M, Verlato F, et al. Arterial wall thickness and blood pressure in children who were born small for gestational age: Correlation with umbilical cord high-sensitivity C-reactive protein. Archives of disease in childhood. 2010;95(1):31-4. doi: 10.1136/adc.2008.150326.
6. Geerts CC, Bots ML, Van Der Ent CK, Grobbee DE, Uiterwaal CSPM. Parental smoking and vascular damage in their 5-year-old children. Pediatrics. 2012;129(1):45-54. doi: 10.1542/peds.2011-0249.
7. Atabek ME, Çağan HH, Eklioğlu BS, Oran B. Absence of increase in carotid artery Intima-Media thickness in infants of diabetic mothers. JCRPE Journal of Clinical Research in Pediatric Endocrinology. 2011;3(3):144-8. doi: 10.4274/jcrpe.v3i3.28.
8. Dratva J, Breton CV, Hodis HN, Mac KWJ, Salam MT, Zemp E, et al. Birth weight and carotid artery intima-media thickness. Journal of Pediatrics. 2013;162(5):906-11.e2. doi: 10.1016/j.jpeds.2012.10.060.
9. Schubert U, Müller M, Abdul-Khaliq H, Norman M, Bonamy AKE. Relative intima-media thickening after preterm birth. Acta Paediatrica, International Journal of Paediatrics. 2013;102(10):965-9. doi: 10.1111/apa.12355.
10. Valenzuela-Alcaraz B, Serafini A, Sepulveda-Martinez A, Casals G, Rodriguez-Lopez M, Garcia-Otero L, et al. Postnatal persistence of fetal cardiovascular remodelling associated with assisted reproductive technologies: a cohort study. Bjog. 2019;126(2):291-8. doi: 10.1111/1471-0528.15246.
11. Morsing E, Liuba P, Fellman V, Maršál K, Brodszki J. Cardiovascular function in children born very preterm after intrauterine growth restriction with severely abnormal umbilical artery blood flow. European journal of preventive cardiology. 2014;21(10):1257-66. doi: 10.1177/2047487313486044.
12. Stergiotou I, Crispi F, Valenzuela-Alcaraz B, Cruz-Lemini M, Bijnens B, Gratacos E. Aortic and carotid intima-media thickness in term small-for-gestational-age newborns and relationship with prenatal signs of severity. Ultrasound in obstetrics & gynecology : the official journal of the International Society of Ultrasound in Obstetrics and Gynecology. 2014;43(6):625-31. doi: 10.1002/uog.13245.

13. Gruszfeld D, Weber M, Nowakowska-Rysz M, Janas R, Kozlik-Feldmann R, Xhonneux A, et al. Protein intake in infancy and carotid intima media thickness at 5 years - A secondary analysis from a randomized trial for the European childhood obesity study group. Annals of Nutrition and Metabolism. 2015;66(1):51-9. doi: 10.1159/000369980.
14. Sebastiani G, Díaz M, Bassols J, Aragonés G, López-Bermejo A, de Zegher F, et al. The sequence of prenatal growth restraint and post-natal catch-up growth leads to a thicker intima-media and more pre-peritoneal and hepatic fat by age 3–6 years. Pediatric Obesity. 2016;11(4):251-7. doi: 10.1111/ijpo.12053.
15. Mohlkert LA, Hallberg J, Broberg O, Hellström M, Pegelow Halvorsen C, Sjöberg G, et al. Preterm arteries in childhood: Dimensions, intima-media thickness, and elasticity of the aorta, coronaries, and carotids in 6-y-old children born extremely preterm. Pediatric research. 2017;81(2):299-306. doi: 10.1038/pr.2016.212.
16. Tzschoppe A, Von Kries R, Struwe E, Rascher W, Dörr HG, Jüngert J, et al. Intrauterine Growth Restriction (IUGR) Induces Signs of Subclinical Atherosclerosis in 6-Year-Old Infants Despite Absence of Excessive Growth. Klinische Padiatrie. 2017;229(4):209-15. doi: 10.1055/s-0043-104528.
17. Sebastiani G, García-Beltran C, Pie S, Guerra A, López-Bermejo A, de Toledo JS, et al. The sequence of prenatal growth restraint and postnatal catch-up growth: normal heart but thicker intima-media and more pre-peritoneal fat in late infancy. Pediatric Obesity. 2019;14(3). doi: 10.1111/ijpo.12476.
18. Sundholm JKM, Litwin L, Rönö K, Koivusalo SB, Eriksson JG, Sarkola T. Maternal obesity and gestational diabetes: Impact on arterial wall layer thickness and stiffness in early childhood - RADIEL study six-year follow-up. Atherosclerosis. 2019. doi: 10.1016/j.atherosclerosis.2019.01.037.
19. Jouret B, Dulac Y, Bassil Eter R, Taktak A, Cristini C, Lounis N, et al. Endothelial function and mechanical arterial properties in children born small for gestational age: comparison with obese children. Hormone research in paediatrics. 2011;76(4):240-7. doi: 10.1159/000329379.
20. Scherrer U, Rimoldi SF, Rexhaj E, Stuber T, Duplain H, Garcin S, et al. Systemic and pulmonary vascular dysfunction in children conceived by assisted reproductive technologies. Circulation. 2012;125(15):1890-6. doi: 10.1161/CIRCULATIONAHA.111.071183.
21. de Arriba A, Domínguez M, Labarta JI, Puga B, Mayayo E, Longás AF. Metabolic syndrome and endothelial dysfunction in a population born small for gestational age relationship to growth and Gh therapy. Pediatric endocrinology reviews : PER. 2013;10(3):297-307.
22. Maurice RL, Vaujois L, Dahdah N, Chibab N, Maurice A, Nuyt AM, et al. Carotid wall elastography to assess midterm vascular dysfunction secondary to intrauterine growth restriction: Feasibility and comparison with standardized intima-media thickness. Ultrasound in Medicine and Biology. 2014;40(5):864-70. doi: 10.1016/j.ultrasmedbio.2013.11.013.
23. Xu GF, Zhang JY, Pan HT, Tian S, Liu ME, Yu TT, et al. Cardiovascular dysfunction in offspring of ovarian-hyperstimulated women and effects of estradiol and progesterone: A retrospective cohort study and proteomics analysis. Journal of Clinical Endocrinology and Metabolism. 2014;99(12):E2494-E503. doi: 10.1210/jc.2014-2349.

24. Sodhi KS, Hondappanavar A, Saxena AK, Dutta S, Khandelwal N. Intima-media complex thickness: Preliminary workup of comparative evaluation of abdominal aorta and carotid artery of small-for-gestation-age term newborns and normal size term newborns. Acta Cardiologica. 2015;70(3):351-7. doi: 10.2143/AC.70.3.3080640.
25. Ciccone MM, Cortese F, Gesualdo M, A DIM, Tafuri S, Mancini G, et al. The role of very low birth weight and prematurity on cardiovascular disease risk and on kidney development in children: a pilot study. Minerva Pediatr. 2016.
26. Faienza MF, Brunetti G, Delvecchio M, Zito A, de Palma FD, Cortese F, et al. Vascular function and myocardial performance indices in children born small for gestational age. Circulation Journal. 2016;80(4):958-63. doi: 10.1253/circj.CJ-15-1038.
27. Olander RFW, Sundholm JKM, Ojala TH, Andersson S, Sarkola T. Neonatal Arterial Morphology Is Related to Body Size in Abnormal Human Fetal Growth. Circulation: Cardiovascular Imaging. 2016;9(9). doi: 10.1161/CIRCIMAGING.116.004657.
28. Dilli D, Ozkan E, Ozkan MB, Aydin B, Özyazici A, Fettah N, et al. Umbilical cord asymmetric dimethylarginine levels and ultrasound assessment of carotid arteries in neonates born small for gestational age. Journal of Maternal-Fetal and Neonatal Medicine. 2017;30(4):492-6. doi: 10.1080/14767058.2016.1176136.
29. Stock K, Schmid A, Griesmaier E, Gande N, Hochmayr C, Knoflach M, et al. The Impact of Being Born Preterm or Small for Gestational Age on Early Vascular Aging in Adolescents. The Journal of pediatrics. 2018;201:49-54.e1. doi: 10.1016/j.jpeds.2018.05.056.
30. Muñiz Fontán M, Oulego Erroz I, Revilla Orias D, Muñoz Lozón A, Rodriguez Núñez A, Lurbe IFE. Thoracic Aortic Intima-Media Thickness in Preschool Children Born Small for Gestational Age. Journal of Pediatrics. 2019. doi: 10.1016/j.jpeds.2018.12.037.
