# Supplementary material for: Risk factors during first 1,000 days of life for carotid intima-media thickness in infants, children, and adolescents: A systematic review with meta-analyses
Source: PLoS Med. 2020 Nov 23;17(11):e1003414. doi: 10.1371/journal.pmed.1003414 (PMC7682901; doi:10.1371/journal.pmed.1003414)
Supplement: Attachment — Submitted filename: epure.pdf [file pmed.1003414.s014.pdf]

This paper reports a systematic review with meta–analysis of studies of carotid intima–media thickness in childhood.

I was asked for a statistical report and I interpret that to include all aspects of the design and conduct of the study.

Points of detail

Page 2 Here and throughout the authors use the prime symbol to separate the thousands digit from the hundreds. I think PLOS uses the Anglo–Saxon convention of the comma.

Page 8 If the authors had found an existing review would they have used it as a source of possible studies? There is, however, a strong line that one should never even read previous reviews in case one imports their biases. Is that what the authors mean?

Page 8 The authors have excluded studies which were in children with certain rare conditions. This does raise the question of what the entry criteria were for the cohorts they do use. Did these all recruit unselected general population children? This does not seem to be stated anywhere unless I have missed it.

Page 9 The language restriction seems to have excluded 3 studies (page 43, Figure 1). At the moment the coverage of the review is largely limited to Europe. I am not a subject matter expert so whether this limitation matters I cannot tell but I think we should at least know which these excluded studies were and whether they would have enhanced global coverage. If cardiovascular disease does vary substantially world–wide perhaps a more extended search would be valuable as various empirical studies have indicated that the main bibliographic databases have varied and incomplete coverage of material not in English (Pilkington et al., 2005), and have limited coverage of material from low and middle–income country journals (Kieling et al., 2009; Syed Sheriff et al., 2008).

Page 11 We read how extraction disagreements were dealt with but not quality disagreement.

Page 11 I think it might be clearer to cite each methods paper after the technique to which it refers. So, for instance, cite reference 30 immediately after the relevant text about range and inter–quartile range. Incidentally the authors might be interested in Luo et al. (2018) where

a team incorporating many of reference 30's authors update the methods.

Page 12 Choosing the random effects model a priori is clearly a good idea and better than choosing it based on estimated obtained heterogeneity.

Page 12 I would usually suggest that confidence intervals for I^2 would also be helpful to reveal our uncertainty about the heterogeneity. I know we did not use to use them but I am persuaded by Ioannidis et al. (2007) that we should. In this case though I think the degree of (expected) heterogeneity is such that I would not insist although the confidence intervals might be a useful addition.

Page 14 Some of the moderators displayed in Table 4 are inherently continuous like age and sample size and presumably transducer frequency. It might be better to do a meta–regression using the moderators as continuous. Are any of these ecological analyses? Sample size presumably not but what about age?

Page 41 and 42 Why does 'not specified' become classified the way it does? This need justifying.

Supplementary Figure S2 This does not seem to be called out anywhere in the main text. The term small study effects used in the caption is better than the term publication bias used in the section heading. If the authors are purely concerned with asymmetry as tested by the

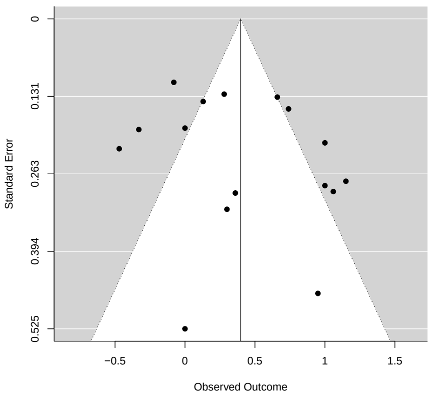

Figure 1: Conventional plot of SGA studies

Eggers' test then I would have thought a conventional funnel plot with the funnel symmetric about the estimate as shown in my figure rather than the Peters' contour–enhanced plot (Peters et al., 2008) might have

> been better. What the Peters' plot seems to show in sub–figure (a) is that if there are any missing studies they are in regions of possible concern (the grey ones). What message wold the authors ike us to draw from the Peters' plots?

The article gives us a commendable amount of detail but I miss the actual means and standard deviations from the primary studies. These would be helpful for anyone who want to do a different analysis.

Summary

Mostly comments about clarification and some suggestions for additions. This is a very comprehensive report with little missing.

Michael Dewey

References

J Ioannidis, N Patsopoulos, and E Evangelou. Uncertainty in heterogeneity estimates in meta–analyses. *British Medical Journal*, 335:914–916, 2007.

C Kieling, H Herrman, V Patel, and J J Mari. Indexation of psychiatric journals from low– and middle–income countries: a survey and a case study. *World Psychiatry*, 8:40–44, 2009.

D Luo, X Wan, J Liu, and T Tong. Optimally extracting the sample mean from the sample size, median, mid–range, and/or mid–quantile range. *Statistical Methods in Medical Research*, 27:1785–1805, 2018.

J L Peters, A J Sutton, D R Jones, K R Abrams, and L Rushton. Contour–enhanced meta–analysis funnel plots help distinguish publication bias from other causes of asymmetry. *Journal of Clinical Epidemiology*, 61:991–996, 2008.

K Pilkington, A Boshnakova, M Clarke, and J Richardson. 'No language restrictions' in database searches: what does this really mean? *The Journal of Alternative and Complementary Medicine*, 11:205–207, 2005.

R J Syed Sheriff, C E Adams, P Tharyan, M Jayaram, L Duley, and the PRACTIHC Mental Health Group. Randomised trials relevant to mental health conducted in low and middle–income countries: a survey. *BMC Psychiatry*, 8(69), 2008.
