# Supplementary material for: Risk factors during first 1,000 days of life for carotid intima-media thickness in infants, children, and adolescents: A systematic review with meta-analyses
Source: PLoS Med. 2020 Nov 23;17(11):e1003414. doi: 10.1371/journal.pmed.1003414 (PMC7682901; doi:10.1371/journal.pmed.1003414)
Supplement: Attachment — Submitted filename: response_to_editors_and_reviewers.docx [file pmed.1003414.s015.docx]

To the Editors and Reviewers,

We thank the editors and reviewers for their thorough assessment of our manuscript. We are pleased to present a version that has been strengthened by your comments and suggestions. The revision required major changes that were highlighted in the marked-up copy of the manuscript using the “Track Changes” option in Microsoft Word. The clean version of the manuscript has been submitted as the main article file and has been formatted according to the formatting guidelines of PLOS Medicine.

We have thoroughly addressed all points raised by the three reviewers. We explained our approach and methods in detail, conducted further analyses, performed major modifications to the text, and provided more material in the supporting information file.

Dr. Sarkola (reviewer 2), a well-known expert in pediatric cardiology, stated that the review contains the most relevant articles in the field. Reviewer 3 asked, however, to update the review. We are not convinced that it is useful to update the review at this time point. Doing it meticulously requires a lot of resources, but would probably not improve the relevancy or change the conclusions of our study. It would, however, delay the publication. We thank you for your understanding.

Sincerely,

Dr. Adina Mihaela Epure, Dr. Arnaud Chiolero, and Dr. Nicole Sekarski

**EDITORS**

*Comment: In the last sentence of the Abstract Methods and Findings section, please add a brief note commenting on any key limitation(s) of the study's methodology.*

**Response:** As requested, we added a brief note on the key limitations in the last sentence of the abstract’s Methods and Findings section: “*The degree of confidence in results is limited by the low number of studies with relatively small sample sizes and the high between-study heterogeneity”*.

**REVIEWER #1: Michael Dewey**

GENERAL REMARKS

*This paper reports a systematic review with meta-analysis of studies of carotid intima-media thickness in childhood. I was asked for a statistical report and I interpret that to include all aspects of the design and conduct of the study.*

POINTS OF DETAIL

1. *Comment: Page 2 Here and throughout the authors use the prime symbol to separate*

*the thousands digit from the hundreds. I think PLOS uses the Anglo-Saxon convention of the comma.*

**Response:** We thank Prof. Dewey for pointing this out and we revised the whole manuscript accordingly.

2. *Comment: Page 8 If the authors had found an existing review would they have used*

*it as a source of possible studies? There is, however, a strong line that one should never even read previous reviews in case one imports their biases. Is that what the authors mean?*

**Response:** We thank Prof. Dewey for underscoring this. Indeed, we did not base our review on an existing systematic review in terms of methodology or source of studies. To the best of our knowledge, this is the first systematic review with such a multidimensional view on risk factors in the first 1000 days of life and CIMT in children.

3. *Comment: Page 8 The authors have excluded studies which were in children with certain rare conditions. This does raise the question of what the entry criteria were for the cohorts they do use. Did these all recruit unselected general population children? This does not seem to be stated anywhere unless I have missed it.*

**Response:** We thank Prof. Dewey for pointing out that this was unclear. Studies in children with rare or special conditions in the first 1000 days of life, such as congenital heart disease, were excluded because we considered that there might be a different effect of DOHAD factors in these groups than in generally healthy children or the general population and this should be the subject of a separate systematic review. Further, we aimed to infer our results to the general population. We revised the text in the Methods section to better justify the eligibility criteria for participants in terms of their health status: *“Studies in children with rare or special conditions, such as congenital heart diseases, or postnatal conditions conferring an increased risk of CVD, such as hypertension or diabetes, were not considered for inclusion. We aimed to make inferences to the general population. Further, we assumed that there may be a different effect of DOHAD exposures in these groups than in the general population, which should be evaluated separately”.*

4. *Comment: Page 9 The language restriction seems to have excluded 3 studies (page 43,*

*Figure 1). At the moment the coverage of the review is largely limited to Europe. I am not a subject matter expert so whether this limitation matters I cannot tell but I think we should at least know which these excluded studies were and whether they would have enhanced global coverage. If cardiovascular disease does vary substantially world-wide perhaps a more extended search would be valuable as various empirical studies have indicated that the main bibliographic databases have varied and incomplete coverage of material not in English (Pilking-ton et al., 2005), and have limited coverage of material from low and middle-income country journals (Kieling et al., 2009; Syed Sheri et al.,2008).*

**Response:** Indeed, most of the studies included in this systematic review were conducted in Europe, but we did not restrict searches to specific regions defined by geographic or income criteria. Nonetheless, as Prof. Dewey pointed out, we were limited by the language of publication because English and French were the two languages spoken in common by the three reviewers who performed screening. The three studies eliminated due to language were published in Spanish, Russian, and Polish, therefore it is unlikely that they would have significantly enhanced the global coverage of the review. Moreover, to overcome the language restriction, we searched several databases, such as Medline, Embase, Central, Web of Science, Google Scholar, and 3 trial registers. To clarify the three language exclusions, we revised the exclusion reason in Figure 1 to say *3 in Spanish, Russian, Polish* instead of *3 in language other than English and French*.

5. *Comment: Page 11 We read how extraction disagreements were dealt with but not quality disagreement.*

**Response:** We revised the text to clarify that quality assessments were performed independently by two reviewers.

6. *Comment: Page 11 I think it might be clearer to cite each methods paper after the technique to which it refers. So, for instance, cite reference 30 immediately after the relevant text about range and inter-quartile range. Incidentally the authors might be interested in Luo et al. (2018) where a team incorporating many of reference 30's authors update the methods.*

**Response:** We cited each methods paper after the technique to which it refers.

7. *Comment: Page 12 Choosing the random effects model a priori is clearly a good idea and better than choosing it based on estimated obtained heterogeneity.*

**Response:** We thank Prof. Dewey for this positive comment.

8. *Comment: Page 12 I would usually suggest that confidence intervals for I^2^ would also be helpful to reveal our uncertainty about the heterogeneity. I know we did not use to use them but I am persuaded by Ioannidis et al. (2007) that we should. In this case though I think the degree of (expected) heterogeneity is such that I would not insist although the confidence intervals might be a useful addition.*

**Response:** We thank Prof. Dewey for this suggestion and we agree that in this case the degree of (expected) heterogeneity is that high that the confidence intervals for I^2^ would not change the interpretation of results. To maintain a good readability of the manuscript, we preferred not to add the confidence intervals.

9. *Comment: Page 14 Some of the moderators displayed in Table 4 are inherently continuous like age and sample size and presumably transducer frequency. It might be better to do a meta-regression using the moderators as continuous. Are any of these ecological analyses? Sample size presumably not but what about age?*

**Response:** We agree with Prof. Dewey that it is better to do a meta-regression analysis whenever moderators are inherently continuous. However, as pointed out by Prof. Dewey, some of the moderators, such as transducer frequency and age, were based on ranges of values, which made them unsuitable for meta-regression. Additionally, meta-regression requires a minimum of 10 studies, criterion met by only one of the risk factors included in our systematic review, namely small size for gestational age (N=16 studies). We performed a meta-regression analysis based on sample size, for the association of small size for gestational age with CIMT, and we present the findings in the supplementary file, Supplemental Figure S3. The sample size explained a very small part of the variance (2%).

10. *Comment: Page 41 and 42 Why does `not specified' become classified the way it does? This need justifying.*

**Response:** We agree with Prof. Dewey that the way “not specified” was classified is not common practice and may be disputable. Therefore, we revised our subgroup-analyses in Table 4, separating the category “not specified” from the others. This modification did not change the interpretation of our findings.

11. *Comment: Supplementary Figure S2 This does not seem to be called out anywhere in the main text. The term small study effects used in the caption is better than the term publication bias used in the section heading. If the authors are purely concerned with asymmetry as tested by the Eggers' test then I would have thought a conventional funnel plot with the funnel symmetric about the estimate as shown in my figure rather than the Peters' contour-enhanced plot (Peters et al., 2008) might have been better. What the Peters' plot seems to show in sub-figure (a) is that if there are any missing studies they are in regions of possible concern (the grey ones). What message would the authors like us to draw from the Peters' plots?*

**Response:** We agree with Prof. Dewey that the conventional funnel plots would provide a better visualization of the asymmetry and we revised our Supplementary Figure S2 accordingly. We cited this figure in the main text, Discussion section, paragraph on limitations. We added the term “small study effects” in the heading of the figure. We preferred to also keep the term “publication bias” as it is a subtype of bias that has a straightforward meaning for a large audience.

12. *Comment: The article gives us a commendable amount of detail but I miss the actual means and standard deviations from the primary studies. These would be helpful for anyone who want to do a different analysis.*

**Response:** The means and standard deviations were not systematically provided for the exposed and reference groups in all studies. Therefore, in some cases, we estimated the standardized mean differences from other statistics, for instance, medians and interquartile ranges (Wan et al., 2014) or unstandardized regression coefficient and full-sample standard deviation (Lipsey and Wilson, 2000). We stated in the Discussion section, the paragraph on limitations, that the heterogeneity in reporting of association measures and other summary statistics across studies is a limitation of the review.

13. *Comment: Mostly comments about clarification and some suggestions for additions. This is a very comprehensive report with little missing.*

**Response:** We thank Prof. Dewey for this positive comment.

**REVIEWER #2: Dr Taisto Sarkola**

GENERAL REMARKS

*This is a systematic review and meta-analysis titled "First 1000 days risk factors for carotid intima media thickness in childhood: a systematic review and meta-analysis" assessing different DOHaD variables associations with CIMT mainly during childhood (<10y). The protocol has been published previously (Epure AM, Leyvraz M,Mivelaz Y, et al. Risk factors and determinants of carotid intimamedia thickness in children: protocol for a systematic review and meta-analysis. BMJ Open 2018;0:e019644). The focus from the original protocol (pediatric age group 0-18; postnatal child CVD risk factors obesity, hypertension, smoking, disease and treatments) has changed in the current review manuscript to focus on younger pediatric age and the role of early life DOHaD predictors.*

*The title uses the term "risk factors for CIMT in childhood", but as there is currently no evidence linking CIMT in infancy/childhood to CVD disease later in life, I would certainly propose a more comprehensive vascular phenotype (not only CIMT) in children. Furthermore, if the main focus and aim is to explore relations between DOHaD and vascular changes in children, one would perhaps expect a more comprehensive set of vascular variables included (other sites of intima-media thickness, different measures of arterial function such as regional and local stiffness, blood pressure and heart rate, perhaps also endothelial function for older children) and a more comprehensive set of covariates on postnatal anthropometrics, growth, body composition, and different traditional aspects of CVR (diet, activity etc.). The present manuscript is, thus, limited to child CIMT only, and mainly prenatal, perinatal factors and early life DOHaD factors. In addition, the high heterogeneity and apparent problems in the reported CIMT measurement methodology in infancy/childhood makes the analysis with DOHaD variables and conclusions thereof challenging and uncertain indeed.*

*The CIMT methodology issues warranted the investigators to critically evaluate and explore explanations for this variance, as mentioned in the published protocol as well. This is warmly endorsed by this reviewer, and forms a major strength of this manuscript. The manuscript provides in-depth analyses to shed light on factors related with this heterogeneity and how this might influence the DOHaD analyses. The current guidelines on vascular assessments and reports of population and healthy CIMT references in the pediatric age group have been made to address mainly adolescent populations with CVR and different disease states and treatments affecting the vasculature. This review is welcome in attempting to provide a summary of the very diverse current literature of the ultrasound CIMT during infancy and childhood.*

*The manuscript reads well, many tables and figures are included and the authors have made good attempts in trying to address the objectives. The review also seems to include the most relevant articles in the field. There are, however, some concerns related with method presentation and the analyses and the conclusions that warrant commenting and modifications to the presentation, as outlined in the comments to the authors.*

**Response:** We thank Dr. Sarkola for their favorable comments and appreciate their support for our critical appraisal of the CIMT measurement methods and the relevancy of our systematic review. Regarding some of the general comments stated above, we agree that this systematic review is limited to the relationship of CIMT with multiple early life DOHAD factors. We did not aim to address other vascular phenotypes with this study, but we will bear in mind Dr Sarkola’s suggestion for a separate systematic review. We also agree with the reviewer’s conclusion that the high heterogeneity between studies and the apparent problems in the reported CIMT measurement methodology made this study’s analyses and conclusions challenging and uncertain. Further, we would like to thank Dr. Sarkola for pointing out other high-quality papers in the field that we cited, helping us to improve our discussion. We addressed all specific points of concern below.

POINTS OF DETAIL

1. *Comment: Title and abstract. See general commenting and suggestion above for revising wording in title. Mean difference 0.40. Please provide information on adjustments for body size, HC size, gestational age, child age made in this main result, as appropriate. The first sentence in the conclusion: "Several risk factors in the first 1000 days of life may be associated with increased CIMT during childhood", comments on ART and smoking (tendencies only) also seem poorly substantiated. What intervention?*

**Response:**

We revised the wording in the title as follows: *“First 1000 days risk factors for carotid intima-media thickness in infants, children, and adolescents: a systematic review with meta-analyses”*.

We revised the text in the abstract’s *Methods and findings* section to clarify that “*Estimates from bivariate or partial associations that were least adjusted for sex were used for pooling data across studies, when appropriate, using random-effects meta-analyses*.” For further details with regard to adjustments, please see our response below at comment no. 9 (Data analysis). We revised the description of our results on ART and smoking as follows: “*Children conceived through assisted reproductive technologies […] or exposed to maternal smoking during pregnancy […] had, on average, an increased CIMT, but the imprecision around the estimates was high.*”. We revised the text in the conclusion as follows: “*…further high-quality studies are needed to justify intervention for primordial prevention of cardiovascular disease”.*

2. *Comment: Introduction. Please see general commenting above. The intro could focus more on the objective, i.e. 1) relations with DOHad and 2)* *challenges with ultrasound derived pediatric IMT in order to provide a rationale for the study. The second paragraph could be more focused on the main objective. What is known about the role of early life factors, early child vascular phenotype assessment, and later CVD, and why should the vascular phenotype be assessed at this stage? The last sentence relating to the guidelines could emphasize the difference in age group addressed in the guidelines compared with the focus of the present manuscript.*

**Response:** We thank Dr. Sarkola for these suggestions. We revised the structure of the introduction and we consider that it currently provides a logical and concise flow of information, namely- 1^st^ paragraph: developmental programming and long-term cardiovascular risk, 2^nd^ paragraph: DOHAD factors and early and later life CIMT, 3^rd^ paragraph: CIMT methodology issues. We revised the 2^nd^ and 3^rd^ paragraphs along Dr Sarkola’s suggestions.

*“INTRODUCTION*

*Background*

*Within a Developmental Origins of Health and Disease (DOHaD) framework, the first 1000 days of life, that is, the period from conception to age 2 years, is regarded as a critical period for long-term cardiovascular health (Hanson and Gluckman, 2014). Evidence suggests that adaptations in body structure and function during development, as a response to cues from the environment, for instance, assisted reproductive treatment for conception (Fleming et al., 2018), undernutrition or environmental pollutants (Huxley et al., 2000), could increase the risk for cardiovascular disease (CVD). Indeed, several studies showed that a low birth weight is associated with higher blood pressure (Huxley et al., 2000) or diabetes risk (Tian et al., 2019), as well as with cardiovascular mortality in adulthood (Risnes et al., 2011).*

*Carotid intima-media thickness (CIMT) is a marker of CVD risk linked to pathways that originate in the first 1000 days of life. In adults, CIMT is predictive of heart attack and stroke risk (Lorenz et al., 2007, Eikendal et al., 2015) and is also associated with prenatal risk factors, such as impaired fetal growth and preterm birth (Skilton et al., 2011, Oren et al., 2004). Within a DOHaD perspective, these associations may be explained by early life adaptative changes in the vascular phenotype (Visentin et al., 2014), with lifelong effects on the CVD risk. Nonetheless, in children, the evidence on increased CIMT after impaired fetal growth or preterm birth is inconsistent (Crispi et al., 2010, Dratva et al., 2013, Jouret et al., 2011, Stock et al., 2018). Further, CVD risk is determined by a combination of factors, at multiple levels, rather than a single factor (Hanson and Gluckman, 2011). A large range of early life factors at the child, familial, or environmental levels needs to be explored to better understand CIMT changes in children. To our knowledge, such a comprehensive overview is lacking.*

*CIMT assessment in children can be challenging (Dalla Pozza et al., 2015, Urbina et al., 2009). Several aspects related to the ultrasound equipment (Sarkola et al., 2010, Sarkola et al., 2012, Potter et al., 2008), the site of measurement, or the edge detection approach may influence the reliability of measurements (Urbina et al., 2009, de Groot et al., 2004). Further, measurements in infants and young children are challenging due to limited compliance, anatomic particularities, and a lack of recommendations tailored to this age group (Mivelaz et al., 2016, Dalla Pozza et al., 2015). The comparability of studies is therefore limited and the inconsistency in findings may be attributed, at least in part, to the heterogeneity in CIMT measurement. An appraisal of the CIMT measurement methods in children is needed to shed light on these limitations.*

*Objectives*

*We therefore aimed to 1) assess the relationship between exposures or interventions in the first 1000 days of life and CIMT in childhood and 2) critically appraise the CIMT measurement*

*methods through a systematic review with meta-analyses.”*

3. *Comment: Methods. Methods/Eligibility criteria/Participants. Definition of child population. "Apparently healthy children" vs children with different disease states? On the other hand, "Both apparently healthy children and individuals with clinical conditions will be included." (information from PROSPERO-registry). And furthermore, in published study protocol: "**We will include studies in children from birth up to 18 years. Studies with both children and adults will be included if the data for children can be extracted separately. Both apparently healthy children and subjects with clinical conditions will be included". The manuscript, however, states, "Studies in children with postnatal conditions that confer an increased risk for CVD, e.g. hypertension or diabetes, were excluded. Additionally, children with rare conditions (e.g. congenital heart disease) were not included as they are not representative of the general population". Both these exclusion criteria seem irrational as future cardiovascular disease is of major interest in and early development and interventions performed could well alter both. In addition, the study includes analyses related with maternal gestational risk (egg gestational diabetes), premature birth, abnormal fetal growth, that are per se risk factors for later disease development in offspring. What is the rationale for these exclusion statements? Figure 1 shows that adult populations were excluded. Study population could then be better defined in methods and mentioned in title/abstract as well to avoid confusion.*

**Response:** We thank Dr. Sarkola for pointing out that these exclusions were not clearly justified. This systematic review focused on CIMT in the pediatric population, aged 0 to 18 years. We, thus, never intended to include adults. Studies in mixed populations, i.e., studies comprising both children and adults, were excluded if data could not be extracted separately for children. This decision is per our study protocol (Epure et al., 2018). What is, indeed, different compared to our study protocol is the restriction to exposures or interventions occurring in the first 1,000 days of life, which justify, among other reasons, the exclusions with regard to the health status of the study population. Studies in children with rare conditions in the first 1000 days of life or postnatal conditions conferring an increased risk for CVD were excluded because we considered that there might be a different effect of DOHAD factors in these groups than in generally healthy children or the general population and this should be the subject of a separate systematic review. Further, we aimed to infer our results to the general population. We revised the text in the Methods section to better justify these exclusions: *“Studies in children with rare or special conditions, such as congenital heart diseases, or postnatal conditions conferring an increased risk of CVD, such as hypertension or diabetes, were not considered for inclusion. We aimed to make inferences to the general population. Further, we assumed that there may be a different effect of DOHAD exposures in these children than in the general population, which should be evaluated separately”.* Of note, the necessity of restricting the focus of the review was documented through searches and study screening based on the large eligibility criteria in our published study protocol (Epure et al., 2018), which resulted in 1,159 abstracts selected for full-text screening. This finding about the extensive existing research on CIMT was presented at a regional scientific meeting in Switzerland (Conference abstract 1, *Summary of research output related to the study* submitted for peer-review purposes). However, this tremendous workload was not anticipated at the protocol stage and it became apparent that a restriction in focus was required to maintain the project feasible and intelligible, but that didn’t affect its broad scope and value. This decision is not uncommon for systematic reviews, as highlighted in the PRISMA guidelines: “the PICOS [participants, interventions, comparators, outcomes, and study design] criteria should be formulated a priori, in the systematic review’s protocol, although some revisions might be required due to the iterative nature of the review process.” (Liberati et al., 2009).

4. *Comment:* *Childhood is per se not defined as 0-18y, but as the age span ranging from birth to adolescence.* *On the other hand, although the study states that they included children aged 0-18. Table 3 shows that a majority of the study subjects included belong to the younger age groups (36/50 studies report age less than 9y), so the focus is on childhood. However, the use of the term childhood should be consistent with common definitions.*

- *Please check this the use of this terminology throughout manuscript* *including title, abstract, manuscript text, figure and tables, supplements.*

**Response:** We thank Dr Sarkola for pointing this out. We replaced “childhood” with “infants, children, and adolescents” throughout the manuscript.

5. *Comment: Comparators. No eligibility criteria is provided although this seems key in the comparison. Published study protocol states: "Studies assessing specific clinical conditions in relationship with CIMT will be included provided they use a control group without the clinical condition of interest". Age, body size, puberty, CVR-profile including BP, race etc. are well known important key predictors of CIMT in children. Do the authors mean to say that no requirements for comparator groups needs to be specified in order to assess differences in CIMT in relation to DOHaD variables? Differences in vascular dimensions reported in the different studies should be adjusted for differences in key covariates. This is not clearly presented in Data Analysis. Please do elaborate and revise accordingly.*

**Response:** The comparator item in the Eligibility criteria refers to what specific characteristics (if any) the comparison group for the exposure of interest should have. As exposures that were originally measured and analyzed as continuous or categorical variables were both equally eligible for inclusion, we did not have any specific requirements for the comparator in order for a study to meet the eligibility criteria for this systematic review, as pointed out by Dr. Sarkola too. Therefore, where applicable, the use of a reference group without the exposure of interest was sufficient for inclusion (*yes versus no; exposed versus reference*). Regarding covariates, we clarified our approach for pooling results across studies in the Data analysis section and provide a thorough explanation below at comment no. 9. We revised the text in the Methods section: *“Where applicable, the use of a reference group without the exposure or intervention of interest was sufficient for inclusion”*.

6. *Comment: Exposures/interventions. "For observational studies, we considered for inclusion prenatal and postnatal exposures, at the individual, familial, or environmental level, which reflected the developmental milieu of the child and occurred between conception and age 24 months" This relates to the DOHaD Figure 1 states that 20 articles were excluded due to missing exposure or intervention of interest. Please specify in more clear terms what prenatal and postnatal exposures were considered here? Birth weight, gestational age at birth, maternal disease, gestational disease or complication, infections, medications etc. Examples could be provided.*

**Response:** We revised the text as follows: “*We considered for inclusion prenatal and postnatal exposures, at the individual, familial, or environmental level, which reflected the developmental milieu of the child and occurred between conception and age 24 months (i.e. first 1000 days of life).* *These included biological factors related to physiology, physiopathology, or epigenetics, and contextual factors related to socioeconomic status, behaviors, or environmental toxins. The selection of exposures was informed by the review of Hanson and Gluckman (Hanson and Gluckman, 2014) and Moore et al (Moore et al., 2017) and was conditioned by the timing of occurrence or ascertainment of exposure, as appropriate.”*

7. *Comment: Disagreement between reviewers were solved by discussion. Could this be presented with numbers somehow?*

**Response:** The mean proportionate agreement between reviewers at the title and abstract screening was 94% and at the full-text screening was 84%. This information was added in the Methods section (Study selection process).

8. *Comment: Data extraction. Quality of study assessment. Supplemental Table 3. Criteria for study quality and study reliability tool criteria and algorithm of judgement seems arbitrary and poorly justified. Why is at least 2 images/frames needed in addition to manual tracings to indicate improved reliability? How does assessing more frames improve CIMT measurement reliability? No data regarding this is presented. Intra and interobserver variabilities are commonly performed in standardized vascular laboratories as quality assurance measures, and may not always be reported in articles methods section. The non-reporting of these (or only referring to a reference) is then by no means evidence of a lower technical operator performance or lower measurement reproducibility. There other more important factors related with this that could be included: standardized imaging and measurement protocols, operator experience and skill, image quality, ultrasound equipment and transducer frequency, imaging feasibility (child preparation with measurements performed at rest; sedation as appropriate), site of carotid intima media thickness, just to mention a few. Reading Box 2 in the published study protocol in BMJ Open suggests a different approach for this assessment. Please justify the choice of different factors included in this manuscript Supplemental table 3. Moreover, "The quality of each study was evaluated using an adjusted version of the Newcastle-Ottawa Scale for observational studies and Cochrane's collaboration risk of bias tool for experimental studies." How this was done remain largely unclear. This also applies to the information provided in Supplemental Table S7, Supplementary Figure 1S. How was study quality data generated? What defines a lo quality study and a high quality study addressed in subanalyses and mentioned in the discussion? The study quality assessment should be clearly presented and outlined.*

**Response:** For study quality assessments, we provided in the supplementary file the definitions and judgement criteria for the bias domains of the Newcastle-Ottawa Scale and Cochrane's collaboration risk of bias tool (Supplemental Table S7). We revised the legend in Supplemental Table S~~7~~8 to clarify that *high* means high quality and low risk of bias, whereas *low* means low quality and high risk of bias. As requested by reviewer 3, we added Supplemental Table S9, which provides individual quality assessments for each study included in meta-analyses. For CIMT reliability assessments, we used the same tool and algorithm of judgement as the one provided in the supplementary file of the peer-reviewed and published study protocol (Epure et al, 2018). The non-reporting of items necessary for CIMT reliability assessment was classified as “unclear” because of insufficient information to permit judgement. Therefore, these studies were rated as unclear reliability, not as lower reliability.

9. *Comment: Data analyses. The SMD measure adjusts for methods differences in scale and precision. However,* *there may be differences between the exposure and comparator that are explained by covariates such as body size (weight, lean body mass or head circumference) that is not accounted for in this meta-analysis metric. The main stated results relate to being born small for gestational age. How differences in body size between exposure and comparator (reference) groups was taken into account in the generation of the SMD and this conclusion? How does measurement precision affect SMD (influence of the SD on the SMD metric?) and how does this influence the reported outcome SMD and conclusions thereof?*

**Response:** We meta-analyzed bivariate effect sizes in all meta-analyses, except for maternal smoking during pregnancy, which included two bivariate effect sizes (Ayer (2011), Geerts (2012)) and one partial effect size (Gale (2006)). For the latter, we pooled together bivariate and partial effect sizes because the partial effect size reported in the study of Gale (2006) was least adjusted for sex and its magnitude was comparable to the bivariate effect size reported in the study of Ayer (2011). While adjusting for extraneous factors is unquestionably important when attempting to make causal inferences, with this systematic review we are in the framework of prediction or correlational data, summarized across studies using aggregate data meta-analyses (AD-MA). One of the main limitations of AD-MA is the access mainly to summary data published in study reports. Another limitation is that the meta-analyst relies on the analysis strategy of the original study investigators, with some studies only providing bivariate effect sizes, i.e. two-variable associations (Lipsey and Wilson, 2000), and others only partial effect sizes, i.e., the association of two variables that has been statistically adjusted for the effect of one or more covariates (Aloe, 2015). Additionally, it is very common that observational studies report partial effect sizes adjusted for different number and types of covariates, which can give rise to effect sizes that are smaller, larger, or, in some cases, even in opposite direction compared to the bivariate relationship. Therefore, combining bivariate and partial effect sizes in the same AD-MA can be misleading with respect to both the magnitude of the association and the variability among effects sizes (Aloe 2105). Such an approach is generally not recommended and may be implemented only if bivariate and partial effect sizes are conceptually and statistically comparable (Aloe, 2015), as it was the case for our meta-analysis of maternal smoking. We clarified in the Data Analysis section the approach that we used for pooling data by replacing the phrasing “*The unadjusted or least adjusted association estimates were used to pool data across studies”* with the following: “*Estimates from bivariate associations or partial associations that were least adjusted for sex were used for pooling data across studies (Aloe, 2015).”* We acknowledged in the Discussion section that the use of unadjusted or least adjusted data to pool results across studies is a limitation of the review. Moreover, where applicable, data on covariates included in partial associations are provided in Supplemental Table S5.

The measurement error increases the variance, and, hence, the standard deviation of the outcome variable, which enters the denominator of the standardized mean difference. Therefore, effect sizes are likely to be smaller in the presence of measurement error. To overcome this limitation, we performed sensitivity analyses based on studies with higher CIMT reliability that resulted in larger pooled estimates, consistent with the reasoning above. Additionally, as the amount of measurement error increases, differences between exposure groups may become more and more obscure, and, therefore, difficult to detect by the conventional significance tests. This is why, when interpreting results, we focused on the point estimates and the uncertainty around them.

10. *Comment: The reported I2 50-83% (child level) in conjunction with the SMD in Table 3 indicate a high heterogeneity and inconsistency between the studies included in the meta-analysis (similarly high in Table 4 subgroup analyses). Could the high heterogeneity be explained by methodological differences between the studies? Could this be explored in more detail. Moreover, how does the high heterogeneity between the studies impact on the conclusion of a higher CIMT among children born small for gestational age? Please explain the use of I2 and tau2 metrics of heterogeneity in Data Analysis and, in particular, include interpretations on these as well. In addition, please elaborate on this in the manuscript discussion.*

**Response:** I^2^ and tau^2^ are standard measures of heterogeneity in random-effects meta-analysis (Borenstein et al., 2009) that reflect the amount of true heterogeneity between studies, i.e., the variation in effect sizes that is not due to sampling error. Tau^2^ is the variance of the true effects, on the same scale as the effects themselves. This value is used to assign study weights under the random-effects model. I^2^ is the percentage of variation in effect sizes attributable to heterogeneity, ranging from 0% to 100%, with 0% indicating that all variation in effect sizes is due to sampling error within studies and 100% indicating that all variation is due to true heterogeneity between studies. We included this additional information in the Methods section. Regarding the impact of the high heterogeneity on the conclusions, it was expected that the review will include data from studies that have differences in design, population or exposures, which are likely to result in different effects or associations across studies. It was, thus, a priori decided to perform meta-analyses with a random-effects model to take into account the high between-studies heterogeneity. The pooled point estimates have wider standard errors and confidence intervals under the random-effects model, impacting the precision and the overall certainty of the results. We performed subgroup-analyses based on the sources of heterogeneity defined in our study protocol. We explored the methodological differences based on the CIMT quality (reliability) analyses. We did not run other statistical analyses due to the relatively low number of studies.

11. *Comment: Results. Table 4, footnote. "P-value reported for between group heterogeneity". What groups? Is this p-value in conjunction with I2 and tau2 metrics (similar to table 3) between small vs appropriate birth size (weight?) or is it some kind of comparison of the heterogeneity related with subgroup factor such as transducer frequency or age? Please clarify.*

**Response:** Random-effects models were used for pooling studies within each subgroup of the variables in Table 4, for example, sample size or CIMT wall. Therefore, I^2^ and tau^2^ are measures of heterogeneity within each subgroup. The p-value is given by a standard test for heterogeneity across subgroup results (Cochran Q) that tests for the variation in mean effect sizes in the different subgroups (Borenstein et al., 2009, Higgins and Green, 2011). A low p-value provides evidence for variation in effect sizes beyond chance (Higgins and Green, 2011). Relevant references explaining in detail these heterogeneity statistics were provided in the Methods section. We clarified in the footnote of Table 4 that “*I^2^ and tau^2^ assess within-group heterogeneity*; *p-value assesses between-group heterogeneity”*.

12. *Comment: Table 4 shows that the higher CIMT in children born small for gestational age (SMD) disappears when assessing studies using higher ultrasound frequencies. This finding should be highlighted, as the measurement accuracy is likely to be much better younger populations with thinner CIMTs.*

**Response:** As Dr. Sarkola pointed out, studies that used transducer frequencies > 12 MHz had a mean pooled effect size that was lower compared to the those that used < 12 MHz. We highlighted this finding in the Discussion section, paragraph on CIMT: “*We could notice large decreases in the magnitude of association estimates across studies using higher transducer frequencies.”*

13. *Comment: Exposure to maternal smoking had a consistent association with higher CIMT (SMD 0.12, CI95% -0.06-0.30), is this statistically significant? Is this mediated by the effect of gestational smoking on birth weight?*

**Response:** Due to the fact that we assessed multiple exposure types in different studies, with some potentially underpowered to detect a significant association, we focused on the effect size and the uncertainty around it, and not on the level of significance or if the p-value reached a given threshold. We clarified in the methods section how results were interpreted: “*As recommended by Amrhein et al (Amrhein et al., 2019), together with more than 800 co-signatories of the initiative "Retire statistical significance", and because we assessed multiple associations, we interpreted the point estimate as the most compatible value of the observed association and did not focus on the level of statistical significance. The uncertainty around the point estimate is comprised in the accompanying 95% confidence interval.”*

We don’t have the data to address the mediation effect of birth weight on the association of gestational smoking with CIMT. As explained above, to pool data across studies, *we used estimates from bivariate associations or partial associations that were least adjusted for sex*. In this systematic review we are in the framework of correlational data, summarized across studies using aggregate data meta-analyses (AD-MA). However, adjusting for relevant covariates is unquestionably important when drawing causal inferences and the selection of covariates is relative to the research question and the causal effect of interest. Assuming an explicit causal reasoning, conditioning on birth weight, which is considered to be an intermediate on the causal pathway from smoking to the CVD outcomes, including CIMT, is indeed useful to separate the direct effect of the exposure on the outcome from the indirect effect (i.e., the effect of the exposure that operates through the intermediate), but this is a fundamentally different question that could be appropriately answered by an individual patient data meta- analysis.

14. *Comment: Figure 2. Small size for gestational age. What was the criteria for SGA applied? What was the age of the population at CIMT assessment? Are these newborns or children of higher age? What is the effect of postnatal growth? Are the exposure and reference groups comparable with respect to age and body size?*

**Response:** The definition of small size for gestational age has been provided in the main text

as a footnote to *Table 3. Summary of findings for each exposure type included in meta-analyses*. The age at CIMT assessment spans from the newborn period to early adolescence (16 years). It has been provided for each study in *Table 1. Characteristics of included studies*. We revised the description of the association of smoking with CIMT in Results, as follows: “*Children […]* *exposed to maternal smoking during pregnancy had, on average, an increased CIMT, but the* *imprecision around the estimates was high.”* We don’t have the data to address the mediation effect of postnatal growth on the association of small size for gestational age with CIMT, as explained above for the association of smoking with CIMT (comment no. 13). With respect to age and body size, please see our response at comment no. 9.

15. *Comment: What is number in brackets after first author name in Supplemental Table S4?*

**Response:** The number in brackets is the reference number according to PLOS Medicine’s in-text citation style.

16. *Comment: Supplemental Table 5. Birth size (weight). How comparable are the studies concerning birth size? Has the mean difference been adjusted for body size? Covariates? Is the CIMT in accordance with body size (lean body mass or HC size)?*

**Response:** We are not sure to understand what Dr Sarkola refers to: comparability of studies in terms of what? No covariates were included in the analysis, unless specified in the covariates field in Supplemental Table S5. For full explanation on covariates, please check our response to comment no. 9. CIMT was measured in mm, thus, it was not normalized according to body size.

17. *Comment: Discussion. Discussion. … followed by ART and maternal smoking during pregnancy consistent effect on CIMT? Was the effect of ART statistically significant? Reported as a tendency in results (SMD 0.78, CI95% -0.20 to 1.75).*

**Response:** Please see our response above with regard to the statistical significance of smoking. We revised the text on ART in results as follows: “*Children conceived through ART […] had, on average, an increased CIMT, but the imprecision around the estimates was high.”.*

18. *Comment: Please address CIMT associations with race and sex, and body composition (e.g. adiposity)?*

**Response:** We presented data on body composition and CIMT in the Supplemental table S5. Data from studies reporting the association of CIMT with DOHAD factors separately for males and females is also presented in Supplemental Table S5 (e.g. Crispi et al (2010), Gruszfeld et al (2015)). There were no studies reporting these associations in subgroups defined based on race.

19. *Comment: What does the SMD 0.40 correspond to in mm? What is the clinical relevance of this from a CVR perspective?*

**Response:** A SMD (95% CI) of 0.40 (0.15, 0.64) would correspond to a CIMT that is 0.02 (0.01, 0.03) mm higher in the SGA compared with reference. The computation was performed assuming a standard deviation (SD) of 0.44 mm, which was estimated based on CIMT data obtained with a 12-MHz linear-array transducer in a sample of 204 healthy children between 8 and 18 years (Torigoe et al., 2020). Our estimate of a higher CIMT among SGA children may be clinically important as it is comparable to that detected for postnatal clinical conditions considered at a high risk of CVD, such as type 1 diabetes. In fact, a recent systematic review with meta-analysis detected a CIMT that was 0.03 mm (95% CI: 0.02, 0.04) higher in children and adolescents with type 1 diabetes (mean age 15 years; mean disease duration 7 years) compared with reference (Giannopoulou et al., 2019). However, the transformation to mm should be interpreted cautiously because it uses the SD of a single study, whereas the pooled estimate was based on multiples studies. Due to this limitation in data transformation to mm and to keep the discussion short, we preferred not to include the equivalent in mm or discuss it in the present manuscript. However, please see our paragraph on prevention of SGA in the Discussion section (Implications for research and practice).

20. *Comment: Overall, the discussion is too long and speculative.*
a) *Please focus on discussing in relation to objective.*
b) *The recommendations for CIMT measurements presented in the conclusions are not warranted by the results, they are too strong and very subjective (e.g. no data provided suggesting superiority of border detection software in pediatric CIMT or the analysis of 2 image frames using calipers), the line of reasoning is missing here.*

**Response:** As proposed by the reviewer, we have shortened some parts of the discussion. We have deleted the recommendations on CIMT measurement.

21. *Comment: Minor comment from reviewer related with CIMT methodology. The authors might be interested in studies addressing non-invasive ultrasound-derived pediatric (and adult) arterial layer method validation using histology (Sarkola et al Atherosclerosis. 2010 Oct;212(2):516-23; Sarkola et al Atherosclerosis. 2012 Sep;224(1):102-7; Sundholm Atherosclerosis. 2015 Apr;239(2):523-7; Sundholm et al Ultrasound Med Biol. 2019 Aug;45(8):2010-2018). These studies illustrate important caveats related with the ultrasound CIMT measurement during infancy/childhood. In brief, these are related with 1) the true histological thinness of the arterial wall layers during infancy/childhood exemplified in the present review by references reporting ultrasound IMTs in infants (e.g.Trevisanuto et al 2010, Schubert et al 2013, Sodhi et al 2015, Chen et al 2019) similar to commonly reported in young adult age (e.g. Engelen et al* [*https://doi.org/10.1093/eurheartj/ehs380*](https://doi.org/10.1093/eurheartj/ehs380)*), and references reporting IMTs childhood (e.g. multiple studies by Ayer and Skilton, Ciccone et al 2016, Valenzuela-Alcaraz et al 2019) similar to commonly reported in older adult age which is biologically impossible, 2) frequency dependence of ultrasound axial resolution (i.e. lower frequency providing higher IMT) and ultrasound frequency effect on IMT measurement accuracy (conventional frequency axial resolution limit >0.25 mm higher than infancy/childhood IMT <0.25). I hope that these could guide the authors in their revision.*

**Response:** As suggested, we have added these very interesting papers and made some brief comments in the Discussion section.

*“In our case, the sensitivity analyses performed for small size for gestational age and prematurity showed that the quality of CIMT measurement methods had a significant impact on the results. Additionally, CIMT values may differ with the edge detection method and the specificities of the ultrasound equipment used, such as the transducer frequency.* *We could notice large decreases in the magnitude of association estimates across studies using higher transducer frequencies. Our finding is in line with other evidence showing that conventional high frequencies (≤ 12 MHz) may have insufficient ultrasound resolution and result in an overestimation of the true thickness in children under 12 years of age (Sarkola et al., 2010, Sarkola et al., 2012). Novel techniques that measure CIMT with very-high resolution ultrasound (25 to 55MHz) seem to be more precise in infants and young children and at very early stages of atherosclerosis (Sundholm et al., 2015, Sundholm et al., 2019).”*

**REVIEWER #3: Name not provided.**

GENERAL REMARKS

*This meta-analysis summarizes the relevance of perinatal life factors for carotid-intima thickness in children and adolescents. In view of the fact that the first 1000 days are regarded as a potentially critical period for shaping later cardiometabolic risk this is a topic of substantial public health relevance. In addition, evidence from several studies suggests that associations of factors acting in this window of opportunity may already impact carotid intima-media thickness, i.e. an established marker of subclinical atherosclerosis.*

*Nonetheless, a number of issues arise with this manuscript*

POINTS OF DETAIL

*Major issues*

1. *Comment: Main results. Only one of the three "main" results (born small for gestational age) is statistically significant. This is not correctly presented and discussed by the authors.*

**Response:** Due to the fact that we assessed multiple exposure types in different studies, with some potentially underpowered to detect a significant association, we focused on the effect size and the uncertainty around it, and not on the level of significance or if the p-value reached a given threshold. We clarified in the methods section how results were interpreted: “*As recommended by Amrhein et al (Amrhein et al., 2019), together with more than 800 co-signatories of the initiative "Retire statistical significance", and because we assessed multiple associations, we interpreted the point estimate as the most compatible value of the observed association and did not focus on the level of statistical significance. The uncertainty around the point estimate is comprised in the accompanying 95% confidence interval.”*

2. *Comment: Aim 2:*

- *The necessity for cIMT method appraisal remains elusive. I do not question them, but they should be explained in the introduction. Why should the between-study heterogeneity matter? What are the main problems associated with this and could this affect the associations under investigation?*
- *The authors provide very little information on their cIMT measurement quality assessment.*
- *Generally, the term "reliability" appears incorrect. Many of the aspects assessed could also affect the validity of the measurements.*

**Response:** We thank the reviewer for their suggestion to better justify the necessity of the CIMT method appraisal and we revised our Introduction section accordingly. We did so by allocating a full paragraph (3^rd^) to describe the CIMT measurement issues. The CIMT measurement quality for this systematic review was evaluated according to a pre-defined tool, published in 2018 (Epure et al., 2018), that was developed according to guidelines and standards in the field (Dalla Pozza et al., 2015, Urbina et al., 2009, Stein et al., 2008, Bots et al., 2003, Touboul et al., 2012, Tang et al., 2005). The criteria and algorithm of judgement were presented in *Supplemental Table S3. Criteria for CIMT quality assessments (CIMT Reliability Tool and algorithm of judgement)*. This tool evaluates the variability in CIMT measurement associated with the (1) site of measurement, (2) image analysis methods and (3) reproducibility of measurements. For example, it was shown that the measurements taken from the common carotid artery far wall, or analyzed through automatic edge detection methods, are less susceptible to inter-observer variability (Dalla Pozza et al., 2015, Urbina et al., 2009). We, therefore, consider that the 3 domains of the CIMT quality measurement tool rather concern reliability matters than validity ones. Moreover, the ultrasound CIMT measurement has been validated against histology and for the assessment of atherosclerotic changes (Pignoli et al., 1986, Wong et al., 1993, Amato et al., 2007).

3. *Comment: Intervention studies:*

- *These can only be considered as such if the primary endpoint of the study was cIMT. This was only the case for the study of Ayer et al. (Ref. 54) The second study had body composition as a major outcome. Hence, the post-hoc analysis of this study for an association with later cIMT should be regarded as an observational cohort study.*
- *The study of Ayer et al. (Ref. 54) is mentioned both as a cohort and an intervention study. Please, clarify.*

**Response:** We thank the reviewer for raising this point and we acknowledge that, compared to primary analyses of interventional studies, post-hoc analyses are susceptible to a higher risk of bias, especially due to selective reporting. We took this in consideration in our quality assessments- selective reporting item, where the study of Gruszfeld et al was rated as low quality. However, we consider that the study of Gruszfeld et al can be regarded as interventional because the intervention was assigned by the study investigators and participants were followed up prospectively to assess differences in outcomes, including those specified at a later time point, such as CIMT. With respect to the study of Ayer et al, a note in the footnote of Table 1 explains why it was mentioned both as a cohort and as an intervention study: *“Note: Studies reporting on both interventions and exposures, were analyzed as randomized controlled trial for the data on interventions and as observational cohort for the data on exposures (N=2, namely Ayer [54], 2009 (b) and Gruszfeld [72], 2015).”*.

4. *Comment:* *Exposures*

- *it remains elusive how the authors arrived at their set of exposures. Were there any a priori criteria (they were not part of the search strategy)? Were all exposures analysed in a relevant publication included?*
- *Many exposures could have multiple indicators, e.g. duration of breastfeeding would certainly be reported more specifically (exclusive vs full vs any), yet it is not clear how the authors attempted to address/combine these.*
- *A table on all early life risk factors and their "definitions" would be helpful, e.g. clarify "diet and feeding practices"*
- *Some of the exposures appear questionable: epigenetic changes, cortisol and cardiometabolic factors in childhood are presumably markers of mechanisms by which perinatal risk factors could affect the cardiometabolic outcome cIMT rather than exposures*

**Response:** We used “exposure” as a general term to include *“biological factors related to physiology, physiopathology, or epigenetics, and contextual factors related to socioeconomic status, behaviors, or environmental toxins. The selection of exposures was informed by the reviews of Hanson and Gluckman (Hanson and Gluckman, 2014) and of Moore et al (Moore et al., 2017) and was conditioned by the timing of occurrence or ascertainment of the exposure, as appropriate.”* We added this information in the Methods section (Eligibility criteria Exposures/Interventions subheading). We justified in the Methods section how categorical exposure variables were addressed: “*(2) if a study reported on multiple exposed or reference subgroups, they were combined into one using formulae provided by the Cochrane collaboration in case of means and standard deviations (Higgins and Green, 2011) or fixed-effect meta-analysis in case of association estimates for each subgroup (Borenstein et al., 2009).”* We provided *Supplemental Table S5.* that includes the main risk factors at each level and category, their comparison levels and definitions.

5. *Comment: Age at outcome. One of the main limitations is the large variability in the age at outcome (0-18 years). This is hardly mentioned and the sensitivity analysis only distinguishes 0-1 from 2-18 years.*

**Response:** We agree and acknowledged the high variability in age as a limitation in the Discussion section. We further added: *“**The variability in age at CIMT assessment was large, from 0 to 18 years. Our subgroup analysis was mainly based on the age range reported in each study and, thus, could only distinguish the infants from the other pediatric age groups combined.”*

6. *Comment: Search. Were searches performed in duplicate?*

**Response:** The queries for the systematic searches were developed in collaboration with 2 experienced librarians (*Acknowledgements* section) and then run in the databases by a single reviewer. The study selection was performed in duplicate by 2 independent reviewers (*Study selection process* heading).

7. *Comment: Risk of bias / Grading of evidence*

- *The authors use the Newcastle Ottawa scale for observational studies, but do not report findings per study. They only provide a summary figure by exposure, i.e. the reader cannot confirm their judgement. Some aspects in this figure would need explanation (i.e. "same method of ascertainment or "comparability")*
- *The Assessment of study quality for interventions studies given in Supplementary Table 7 is not referred to in the text and the table is not intelligible. Does "high" indicate high quality or high risk of bias?*
- *The quality of meta-evidence needs to be graded*

**Response:** For study quality assessments, we provided in the supplementary file the definitions and judgement criteria for the bias domains of the Newcastle-Ottawa Scale and Cochrane's collaboration risk of bias tool (Table S7). We revised the legend in Supplemental Table S~~7~~8 to clarify that *high* means high quality and low risk of bias, whereas *low* means low quality and high risk of bias. As requested, we added Supplemental Table S9, which provides individual quality assessments for each study included in meta-analyses. Regarding the certainty (quality) of the meta-evidence, we added in the Discussion section, paragraph on limitations: “*The main limitation is that the body of evidence for this systematic review is largely observational, which is of low certainty by design […]*”. This statement was followed by a detailed discussion on specific limitations, with the main ones also listed in the Abstract- last sentence of the *Methods and Findings* section: “*The degree of confidence in results is limited by the low number of studies with relatively small sample sizes and the high between-study heterogeneity.*”

8. *Comment: Discussion of the findings*

- *An appraisal of the "effect sizes" and their public health relevance is missing*
- *Heterogeneity due to the age-range at outcome measurement (only first year vs 2-18 yrs) longitudinal vs cross-sectional needs to be discussed*
- *Lack in focus: The authors discuss a number of potential mechanisms driving their observations, even for small sensitivity analyses for which data are not shown (and are perhaps not significant). In view of this reviewer, this should be focusing on the main findings of the meta-analysis only.*

**Response**: As requested, we shortened the discussion and focused on the main result, that is, small size for gestational age. We discussed its public health importance in the light of the prevalence of the risk factor and its potential as a target exposure for primordial prevention of cardiovascular disease. With respect to the heterogeneity due to age, please see our response at comment no. 5 above. We mentioned the variability due to study design as a limitation of the review, but to keep the discussion short, we did not further expand on it.

*“…* *our study indicated that children which were small for gestational age had a higher CIMT in early life. Prevention of small for gestational age could have a big impact from a public health perspective. Its prevalence varies among populations and raises with decreasing gestational age and availability of resources, reaching about 20% in low and middle-income countries (Lee et al., 2017). Moreover, modifiable factors, including gestational smoking or poor maternal nutrition, account for a large share of small for gestational age infants (Wollmann, 1998). In fact, smoking cessation interventions during pregnancy were shown to result in a 17% reduction in low birth weight cases (Chamberlain et al., 2017). Public health strategies to reduce smoking and improve the nutritional status in women of reproductive age should be advocated and may improve the cardiovascular health too (Chamberlain et al., 2017, Barker et al., 2018).”*

*Minor issues*

9. *Comment: Abstract: CIMT methods cannot be appraised with a meta-analysis*

**Response:** We agree that the CIMT methods cannot be appraised with a meta-analysis. However, this is a systematic review with meta-analyses, thus, the systematic review process meets our objective by identifying studies that measured CIMT, extracting data on their measurement methods, and summarizing their findings descriptively in the Results section: paragraph on CIMT measurement methods, Table 2, Supplemental Table S4. The summary of evidence on CIMT measurement methods was also critically discussed in the Discussion section. To improve clarity, we placed *“a systematic review with meta-analysis”* at the beginning of the last sentence in the Background sections of the abstract and the main text (Objectives subheading).

10. *Comment: Number for observational studies should be given separately for cross-sectional and longitudinal studies*

**Response:** We provided this information in the Results section (Study characteristics subheading).

11. *Comment: Why was this study done: "It is unclear whether …" is not a convincing argument to perform such a study. Preliminary (mechanistic) evidence suggesting that this could be relevant should be given.*

**Response:** We agree with the reviewer. We removed that sentence from the abstract. In the author summary, *Why was this study done* section, we replaced it with: *“Exposure to adverse experiences in the first 1000 days of life, that is, from conception to age 2 years, may determine adaptative changes in the vascular morphology and increased carotid intima-media thickness in infants, children, and adolescents”.* We provide extensive justification for our objectives in the Background subheading of the main text.

12. *Comment: Funding information should not be mentioned in methods and findings*

**Response:** We agree with the reviewer and we revised accordingly.

13. *Comment: What does the age range in the abstract refer to? Age at cIMT measurement?*

**Response:** We clarified that the age provided in the abstract’s Methods and Findings represents the age at CIMT assessment: *0 to 18 years at CIMT assessment*.

14. *Comment: The authors should already introduce the concept of perinatal programming and the mechanisms discussed in this context in the background. Why should we expect that early life factors could already affect cIMT in childhood/adolescence?*

**Response:** We thank the reviewer for this suggestion and we revised accordingly in the 2^nd^ paragraph of our introduction: *Within a DOHaD perspective, these associations may be explained by early life adaptative changes in the vascular phenotype (Visentin et al., 2014), with lifelong effects on the CVD risk.*

15. *Comment: The conclusion on CIMT method "cIMT measurement protocols are heterogeneous and often poorly reported" is vague. What does this refer to? Validity or reliability of the method? Lack of data on intra- and inter-observer variability? Potential for investigator-associated bias (unstandardized measurement procedure)?*

**Response:** This statement is based on the results summarized under the subheading CIMT measurement methods (Results section) and detailed in Table 2 and Supplemental Table S4. It was further explained and developed in the paragraph discussing the limitations of the CIMT measurement (Discussion section). Our assessments based on the CIMT quality tool mainly reflect the reliability of the method. Please also see our response to comment no.2 above.

16. *Comment: Purpose of a meta-analysis. In the discussion the authors state that their review is needed to better understand DOHaD mechanisms. In the view of this reviewer a meta-analysis based primarily on observational studies can only provide suggestions for potential mechanisms which need to be addressed in other subsequent studies. Instead, quantification of associations, evidence on public health relevance etc. are most likely more important contributions of a meta-analysis.*

**Response:** We agree. We revised that statement in the Discussion as follows: *“Assessing the relationship* *with carotid remodeling already in children was needed to shed light on the consistency and magnitude of associations, their clinical and public health importance, as well as to provide suggestions for mechanisms that need to be addressed in subsequent high-quality studies.”*

17. *Comment: Experimental studies should be termed intervention studies*

**Response:** We thank the reviewer for this suggestion and we revised accordingly.

18. *Comment: Participants cannot be recruited pre-birth, whereas their mothers can*

**Response:** We stated that participants can be recruited pre-birth: *“We considered for inclusion studies in children from birth up to 18 years old, including studies where participants were recruited pre-birth.”* (Eligibility criteria, Participants subheading).

19. *Comment: Please check language, e.g. lines 148-150, does not appear to be a sentence*

**Response:** We thank the reviewer for this suggestion and we revised accordingly.

20. *Comment: Results: When referring to specific studies the reference should be given*

**Response:** We thank the reviewer for this suggestion and we revised accordingly.

21. *Comment: Line 293: this relation is no longer significant!*

**Response:** Please see our response to comment no. 1.

22. *Comment: Strength: performing a meta-analysis according to a protocol is not a strength.*

**Response:** We adhere to the PRISMA guidelines for reporting systematic reviews and meta-analyses which highlight that making a protocol available to readers, through its registration or publication, is important because “it pre-specifies the objectives and methods of the systematic review, “can help restrict the likelihood of biased post hoc decisions in review methods, such as selective outcome reporting.” (Liberati et al., 2009). Following such a procedure is considered a strength in the field of systematic review and meta-analyses.

23. *Comment: How was the association between study quality (RoB) and method quality?*

**Response**: Our study was not designed to quantify such a correlation. For a broad view on the relationship between CIMT quality assessments and study quality assessments, we present both types of assessments, for each study, in Supplemental Tables S8 and S9.

24. *Comment: Strength of relationships (e.g. line 301) are an interpretation of the results, not a result per se.*

**Response:** We were referring to the magnitude of the association. We replaced the term “strength” with “magnitude” in the Results section.

25. *Comment: Fixed-effects meta-analysis was used for data simplification, yet random-effects meta-analysis for the main analysis. Please explain.*

**Response:** This is because: 1) we used the study as the unit of analysis, 2) it was assumed the effect sizes to be relatively consistent within a study, but to very substantially from one study to the other (Borenstein et al., 2009, Higgins and Green, 2011). To allow for this between-study variation, we used a random-effects model only across studies. We provided in the Methods section the reference of Borenstein et al., 2009 that discusses in detail this approach.

26. *Comment: The discussion of the breastfeeding non-finding is misleading and should be shortened. The findings in this meta-analysis do not permit any public health statements relating to breastfeeding.*

**Response:** We removed the paragraph on breastfeeding from the Discussion section. This is in line with the second reviewer’s request about focusing the discussion on the main results.

**Table 4.** Subgroup meta-analyses of standardized mean differences (SMD) for the association of birth size for gestational age or gestational age with carotid intima-media thickness.

|  | **Birth size for gestational age (small versus appropriate)** | | | | **Gestational age (preterm versus term)** | | | |
| --- | --- | --- | --- | --- | --- | --- | --- | --- |
|  | **N studies** | **SMD (95%CI)** | **I^2^, tau^2^** | **P** | **N studies** | **SMD (95%CI)** | **I^2^, tau^2^** | **p** |
| **All** | 16 | 0.40 (0.15 to 0.64) | 83%, 0.18 | - | 7 | 0.03 (-0.17 to 0.22) | 50%, 0.03 | N/A |
| **Study design** |  |  |  |  |  |  |  |  |
| Cohort | 7 | 0.39 (0 to 0.78) | 79%, 0.2 | 0.528 | 5 | 0.1 (-0.13 to 0.33) | 45%, 0.03 | 0.030 |
| Cross-sectional | 9 | 0.41 (0.07 to 0.74) | 87%, 0.21 |  | 2 | -0.18 (-0.39 to 0.04) | 0%, 0 |  |
| **Study setting** |  |  |  |  |  |  |  |  |
| Community or population-based | 1 | -0.09 (-0.30 to 0.13) | - | <0.001 | 3 | 0.08 (-0.23 to 0.38) | 73%, 0.05 | 0.716 |
| Health-care facility | 13 | 0.43 (0.14 to 0.72) | 83%, 0.21 |  | 4 | -0.04 (-0.34 to 0.27) | 32%, 0.03 |  |
| N/S | 2 | 0.60 (0.36 to 0.85) | 1%, 0.00 |  | - | - | - |  |
| **CIMT wall** |  |  |  |  |  |  |  |  |
| Far | 11 | 0.38 (0.11 to 0.64) | 82%, 0.15 | 0.756 | 6 | 0.08 (-0.11 to 0.27) | 42%, 0.02 | 0.066 |
| N/S | 5 | 0.47 (-0.17 to 1.12) | 88%, 0.46 |  | 1 | -0.37 (-0.81 to 0.06) | - |  |
| **CIMT sides** |  |  |  |  |  |  |  |  |
| Left and right | 12 | 0.5 (0.22 to 0.79) | 84%, 0.19 | 0.196 | 4 | 0.02 (-0.2 to 0.23) | 41%, 0.02 | 0.059 |
| Right | 2 | 0 (-0.35 to 0.35) | 0%, 0.00 |  | 1 | 0.35 (0.01 to 0.69) | - |  |
| N/S | 2 | 0.11 (-1.00 to 1.21) | 95%, 0.60 |  | 2 | -0.22 (-0.6 to 0.17) | 19%, 0.02 |  |
| **CIMT edge detection method** |  |  |  |  |  |  |  |  |
| Automatic or semi-automatic | 4 | 0.35 (0.04 to 0.67) | 69%, 0.06 | <0.001 | 3 | 0.21 (0.03 to 0.39) | 0%, 0 | 0.006 |
| Manual | 6 | 0.22 (-0.26 to 0.71) | 88%, 0.31 |  | 2 | -0.23 (-0.43 to -0.03) | 0%, 0 |  |
| N/S | 6 | 0.64 (0.27 to 1.01) | 71%, 0.14 |  | 2 | -0.02 (-0.46 to 0.43) | 0%, 0 |  |
| **US transducer frequency** |  |  |  |  |  |  |  |  |
| ≤ 12 MHz | 10 | 0.69 (0.45 to 0.93) | 57%, 0.07 | <0.001 | 3 | 0.21 (-0.06 to 0.48) | 0%, 0 | 0.253 |
| > 12 MHz | 4 | 0.03 (-0.5 to 0.56) | 90%, 0.26 |  | 2 | -0.06 (-0.66 to 0.54) | 77%, 0.14 |  |
| N/S | 2 | -0.06 (-0.25 to 0.12) | 0%, 0.00 |  | 2 | -0.04 (-0.34 to 0.27) | 69%, 0.03 |  |
| **Age** |  |  |  |  |  |  |  |  |
| 0 to 1 years | 4 | 0.63 (-0.02 to 1.27) | 91%, 0.39 | 0.002 | 1 | 0.03 (-0.53 to 0.59) | - | 0.957 |
| 2 to 16 years | 12 | 0.31 (0.06 to 0.55) | 77%, 0.13 |  | 6 | 0.02 (-0.2 to 0.25) | 58%, 0.04 |  |
| **N participants** |  |  |  |  |  |  |  |  |
| < 200 | 11 | 0.44 (0.05 to 0.84) | 83%, 0.35 | 0.699 | 4 | -0.04 (-0.34 to 0.27) | 32%, 0.03 | 0.716 |
| ≥ 200 | 5 | 0.34 (0.03 to 0.65) | 86%, 0.11 |  | 3 | 0.08 (-0.23 to 0.38) | 73%, 0.05 |  |
| Abbreviations: CIMT, carotid intima-media thickness; N, number; N/S, not specified; I2 and tau2 assess within-group heterogeneity; p-value assesses between-group heterogeneity. | | | | | | | | |

**Supplemental Table S7. Criteria for study quality assessment**

| INTERVENTIONAL STUDIES | |
| --- | --- |
| Random sequence generation | Selection bias (biased allocation to interventions) due to inadequate generation of a randomized sequence.  Criteria   - high: the random component in the sequence generation process was referring to a random number table, using a computer random number generator, coin tossing, shuffling cards or envelopes, throwing dice, or drawing of lots - low: non-random component in the sequence generation process such as generation by odd or even date of birth, some rule based on date (or day) of admission, some rule based on hospital or clinic record number, or allocation by judgment of the clinician, preference of the participant, based on the results of a laboratory test or a series of tests, or availability of the intervention - unclear: insufficient information about the sequence generation process to permit judgment of ‘low’ or ‘high’ quality |
| Allocation concealment | Selection bias (biased allocation to interventions) due to inadequate concealment of allocations prior to assignment.  Criteria   - high: assignment could not be foreseen by participants and investigators due to central allocation, sequentially numbered drug container of identical appearance, sequentially numbered opaque sealed envelopes - low: assignment could have been foreseen by participants or investigators due to open random allocation schedule (e.g. list of random numbers), assignment envelopes without appropriate safeguards, alternation or rotation, date of birth, case record number, or any other explicitly unconcealed procedure - unclear: insufficient information to permit judgment of ‘low’ or ‘high’ quality |
| Blinding participants and personnel | Performance bias due to knowledge of the allocated interventions by participants and personnel during the study.  Criteria   - high: no blinding or incomplete blinding, but the review authors judge that the outcome is not likely to be influenced by lack of blinding; or blinding of participants and key study personnel ensured, and unlikely that the blinding could have been broken - low: no blinding or incomplete blinding, and the outcome is likely to be influenced by lack of blinding; or blinding of key study participants and personnel attempted, but likely that the blinding could have been broken, and the outcome is likely to be influenced by lack of blinding. - unclear: insufficient information to permit judgment of ‘low’ or ‘high’ quality |
| Blinding outcome assessment | Detection bias due to knowledge of the allocated interventions by outcome assessors.  Criteria   - high: no blinding of outcome assessment, but the review authors judge that the outcome measurement is not likely to be influenced by lack of blinding; or blinding of outcome assessment ensured, and unlikely that the blinding could have been broken. - low: no blinding of outcome assessment, and the outcome measurement is likely to be influenced by lack of blinding; or blinding of outcome assessment, but likely that the blinding could have been broken, and the outcome measurement is likely to be influenced by lack of blinding. - unclear: insufficient information to permit judgment of ‘low’ or ‘high’ quality |
| Incomplete data outcome | Attrition bias due to amount, nature or handling of incomplete outcome data.  Criteria   - high: no missing outcome data; or reasons for missing outcome data unlikely to be related to true outcome (for survival data, censoring unlikely to be introducing bias); or missing outcome data balanced in numbers across intervention groups, with similar reasons for missing data across groups; or for dichotomous outcome data, the proportion of missing outcomes compared with observed event risk not enough to have a clinically relevant impact on the intervention effect estimate; or for continuous outcome data, plausible effect size (difference in means or standardized difference in means) among missing outcomes not enough to have a clinically relevant impact on observed effect size; or missing data have been imputed using appropriate methods - low: reason for missing outcome data likely to be related to true outcome, with either imbalance in numbers or reasons for missing data across intervention groups; or for dichotomous outcome data, the proportion of missing outcomes compared with observed event risk enough to induce clinically relevant bias in intervention effect estimate; or for continuous outcome data, plausible effect size (difference in means or standardized difference in means) among missing outcomes enough to induce clinically relevant bias in observed effect size; or ‘as-treated’ analysis done with substantial departure of the intervention received from that assigned at randomization or; or potentially inappropriate application of simple imputation - unclear: insufficient reporting of attrition/exclusions to permit judgment of ‘low’ or ‘high’ quality (e.g. number randomized not stated, no reasons for missing data provided) |
| Selective reporting | Reporting bias due to selective outcome reporting.  Criteria   - high: the study protocol is available and all of the study’s pre-specified (primary and secondary) outcomes that are of interest in the review have been reported in the pre-specified way; or the study protocol is not available but it is clear that the published reports include all expected outcomes, including those that were pre-specified (convincing text of this nature may be uncommon) - low: not all of the study’s pre-specified primary outcomes have been reported; or one or more primary outcomes is reported using measurements, analysis methods or subsets of the data (e.g. subscales) that were not pre-specified; or one or more reported primary outcomes were not pre-specified (unless clear justification for their reporting is provided, such as an unexpected adverse effect); or one or more outcomes of interest in the review are reported incompletely so that they cannot be entered in a meta-analysis or; or the study report fails to include results for a key outcome that would be expected to have been reported for such a study - unclear: insufficient information to permit judgment of ‘low’ or ‘high’ quality |
| Other bias | Bias due to problems not covered elsewhere in the table.  Criteria   - high: the study appears to be free of other sources of bias. - low: there is at least one important risk of bias, for example, the study had a potential source of bias related to the specific study design used; or has been claimed to have been fraudulent; or had some other problem - unclear: there may be a risk of bias, but there is either: insufficient information to assess whether an important risk of bias exists; or insufficient rationale or evidence that an identified problem will introduce bias. |
| OBSERVATIONAL STUDIES | |
| Selection of non-exposed | For both cohort and cross-sectional studies.  Criteria   - high: non-exposed drawn from the same source as the exposed cohort or study performed in a single pool of participants (i.e. when sampling of participants in the study is not based on the exposure status) - low: non-exposed drawn from a different source than the exposed cohort - unclear: insufficient information to permit judgement; no description of the derivation of the non-exposed |
| Ascertainment of outcome | For both cohort and cross-sectional studies.  Criteria   - high: blind assessment - low: non-blind assessment - unclear: insufficient information to permit judgement |
| Participation rate (adequacy of follow-up) | For cohort studies only.  Criteria   - high: complete follow-up or subjects lost to follow-up unlikely to introduce bias (i.e. small number lost- ≥ 80% follow-up or follow-up rate corresponding to < 80%, but subjects lost to follow-up were well described and did not differ substantially from those not lost to follow-up) - low: subjects lost to follow-up likely to introduce bias (subjects lost to follow-up differ substantially from those not lost to follow-up or follow-up rate corresponding to < 80% without further description of those lost to follow-up) - unclear: insufficient information to permit judgement |
| Participation rate (non-response rate) | For cross-sectional studies only.  Criteria   - high: non-response rate ≤ 20% - low: non-response rate ≥ 20% - unclear: insufficient information to permit judgement |
| Ascertainment of exposure | For both cohort and cross-sectional studies.  Ascertainment of exposure   - high: objective and/or reliable assessment method (e.g. reference to records, clinical assessments by study team) - low: method based on self-reporting (e.g. interview or questionnaire) without independent validation (that is, for example, without reference to another source, such as medical records or registries) - unclear: insufficient information to permit judgement |
| Same method of ascertainment for exposed and non-exposed | For both cohort and cross-sectional studies.  Same method of ascertainment for exposed and non-exposed?   - high: yes - low: no - unclear: insufficient information to permit judgement |
| Comparability | For both cohort and cross-sectional studies.  Comparability of exposed and non-exposed for child age and sex, on the basis of the design or analysis:   - high: study controls for these factors - low: study does not control for these factors - unclear: insufficient information to permit judgement |
| Note: high: high quality and low risk of bias; low: low quality and high risk of bias | |

**Supplemental Table S9. Assessment of study quality for observational studies included in meta-analyses.**

| Author, Year | Selection of non-exposed | Ascertain outcome | Participation rate | Type of exposure | Ascertainexposure | Same method for exposed and non-exposed | Comparability | CIMT acquisition  site | CIMT image analysis | CIMT reproducibility assessment |
| --- | --- | --- | --- | --- | --- | --- | --- | --- | --- | --- |
| Gale, 2006 | high | unclear | high | Maternal smoking in pregnancy | low | high | high | higher | higher | higher |
| Ayer, 2009 (a) | high | high | high | Birth weight | high | high | low | higher | higher | higher |
|  |  |  |  | Maternal diabetes in pregnancy | low | high | low |  |  |  |
| Ayer, 2011 | high | high | high | Maternal smoking in pregnancy | low | high | low | higher | higher | higher |
| Crispi, 2010 | high | high | high | Prematurity | high | high | low | higher | higher | higher |
|  |  |  |  | Small size for gestational age | high | high | high |  |  |  |
| Trevisanuto, 2010 | high | high | low | Small size for gestational age | unclear | unclear | low | higher | lower | higher |
| Geerts, 2012 | high | high | high | Maternal smoking in pregnancy | low | high | high | higher | higher | higher |
| Atabek, 2011 | high | high | unclear | Birth length | high | high | low | higher | lower | unclear |
|  |  |  |  | Birth weight | high | high | low |  |  |  |
|  |  |  |  | Birth head circumference | high | high | low |  |  |  |
|  |  |  |  | Maternal diabetes in pregnancy | unclear | unclear | low |  |  |  |
| Dratva, 2013 | high | unclear | unclear | Prematurity | high | high | low | higher | higher | higher |
| Schubert, 2013 | low | high | unclear | Prematurity | high | high | low | higher | higher | higher |
| Valenzuela-Alcaraz, 2019 | high | high | unclear | ART conception | high | high | low | higher | higher | higher |
| Morsing, 2014 | low | unclear | low | Prematurity | high | high | high | unclear | lower | unclear |
|  |  |  |  | Small size for gestational age | high | high | high |  |  |  |
| Stergiotou, 2014 | high | unclear | unclear | Small size for gestational age | high | high | high | higher | higher | higher |
| Gruszfeld, 2015 | high | high | low | Birth weight | high | high | low | higher | lower | lower |
| Sebastiani, 2016 | high | unclear | low | Small size for gestational age | high | high | high | higher | unclear | higher |
| Mohlkert, 2017 | low | high | high | Prematurity | high | high | high | higher | higher | unclear |
| Tzschoppe , 2017 | high | unclear | low | Small size for gestational age | high | high | low | unclear | unclear | unclear |
| Sebastiani, 2019 | high | unclear | low | Small size for gestational age | high | high | high | higher | unclear | higher |
| Sundholm, 2019 | low | high | low | Maternal diabetes in pregnancy | high | high | unclear | higher | unclear | higher |
| Jouret, 2011 | low | unclear | unclear | Small size for gestational age | high | unclear | high | unclear | unclear | unclear |
| Scherrer, 2012 | low | high | unclear | ART conception | unclear | unclear | high | unclear | higher | higher |
| de Arriba, 2013 | high | unclear | unclear | Birth length | unclear | unclear | low | higher | unclear | unclear |
|  |  |  |  | Birth weight | unclear | unclear | low |  |  |  |
|  |  |  |  | Small size for gestational age | unclear | unclear | low |  |  |  |
| Maurice, 2014 | high | unclear | unclear | Small size for gestational age | high | high | low | higher | higher | unclear |
| Xu, 2014 | unclear | high | unclear | ART conception | high | high | low | unclear | unclear | unclear |
| Sodhi, 2015 | high | high | unclear | Birth length | high | high | unclear | unclear | lower | lower |
|  |  |  |  | Birth weight | high | high | unclear |  |  |  |
|  |  |  |  | Birth head circumference | high | high | unclear |  |  |  |
|  |  |  |  | Small size for gestational age | high | high | low |  |  |  |
| Ciccone, 2016 | high | high | unclear | Prematurity | high | high | high | higher | unclear | unclear |
| Faienza, 2016 | low | unclear | unclear | Small size for gestational age | unclear | unclear | high | unclear | lower | higher |
| Olander, 2016 | high | unclear | unclear | Birth weight | high | high | low | higher | lower | higher |
|  |  |  |  | Birth head circumference | high | high | low |  |  |  |
|  |  |  |  | Small size for gestational age | high | high | low |  |  |  |
| Dilli, 2017 | high | high | unclear | Birth weight | high | high | low | higher | higher | higher |
|  |  |  |  | Small size for gestational age | high | high | low |  |  |  |
| Stock, 2018 | high | unclear | low | Prematurity | high | high | low | higher | lower | unclear |
|  |  |  |  | Small size for gestational age | high | high | low |  |  |  |
| Muñiz Fontán, 2019 | high | high | unclear | Small size for gestational age | unclear | unclear | high | higher | higher | unclear |
| Note: high: high quality and low risk of bias; low: low quality and high risk of bias; higher: higher CIMT quality (reliability); lower: lower CIMT quality (reliability)  Abbreviations: ART, assisted reproductive technology. | | | | | | | | | | |

**Supplemental Figure S2. Assessment of small-study effects, including publication bias, for each exposure type included in meta-analyses.** Funnel plots of standardized mean difference (binary exposure variable) or Fischer’s z correlation (continuous exposure variable) against its standard error. Abbreviations: ART, assisted reproductive technology. * p-value for small study effects from Egger’s test, computed only for the exposure types with 10 or more studies included in meta-analysis.

| **a) Small size for gestational age (16 studies) *** | **c) Birth weight (7 studies)** |
| --- | --- |
|  |  |
|  |  |
| **d) Birth length (3 studies)** | **e) Birth head circumference (3 studies)** |
|  |  |
|  |  |
| **f) Prematurity (7 studies)** | **g) ART conception (3 studies)** |
|  |  |
|  |  |
| **h) Maternal diabetes in pregnancy (3 studies)** | **i) Maternal smoking in pregnancy (3 studies)** |
|  |  |
|  |  |

**S3 Fig. Random-effects meta-regression examining the influence of sample size on the association of small size for gestational age with carotid intima-media thickness.** Bubble plot with fitted meta-regression line. The circles represent the association estimate (standardized mean difference) from each study, sized according to the random-effects weights. Number of studies: 16. Residual between-study variance (tau^2^): 0.21. Residual variation due to heterogeneity (I^2^_res_): 81%. Between-study variance explained by the covariate sample size (Adjusted R^2^): 2%.

REFERENCES

ALOE, A. M. 2015. Inaccuracy of regression results in replacing bivariate correlations. *Res Synth Methods,* 6**,** 21-7.

AMATO, M., MONTORSI, P., RAVANI, A., OLDANI, E., GALLI, S., RAVAGNANI, P. M., TREMOLI, E. & BALDASSARRE, D. 2007. Carotid intima-media thickness by B-mode ultrasound as surrogate of coronary atherosclerosis: correlation with quantitative coronary angiography and coronary intravascular ultrasound findings. *Eur Heart J,* 28**,** 2094-101.

AMRHEIN, V., GREENLAND, S. & MCSHANE, B. 2019. Scientists rise up against statistical significance. *Nature,* 567**,** 305-307.

BARKER, M., DOMBROWSKI, S. U., COLBOURN, T., FALL, C. H. D., KRIZNIK, N. M., LAWRENCE, W. T., NORRIS, S. A., NGAIZA, G., PATEL, D., SKORDIS-WORRALL, J., SNIEHOTTA, F. F., STEEGERS-THEUNISSEN, R., VOGEL, C., WOODS-TOWNSEND, K. & STEPHENSON, J. 2018. Intervention strategies to improve nutrition and health behaviours before conception. *The Lancet,* 391**,** 1853-1864.

BORENSTEIN, M., HEDGES, L. V., HIGGINS, J. P. T. & ROTHSTEIN, H. R. 2009. Introduction to Meta‐Analysis. *John Wiley & Sons, Ltd*.

BOTS, M. L., EVANS, G. W., RILEY, W. A. & GROBBEE, D. E. 2003. Carotid intima-media thickness measurements in intervention studies: design options, progression rates, and sample size considerations: a point of view. *Stroke,* 34**,** 2985-94.

CHAMBERLAIN, C., O'MARA‐EVES, A., PORTER, J., COLEMAN, T., PERLEN, S. M., THOMAS, J. & MCKENZIE, J. E. 2017. Psychosocial interventions for supporting women to stop smoking in pregnancy. *Cochrane Database of Systematic Reviews*.

CRISPI, F., BIJNENS, B., FIGUERAS, F., BARTRONS, J., EIXARCH, E., LE NOBLE, F., AHMED, A. & GRATACOS, E. 2010. Fetal growth restriction results in remodeled and less efficient hearts in children. *Circulation,* 121**,** 2427-36.

DALLA POZZA, R., EHRINGER-SCHETITSKA, D., FRITSCH, P., JOKINEN, E., PETROPOULOS, A. & OBERHOFFER, R. 2015. Intima media thickness measurement in children: A statement from the Association for European Paediatric Cardiology (AEPC) Working Group on Cardiovascular Prevention endorsed by the Association for European Paediatric Cardiology. *Atherosclerosis,* 238**,** 380-7.

DE GROOT, E., HOVINGH, G. K., WIEGMAN, A., DURIEZ, P., SMIT, A. J., FRUCHART, J. C. & KASTELEIN, J. J. 2004. Measurement of arterial wall thickness as a surrogate marker for atherosclerosis. *Circulation,* 109**,** Iii33-8.

DRATVA, J., BRETON, C. V., HODIS, H. N., MAC, K. W. J., SALAM, M. T., ZEMP, E., GILLILAND, F., KUENZLI, N. & AVOL, E. 2013. Birth weight and carotid artery intima-media thickness. *Journal of Pediatrics,* 162**,** 906-911.e2.

EIKENDAL, A. L., GROENEWEGEN, K. A., ANDERSON, T. J., BRITTON, A. R., ENGSTROM, G., EVANS, G. W., DE GRAAF, J., GROBBEE, D. E., HEDBLAD, B., HOLEWIJN, S., IKEDA, A., KITAGAWA, K., KITAMURA, A., LONN, E. M., LORENZ, M. W., MATHIESEN, E. B., NIJPELS, G., DEKKER, J. M., OKAZAKI, S., O'LEARY, D. H., POLAK, J. F., PRICE, J. F., ROBERTSON, C., REMBOLD, C. M., ROSVALL, M., RUNDEK, T., SALONEN, J. T., SITZER, M., STEHOUWER, C. D., HOEFER, I. E., PETERS, S. A., BOTS, M. L. & DEN RUIJTER, H. M. 2015. Common carotid intima-media thickness relates to cardiovascular events in adults aged <45 years. *Hypertension,* 65**,** 707-13.

EPURE, A. M., LEYVRAZ, M., MIVELAZ, Y., DI BERNARDO, S., DA COSTA, B. R., CHIOLERO, A. & SEKARSKI, N. 2018. Risk factors and determinants of carotid intima-media thickness in children: protocol for a systematic review and meta-analysis. *BMJ Open,* 8**,** e019644.

FLEMING, T. P., WATKINS, A. J., VELAZQUEZ, M. A., MATHERS, J. C., PRENTICE, A. M., STEPHENSON, J., BARKER, M., SAFFERY, R., YAJNIK, C. S., ECKERT, J. J., HANSON, M. A., FORRESTER, T., GLUCKMAN, P. D. & GODFREY, K. M. 2018. Origins of lifetime health around the time of conception: causes and consequences. *Lancet,* 391**,** 1842-1852.

GIANNOPOULOU, E. Z., DOUNDOULAKIS, I., ANTZA, C., CHRISTOFORIDIS, A., HAIDICH, A. B., KOTSIS, V. & STABOULI, S. 2019. Subclinical arterial damage in children and adolescents with type 1 diabetes: A systematic review and meta-analysis. *Pediatr Diabetes,* 20**,** 668-677.

HANSON, M. & GLUCKMAN, P. 2011. Developmental origins of noncommunicable disease: population and public health implications. *Am J Clin Nutr,* 94**,** 1754s-1758s.

HANSON, M. A. & GLUCKMAN, P. D. 2014. Early developmental conditioning of later health and disease: physiology or pathophysiology? *Physiol Rev,* 94**,** 1027-76.

HIGGINS, J. P. T. & GREEN, S. 2011. Cochrane Handbook for Systematic Reviews of Interventions Version 5.1.0 [updated March 2011] The Cochrane Collaboration, 2011.

HUXLEY, R. R., SHIELL, A. W. & LAW, C. M. 2000. The role of size at birth and postnatal catch-up growth in determining systolic blood pressure: a systematic review of the literature. *J Hypertens,* 18**,** 815-31.

JOURET, B., DULAC, Y., BASSIL ETER, R., TAKTAK, A., CRISTINI, C., LOUNIS, N., MOLINAS, C., SALLES, J. P., ARNAUD, C., ACAR, P. & TAUBER, M. 2011. Endothelial function and mechanical arterial properties in children born small for gestational age: comparison with obese children. *Horm Res Paediatr,* 76**,** 240-7.

LEE, A. C., KOZUKI, N., COUSENS, S., STEVENS, G. A., BLENCOWE, H., SILVEIRA, M. F., SANIA, A., ROSEN, H. E., SCHMIEGELOW, C., ADAIR, L. S., BAQUI, A. H., BARROS, F. C., BHUTTA, Z. A., CAULFIELD, L. E., CHRISTIAN, P., CLARKE, S. E., FAWZI, W., GONZALEZ, R., HUMPHREY, J., HUYBREGTS, L., KARIUKI, S., KOLSTEREN, P., LUSINGU, J., MANANDHAR, D., MONGKOLCHATI, A., MULLANY, L. C., NDYOMUGYENYI, R., NIEN, J. K., ROBERFROID, D., SAVILLE, N., TERLOUW, D. J., TIELSCH, J. M., VICTORA, C. G., VELAPHI, S. C., WATSON-JONES, D., WILLEY, B. A., EZZATI, M., LAWN, J. E., BLACK, R. E. & KATZ, J. 2017. Estimates of burden and consequences of infants born small for gestational age in low and middle income countries with INTERGROWTH-21(st) standard: analysis of CHERG datasets. *Bmj,* 358**,** j3677.

LIBERATI, A., ALTMAN, D. G., TETZLAFF, J., MULROW, C., GØTZSCHE, P. C., IOANNIDIS, J. P., CLARKE, M., DEVEREAUX, P. J., KLEIJNEN, J. & MOHER, D. 2009. The PRISMA statement for reporting systematic reviews and meta-analyses of studies that evaluate health care interventions: explanation and elaboration. *PLoS Med,* 6**,** e1000100.

LIPSEY, M. W. & WILSON, D. B. 2000. *Practical Meta-Analysis*, SAGE Publications Inc.

LORENZ, M. W., MARKUS, H. S., BOTS, M. L., ROSVALL, M. & SITZER, M. 2007. Prediction of clinical cardiovascular events with carotid intima-media thickness: a systematic review and meta-analysis. *Circulation,* 115**,** 459-67.

MIVELAZ, Y., DI BERNARDO, S., BOULOS KSONTINI, T., PRSA, M., VIAL, Y., CHIOLERO, A. & SEKARSKI, N. 2016. Feasibility and reliability of carotid intima-media thickness measurements in nonsedated infants. *J Hypertens,* 34**,** 2227-32.

MOORE, T. G., AREFADIB, N., DEERY, A., KEYES, M. & WEST, S. 2017. The first thousand days: an evidence paper- summary. *Parkville, Victoria: Centre for Community Child Health, Murdoch Children's Research Institute*.

OREN, A., VOS, L. E., UITERWAAL, C. S., GORISSEN, W. H., GROBBEE, D. E. & BOTS, M. L. 2004. Birth weight and carotid intima-media thickness: new perspectives from the atherosclerosis risk in young adults (ARYA) study. *Ann Epidemiol,* 14**,** 8-16.

PIGNOLI, P., TREMOLI, E., POLI, A., ORESTE, P. & PAOLETTI, R. 1986. Intimal plus medial thickness of the arterial wall: a direct measurement with ultrasound imaging. *Circulation,* 74**,** 1399-406.

POTTER, K., REED, C. J., GREEN, D. J., HANKEY, G. J. & ARNOLDA, L. F. 2008. Ultrasound settings significantly alter arterial lumen and wall thickness measurements. *Cardiovasc Ultrasound,* 6**,** 6.

RISNES, K. R., VATTEN, L. J., BAKER, J. L., JAMESON, K., SOVIO, U., KAJANTIE, E., OSLER, M., MORLEY, R., JOKELA, M., PAINTER, R. C., SUNDH, V., JACOBSEN, G. W., ERIKSSON, J. G., SORENSEN, T. I. & BRACKEN, M. B. 2011. Birthweight and mortality in adulthood: a systematic review and meta-analysis. *Int J Epidemiol,* 40**,** 647-61.

SARKOLA, T., REDINGTON, A., KEELEY, F., BRADLEY, T. & JAEGGI, E. 2010. Transcutaneous very-high-resolution ultrasound to quantify arterial wall layers of muscular and elastic arteries: validation of a method. *Atherosclerosis,* 212**,** 516-23.

SARKOLA, T., SLORACH, C., HUI, W., BRADLEY, T. J., REDINGTON, A. N. & JAEGGI, E. 2012. Transcutaneous very-high resolution ultrasound for the quantification of carotid arterial intima-media thickness in children - feasibility and comparison with conventional high resolution vascular ultrasound imaging. *Atherosclerosis,* 224**,** 102-7.

SKILTON, M. R., VIIKARI, J. S., JUONALA, M., LAITINEN, T., LEHTIMAKI, T., TAITTONEN, L., KAHONEN, M., CELERMAJER, D. S. & RAITAKARI, O. T. 2011. Fetal growth and preterm birth influence cardiovascular risk factors and arterial health in young adults: the Cardiovascular Risk in Young Finns Study. *Arterioscler Thromb Vasc Biol,* 31**,** 2975-81.

STEIN, J. H., KORCARZ, C. E., HURST, R. T., LONN, E., KENDALL, C. B., MOHLER, E. R., NAJJAR, S. S., REMBOLD, C. M. & POST, W. S. 2008. Use of carotid ultrasound to identify subclinical vascular disease and evaluate cardiovascular disease risk: a consensus statement from the American Society of Echocardiography Carotid Intima-Media Thickness Task Force. Endorsed by the Society for Vascular Medicine. *J Am Soc Echocardiogr,* 21**,** 93-111; quiz 189-90.

STOCK, K., SCHMID, A., GRIESMAIER, E., GANDE, N., HOCHMAYR, C., KNOFLACH, M., KIECHL-KOHLENDORFER, U., BERNAR, B., WINDER, B., REITER, C., BURGER, C., KLINGENSCHMID, J., MARXER, J., ASARE, M., BOCK-BARTL, M., KOTHMAYER, M., BOHL, M., PIRCHER, M., GEIGER, R., KIECHL, S. & KIECHL, S. J. 2018. The Impact of Being Born Preterm or Small for Gestational Age on Early Vascular Aging in Adolescents. *Journal of Pediatrics,* 201**,** 49-54.e1.

SUNDHOLM, J. K., OLANDER, R. F., OJALA, T. H., ANDERSSON, S. & SARKOLA, T. 2015. Feasibility and precision of transcutaneous very-high resolution ultrasound for quantification of arterial structures in human neonates - comparison with conventional high resolution vascular ultrasound imaging. *Atherosclerosis,* 239**,** 523-7.

SUNDHOLM, J. K. M., PAETAU, A., ALBÄCK, A., PETTERSSON, T. & SARKOLA, T. 2019. Non-Invasive Vascular Very-High Resolution Ultrasound to Quantify Artery Intima Layer Thickness: Validation of the Four-Line Pattern. *Ultrasound Med Biol,* 45**,** 2010-2018.

TANG, R., HENNIG, M., BOND, M. G., HOLLWECK, R., MANCIA, G. & ZANCHETTI, A. 2005. Quality control of B-mode ultrasonic measurement of carotid artery intima-media thickness: the European Lacidipine Study on Atherosclerosis. *J Hypertens,* 23**,** 1047-54.

TIAN, G., GUO, C., LI, Q., LIU, Y., SUN, X., YIN, Z., LI, H., CHEN, X., LIU, X., ZHANG, D., CHENG, C., LIU, L., LIU, F., ZHOU, Q., WANG, C., LI, L., WANG, B., ZHAO, Y., LIU, D., ZHANG, M. & HU, D. 2019. Birth weight and risk of type 2 diabetes: A dose-response meta-analysis of cohort studies. *Diabetes Metab Res Rev,* 35**,** e3144.

TORIGOE, T., DALLAIRE, F., SLORACH, C., CARDINAL, M. P., HUI, W., BRADLEY, T. J., SARKOLA, T., MERTENS, L. & JAEGGI, E. 2020. New Comprehensive Reference Values for Arterial Vascular Parameters in Children. *J Am Soc Echocardiogr*.

TOUBOUL, P. J., HENNERICI, M. G., MEAIRS, S., ADAMS, H., AMARENCO, P., BORNSTEIN, N., CSIBA, L., DESVARIEUX, M., EBRAHIM, S., HERNANDEZ HERNANDEZ, R., JAFF, M., KOWNATOR, S., NAQVI, T., PRATI, P., RUNDEK, T., SITZER, M., SCHMINKE, U., TARDIF, J. C., TAYLOR, A., VICAUT, E. & WOO, K. S. 2012. Mannheim carotid intima-media thickness and plaque consensus (2004-2006-2011). An update on behalf of the advisory board of the 3rd, 4th and 5th watching the risk symposia, at the 13th, 15th and 20th European Stroke Conferences, Mannheim, Germany, 2004, Brussels, Belgium, 2006, and Hamburg, Germany, 2011. *Cerebrovasc Dis,* 34**,** 290-6.

URBINA, E. M., WILLIAMS, R. V., ALPERT, B. S., COLLINS, R. T., DANIELS, S. R., HAYMAN, L., JACOBSON, M., MAHONEY, L., MIETUS-SNYDER, M., ROCCHINI, A., STEINBERGER, J. & MCCRINDLE, B. 2009. Noninvasive assessment of subclinical atherosclerosis in children and adolescents: recommendations for standard assessment for clinical research: a scientific statement from the American Heart Association. *Hypertension,* 54**,** 919-50.

VISENTIN, S., GRUMOLATO, F., NARDELLI, G. B., DI CAMILLO, B., GRISAN, E. & COSMI, E. 2014. Early origins of adult disease: low birth weight and vascular remodeling. *Atherosclerosis,* 237**,** 391-9.

WAN, X., WANG, W., LIU, J. & TONG, T. 2014. Estimating the sample mean and standard deviation from the sample size, median, range and/or interquartile range. *BMC Med Res Methodol,* 14**,** 135.

WOLLMANN, H. A. 1998. Intrauterine growth restriction: definition and etiology. *Horm Res,* 49 Suppl 2**,** 1-6.

WONG, M., EDELSTEIN, J., WOLLMAN, J. & BOND, M. G. 1993. Ultrasonic-pathological comparison of the human arterial wall. Verification of intima-media thickness. *Arterioscler Thromb,* 13**,** 482-6.
