# Supplementary material for: Risk factors during first 1,000 days of life for carotid intima-media thickness in infants, children, and adolescents: A systematic review with meta-analyses
Source: PLoS Med. 2020 Nov 23;17(11):e1003414. doi: 10.1371/journal.pmed.1003414 (PMC7682901; doi:10.1371/journal.pmed.1003414)
Supplement: Attachment — Submitted filename: response_to_editors_and_reviewers_corrections.docx [file pmed.1003414.s016.docx]

Dear Editors,

We are submitting a new version of the manuscript that contains the revision with the corresponding corrections that were sent to us on September 29 2020.

We have made all changes as requested. We believe, however, that there is a confusion between the p-value related to the pooled association estimate and the p-value related to the heterogeneity between studies in a meta-analysis, as explained below at comment 1d. We updated our Tables 3 and 4 and in-text data corresponding to results from meta-analyses to include the p-value related to the pooled association estimate for each risk factor that was meta-analyzed. Thus, all p-values for the pooled association estimates reported in the abstract are now found in the main text too. We also clarified this in the Methods: “The uncertainty around the point estimate is comprised in the accompanying 95% CI, which is reported along the corresponding p-value”.

This revision was initially submitted on September 24, but it was returned on September 29 2020 to enable us to make the corrections listed herein. The revision files that we initially submitted on September 24 are listed as file type ‘Other’ in the File Inventory and described as “submitted on 24 Sep 2020” in the Description field. The files listed as ‘Marked Up Manuscript’, ‘Manuscript’, and ‘Response to Reviewers’ in the File Inventory correspond to your latest request, i.e. the corrections on the revision submitted on September 24.

We also solved the remaining production issues, i.e. we formatted Figs 2, 3, 4 as per your technical specificities. These figures were performed with Stata and exported as eps, then uploaded and transformed to tiff in PACE. We submit them in a tiff format.

Sincerely,

Dr. Adina Mihaela Epure, Prof Arnaud Chiolero, and Prof. Nicole Sekarski

CORRECTIONS REQUIRED BY THE EDITORS

1. Abstract:

a. Lines 32-33: Please include a more concrete list of the (main) risk factors/exposures tested.

**Response:** We added: “Observational and interventional studies evaluating factors at the individual, familial, or environmental levels, for instance, size at birth, gestational age, breastfeeding, mode of conception, gestational diabetes or smoking, were included”.

b. Line 59: Please still begin this section with: “In our meta-analyses, we found that…”

**Response:** We revised accordingly.

c. Lines 60-62: We would still ask that you rephrase this non-significant result: “Conception through ART and smoking during pregnancy were not significantly associated with increased CIMT, but the imprecision around the estimates was high.”

**Response:** We agree to highlight the absence of statistical significance. We rephrased: “Conception through ART and smoking during pregnancy were not statistically significantly associated with increased CIMT, with a high imprecision around the estimates”.

d. Please correct here to match Table 3 (gives p as <0.001): “Children conceived through assisted reproductive technologies (ART) (3 studies; 323 participants; pooled SMD: 0.78 (95% CI: -0.20 to 1.75, p-value: 0.120, I2: 94%) …”

**Response:** We believe that there is a confusion between the p-values related to the pooled association estimate and the p-values related to the heterogeneity between studies in a meta-analysis. In this specific case, the p-value of 0.120 corresponds to the pooled association estimate. This is the p-value that is linked with the 95% confidence interval, which we were asked to report for all results presented in the abstract in your Decision email dated September 17 2020. The p-value of <0.001 reported in Table 3 (under the column *Heterogeneity*) corresponds to the heterogeneity between studies. To avoid confusion, we kept in the abstract the p-value of 0.120, which is related to the pooled association estimate for ART, and we also added it in Table 3 (under the column *Association estimate*), as well as in the Results section of the main text.

e. Please correct here to match Table 3 (gives p as 0.38): “…exposed to maternal smoking during pregnancy (3 studies; 909 participants; pooled SMD: 0.12 (95% CI: -0.06 to 0.30, p-value: 0.205, I2: 0%) …”

**Response:** This is a similar situation to the one at point 1d and we adopted the same approach.

f. Please present the following data in your main Results (and also mention which data table contains these values): “When restricted to studies of higher quality of CIMT measurement, this relationship was stronger (3 studies; 461 participants; pooled SMD: 0.64 (95% CI: 0.09 to 1.19, p-value: 0.024, I2: 86%).”

**Response:** We included this data in the text of the main Results, next to the corresponding statement (subheading *Risk factors at the child level*, paragraph 2). This data is presented in Fig 2B (Higher CIMT reliability), which has been cited in the text.

2. Author summary: Lines 78-80: Please rephrase to: “Risk factors in the first 1,000 days of life, particularly poor fetal growth, are associated with increased CIMT in infants, children, and adolescents.”

**Response:** We revised accordingly.

3. Discussion: Lines 547-549: Please rephrase to: “Poor fetal growth had the most consistent and strongest association with increased CIMT, while ART conception and maternal smoking during pregnancy were not significantly associated.”

**Response:** We agree to highlight the absence of statistical significance. We rephrased where you indicated, i.e. Discussion section, *Conclusion*: “Poor fetal growth had the most consistent and strongest association with increased CIMT, while ART conception and maternal smoking during pregnancy were not statistically significantly associated.”. In addition, we rephrased a similar statement in the Discussion section, *Main findings,* in the same way: “Conception through ART and maternal smoking during pregnancy were not statistically significantly associated with increased CIMT”.
